# Bioactive Compounds from Marine Heterobranchs

**DOI:** 10.3390/md18120657

**Published:** 2020-12-21

**Authors:** Conxita Avila, Carlos Angulo-Preckler

**Affiliations:** 1 Department of Evolutionary Biology, Ecology, and Environmental Sciences, Biodiversity Research Institute (IrBIO), Faculty of Biology, University of Barcelona, Av. Diagonal 643, 08028 Barcelona, Catalonia, Spain; carlos.a.preckler@uit.no; 2 Norwegian College of Fishery Science, UiT The Arctic University of Norway, Hansine Hansens veg 18, 9019 Tromsø, Norway

**Keywords:** marine natural products, Mollusca, Gastropoda, chemical ecology

## Abstract

The natural products of heterobranch molluscs display a huge variability both in structure and in their bioactivity. Despite the considerable lack of information, it can be observed from the recent literature that this group of animals possesses an astonishing arsenal of molecules from different origins that provide the molluscs with potent chemicals that are ecologically and pharmacologically relevant. In this review, we analyze the bioactivity of more than 450 compounds from ca. 400 species of heterobranch molluscs that are useful for the snails to protect themselves in different ways and/or that may be useful to us because of their pharmacological activities. Their ecological activities include predator avoidance, toxicity, antimicrobials, antifouling, trail-following and alarm pheromones, sunscreens and UV protection, tissue regeneration, and others. The most studied ecological activity is predation avoidance, followed by toxicity. Their pharmacological activities consist of cytotoxicity and antitumoral activity; antibiotic, antiparasitic, antiviral, and anti-inflammatory activity; and activity against neurodegenerative diseases and others. The most studied pharmacological activities are cytotoxicity and anticancer activities, followed by antibiotic activity. Overall, it can be observed that heterobranch molluscs are extremely interesting in regard to the study of marine natural products in terms of both chemical ecology and biotechnology studies, providing many leads for further detailed research in these fields in the near future.

## 1. Background

Marine heterobranch molluscs are a well-known source of marine natural products (MNPs) that have been studied in depth over the years [1,2,3]. MNPs from heterobranchs show an amazing structural diversity and display a wide variety of biological activities, as reported in previous reviews [1,2,3,4]. In general, MNPs have been demonstrated to be crucial in many ecological interactions among marine organisms, regulating several aspects of reproduction, development, settlement, growth, defense, and others [2,5,6,7]. Some general reviews have reported a significant amount of detailed information on the structure of MNPs, marine chemical ecology, and marine chemistry, or have analyzed some particular mollusc compounds [4,8,9,10,11,12,13,14,15,16,17,18]. The yearly reports by Blunt and collaborators [5,6] have provided very accurate information on new marine natural products. Previous reviews have also dealt with the different chemical structures found in heterobranchs, the origin and anatomical allocation of their compounds, their biosynthesis, biogeography, and their evolutionary patterns [1,2,19,20,21,22,23,24,25,26,27,28,29]. Therefore, all of these topics will not be considered again here.

Furthermore, MNPs have been described to be potentially useful as drugs, and some of them are already available on the market [7,8,10,12,30,31,32,33]. Remarkably, many MNPs possess unique chemical structures that are totally absent in terrestrial or freshwater environments [32,34,35,36,37]. Five drugs, at least, have been isolated from marine invertebrates and are approved for different (mostly anticancer) purposes, including cytarabine (Ara-C), eribulin mesylate, ziconotide, brentuximab vedotin, and trabectedin, obtained from two sponges, two molluscs, and a tunicate, respectively [31,33,38]. These molecules include very different chemical structures, from nucleosides to peptides, alkaloids, macrolides, and antibody–drug conjugates (ADCs). Many other compounds are currently in phase III, phase II, and phase I clinical trials, including several heterobranch compounds, and could soon be on the market [31]. Moreover, many studies deal with MNPs bioactivity, mechanisms of action, virtual screening, synthesis, derivatives, ADMET (absorption, distribution, metabolism, excretion, and toxicity), and others in an attempt to increase the chances of finding new useful drugs [31,32,33,34,35,36,37,38,39,40,41,42,43]. Some databases are also very good tools to search the details of MNPs described to date, such as MarinLit (http://pubs.rsc.org/marinlit/). In cancer research, for example, NPs are considered very relevant as potential drug leads, and approximately 80% of the approved chemotherapeutic drugs and more than 50% of all drugs are based on bioactive natural products, while almost 90% of human diseases are treated with natural products or their derivatives [39,40,41,42,43]. Thus, many MNPs are being tested as antitumor agents because of their potent growth inhibition against human tumor cells, both in vitro and in vivo in murine models (and others), as well as in cancer clinical trials [39,42,43].

In fact, marine organisms are still considered an underexplored source of NPs, displaying specific biological activities, with biomedically interesting applications to be potentially used as drugs [2,5,6,8,10,29,30,31,44]. Many compounds found in heterobranchs are also promising drugs and are being tested under clinical trials [36,43,45,46]. However, as far as we know, there has not yet been a comprehensive published review on the bioactivity of MNPs from heterobranch molluscs, despite the fact that this is one of the most chemodiverse invertebrate groups [2,4]. For this reason, we summarize here all the ecological and pharmacological activities reported in heterobranch molluscs, trying to emphasize in the assays carried out, whether they are or not ecologically and biomedically significant, and their potential interest, since it seemed timely and necessary now. As previously mentioned, this review does not cover other ecological or evolutionary aspects that are already covered in previous reviews [1,2], nor the chemical synthesis of the MNPs. The aim of this review is, therefore, to showcase the main ecological and pharmacological bioactivities of the chemical compounds found in heterobranch molluscs, describing in which groups they are found and their particular bioactivities with all of the information we have been able to compile up to June 2020. 

Heterobranch molluscs are soft-bodied and mostly shell-less animals that live all around the planet at all latitudes and depths [2]. These animals are often protected by chemical strategies, although they may also present behavioral and/or morphological strategies to combine them with [1]. As a result of the most recent evolutionary, phylogenetic, and taxonomical studies on the group, heterobranch gastropods now comprise the classical “Opisthobranchia” and the marine “Pulmonata” together with several other groups, reaching a total of more than 33,000 species, although the most well-known groups account for only ca. 9000 species [47,48,49,50,51]. Among these, only about 400 species have been chemically analyzed, and, therefore, a lot of compounds remain to be potentially discovered [1,2,5,6,52]. Among the chemically studied heterobranch species, a wide variety of compounds has been described, many of them being bioactive at the ecological and/or pharmacological level [2,8]. At the ecological level, some NPs are used for protection against potential predators and competitors, enhancing their ecological performance, while others may have a role in their reproduction, development, growth, and feeding behavior [1,2,8]. In heterobranch molluscs, NPs may be de novo biosynthesized by the animals, obtained from their diet (biotransformed or not), or perhaps even produced by symbionts [1,2]. In any case, all of them are considered in this review because they are found in and used by the molluscs.

This review analyzes the bioactive compounds by activity (ecological and pharmacological, and different subtopics within them) and by taxonomical groups. Heterobranchs classically include eight major taxa: Nudibranchia, Pleurobranchoidea (or Pleurobranchida), Tylodinoidea (or Umbraculida), Cephalaspidea, Anaspidea (or Aplysiida), Pteropoda, Sacoglossa, and Pulmonata (Table 1) [47,48,49,50]. All of these taxa have different morphological and anatomical characteristics; different diet, behavioral, and ecological traits; and different chemical strategies [1,2]. Nudibranchs (sea slugs) are carnivorous and comprise Doridacea, Dendronotida, Euarminida, and Aeolidida, and are considered the most diverse group, with Doridacea feeding on porifera (sponges), bryozoans, tunicates, or other “opisthobranchs”, Dendronotids prey on cnidarians (usually octocorals or hydrozoans) or some small animals (crustaceans or turbellarians), Euarminida feed on octocoral cnidarians or bryozoans, and Aeolidida are mainly cnidarian feeders [1]. All of them lack a shell in adult stage, and they possess interesting chemistry that may be de novo biosynthesized or obtained from their diet of the above-mentioned prey [1,2]. Pleurobranchoidea (side-gill slugs) are usually ascidian feeders or generalist scavengers, while Tylodinoidea (false limpets) feed on sponges, and Cephalaspidea (head-shielded slugs and snails) may be algal feeders or voracious predators of other animals (other “opisthobranchs”, including other cephalaspideans), sponges, annelids, and others [1]. Anaspideans (sea hares) are herbivorous, feeding on different kinds of algae, but also on sea grasses, or even cyanobacteria. On the other hand, pelagic Pteropods (sea angels) are planktonic and feed on phytoplankton or other pteropods, while Sacoglossans and Pulmonates are herbivorous that feed on different types of algae [1,2].

## 2. Ecological Activity

### 2.1. Predation

**Figure 1:**
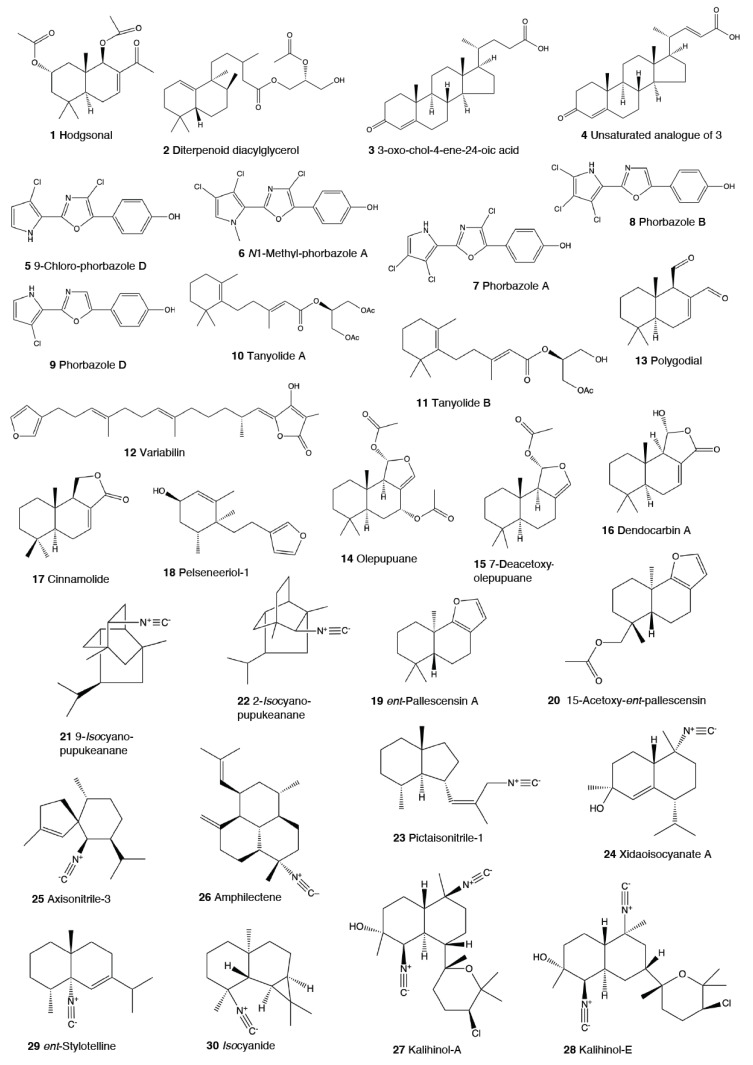
Structures of selected compounds used against predation in some Doridacea. These molecules may also display other activities, as reported in the text.

**Figure 2:**
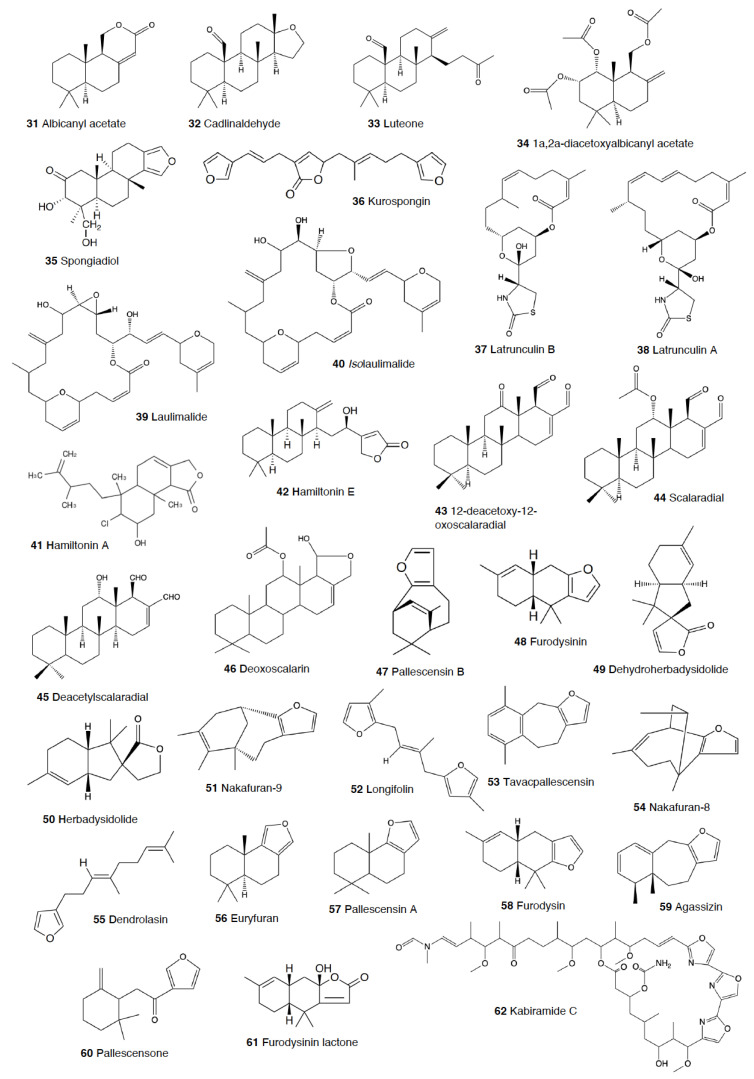
Structures of selected compounds used against predation in some Doridacea. These molecules may also display other activities, as reported in the text.

**Figure 3:**
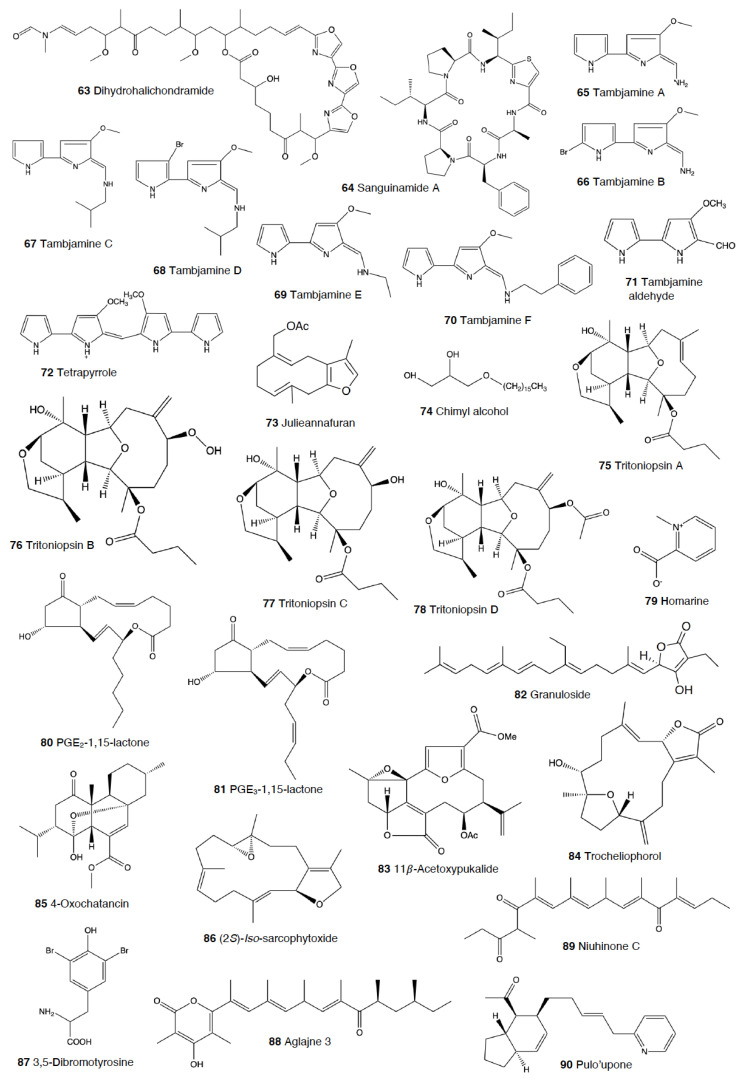
Structures of selected compounds used against predation in some Doridacea, Dendronotida, Euarminida, Aeolidida, Pleurobranchoidea, Tylodinoidea, and some Cephalaspidea. These molecules may also display other activities, as reported in the text.

**Figure 4:**
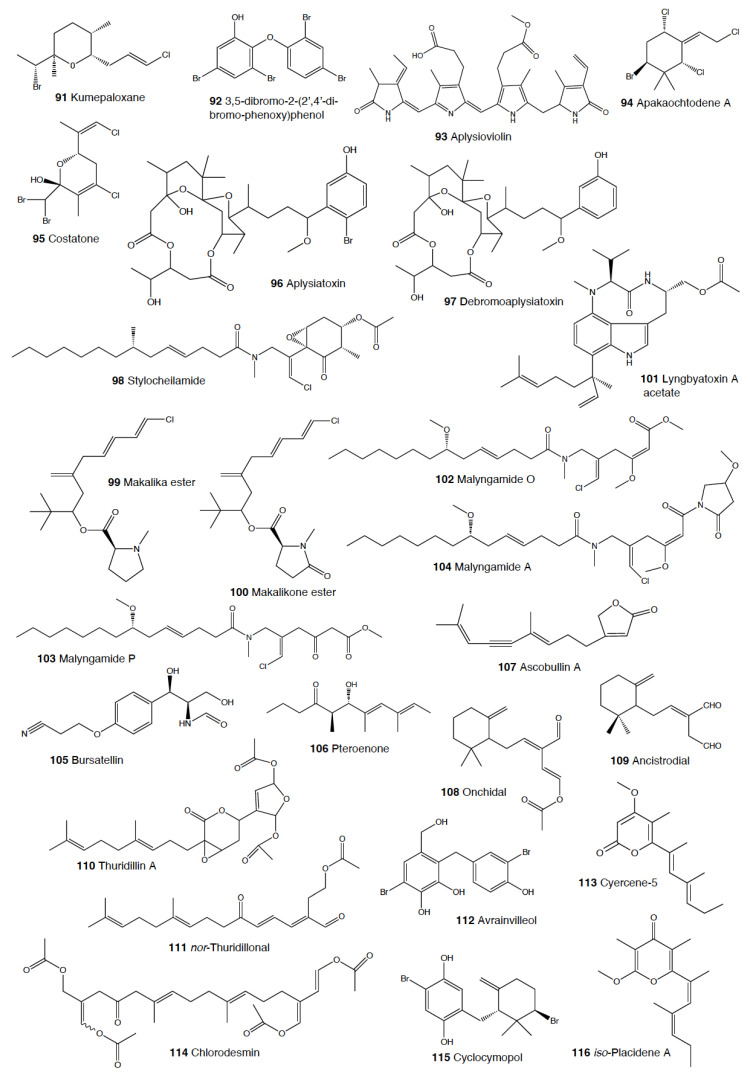
Structures of selected compounds used against predation in some Cephalaspidea, Anaspidea, Pteropoda, and Sacoglossa. These molecules may also display other activities, as reported in the text.

**Figure 5:**
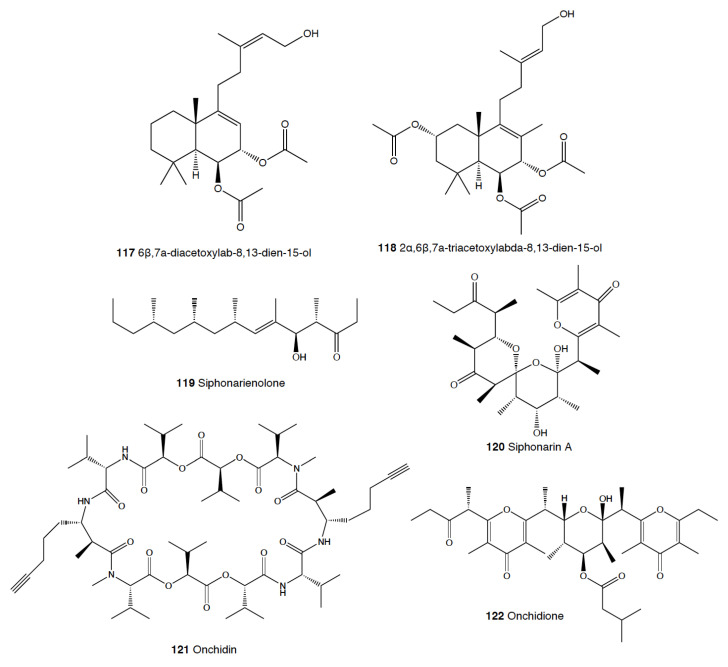
Structures of selected compounds used against predation in Pulmonata. These molecules may also display other activities, as reported in the text.

Heterobranch mollusc are protected against predation by a vast array of defensive strategies, many of which are combined with or include the use of natural products (Figure 1, Figure 2, Figure 3, Figure 4 and Figure 5) [2]. These chemical strategies may, in fact, be useful against many different kinds of predators, which can usually be grouped into three main types: fish, crabs, and sea stars, although other potential predators, such as anemones, sea spiders, etc., have also been reported (Table 2) [1,2]. Whether defensive strategies used against one predator are also effective against another potential predator is seldom reported in the literature. Furthermore, when laboratory assays are carried out using non-sympatric potential predators, the presumed ecological roles become highly speculative, because laboratory results cannot and should not be directly extrapolated to the field. The possibility that chemical compounds are used in the field against a wider range of predators than those usually tested in the laboratory remains to be proven in most cases [1,2]. In general, as reported below, very few studies have been conducted in the field against sympatric predators, and, thus, the ecological role of NPs in the field should be carefully considered.

#### 2.1.1. Nudibranchia

##### Doridacea

This is the most studied group of heterobranchs regarding compounds against predation (Figure 1, Figure 2 and Figure 3). Even the most basal species are protected against potential predators, such as the Antarctic *Bathydoris hodgsoni* [53,54]. This large slug presents the drimane sesquiterpene hodgsonal (**1**), which is located in its mantle and dorsal papillae, and which is suggested to be de novo biosynthesized. Hodgsonal (**1**) was the first described 2-substituted drimane sesquiterpene from a marine organism [55,56]. While *B. hodgsoni* is chemically protected against sympatric predators, such as the sea star *Odontaster validus* and the anemone *Epiactis* sp., its egg masses seem to rely only on physical defenses [54,57]. The related Antarctic species, *Prodoris (Bathydoris) clavigera* also possesses chemical defenses against *O. validus*, but the compounds behind this activity have not been yet described (C Avila and K Iken, unpublished results; [2]).

The most studied group within Doridacea are the Doridoidei, comprising the well-known dorids, phyllids, and chromodorids, among others. The Antarctic *Doris (Austrodoris) kerguelenensis* possesses a series of diterpene diacylglycerides (**2**) along with monoacylglycerides, and monoacylglycerides of regular fatty acids, which are located in the mantle and deter sympatric predators, such as sea stars (*O. validus*) and anemones (*Epiactis* sp.) [1,58,59,60,61]. This slug possesses many other molecules that may not be involved in defense against predators, including additional diterpene glycerides with different skeletons, such as *ent*-labdane, labdane, halimane, clerodane, and isocopalane diterpenes, as well as norsesquiterpenes [18,58,59,62,63,64,65,66,67]. Cryptic speciation has been reported in *D. kerguelenensis,* and this could be behind their chemical variability, even at the intrapopulation level, as well as perhaps the presence of different terpene synthase variants involved in their de novo biosynthesis [61,67,68,69,70]. Since these compounds occur in complex mixtures in the slug, it seems difficult to trace the bioactivity to the individual compounds. *Doris* (*Archidoris)* species also present similar glycerid compounds [1,71].

Several species have been reported to use steroids against potential predators. This is the case of *Aldisa sanguinea*, and perhaps also the Brazilian *Doris* aff. *verrucosa* [1,72]. The steroidal acids, 3-oxo-chol-4-ene-24-oic acid (**3**) and its unsaturated analogue (**4**) were reported from *Aldisa sanguinea* (*A. cooperi*), probably originated from some related inactive compounds from its diet of the sponge *Anthoarcuata graceae* [73]. The 3-oxo-chol-4-ene-24-oic acid (**3**) deterred feeding in the common freshwater goldfish (*Carassius auratus*) in laboratory assays [73]. Similarly, a progesterone homologue was found in the mantle of *Aldisa smaragdina* from Spain [74]. Another species*, A. andersoni* from India, is protected against predators by two phorboxazoles, 9-chloro-phorbazole D (**5**) and *N*1-methyl-phorbazole A (**6**), and the phorbazoles A (**7**), B (**8**), and D (**9**) located in their mantle and viscera [55,75,76]. The phorbazoles are chlorinated phenyl-pyrrolyloxazoles that were previously found in the sponge *Phorbas* aff. *clathrata*, and, therefore, a dietary origin from a sponge has been suggested [55,56,75,76]. The two phorboxazoles (**5,6**) and phorbazole A (**7**) were tested in the laboratory at 1 mg/mL against the shrimp *Palaemon elegans* and showed to be deterrent, although they were not in their natural concentration [75,77].

The Pacific slug *Sclerodoris tanya* presents the sesquiterpene glyceride esters tanyolides A (**10**) and B (**11**) in its mantle, reported to be effective deterrents against sympatric fish predators, such as *Gibbonsia elegans* and *Paraclinus integrippinis* at 1 mg/pellet [78]. The Mediterranean *Paradoris (Discodoris) indecora* incorporates furanosesterterpenes, including variabilin (**12**), from its sponge preys *Ircinia variabilis* and *I. fasciculata* [79] as deterrents against fish predation [79]. Variabilin (**12**) was tested in the laboratory at 300 μg/cm^2^ against freshwater and marine fishes [79].

*Dendrodoris* species are well studied, with polygodial (**13**) from *D. limbata* being the first example of de novo biosynthesis in nudibranchs [80,81]. Polygodial (**13**), a drimane sesquiterpene, was first described in plants, where it is a deterrent against herbivores [82], and it is a deterrent in the slug against predation by marine and freshwater fish [80]. Polygodial (**13**) was found to be transformed from olepupuane (**14**) once secreted from the mantle cells, since it is not present in vivo in the slug tissues [80,83,84]. Furthermore, some fatty acid-esterified sesquiterpenoids were also found in *D. limbata*, and later in other species, generally found in the reproductive organs and egg masses and possibly with other functions, or perhaps just being stored as putative precursors of polygodial (**13**) [85]. Further studies with many other *Dendrodoris* species around the planet have yielded similar drimane sesquiterpenes located in the mantle, such as in *D. arborescens*, *D. carbunculosa*, *D. denisoni, D. grandiflora*, *D. carbunculosa*, *D. krebsii, D. nigra*, and *D. tuberculosa*, which are suggested to be used as feeding deterrents against predators [1,2,81,86,87,88,89,90,91,92,93,94]. In particular, *D. arborescens* presents 7-deacetoxyolepupuane (**15**) [87], *D. carbunculosa* possesses dendrocarbins A–N (**16**) [86], *D. krebsi* also has drimane sesquiterpenes and esters [89,90], and *D. denisoni* has cinnamolide (**17**), olepupuane (**14**), and polygodial (**13**) in its mantle [88].

*Doriopsilla* species also present similar metabolites to the related genus *Dendrodoris*. The Atlantic *Doriopsilla pelseneeri* presents the furanosesquiterpene alcohols pelseneeriols-1 and -2 (**18**) in the mantle [81,85,95,96,97]. *D. albopunctata* and *D. areolata* also have drimane sesquiterpenes and *ent*-pallescensin A (**19**) [89]. Other *Doriopsilla* species studied possess also drimane sesquiterpenoids and sesquiterpenoids with the *ent*-pallescensin A (**19**) skeleton in the mantle, including *D. janaina* and *D. pharpa* [81,89,95,96,97,98]. These natural products are de novo biosynthesized by the slugs, such as 15-acetoxy-*ent*-pallescensin (**20**) via the mevalonic pathway in *D. areolata* and *Doriopsilla* sp. [81,96,97,99]. It has been suggested that these compounds are used for defense against predators, but very few assays have been reported [81,96]. These include only the extracts of *D. pharpa* presenting polygodial (**13**), which deter feeding of the blenny fish *Chasmodes bosquianus* and the mummichog fish *Fundulus heteroclitus*, which even learned to avoid food items with extracts of slugs, and also deter the crabs *Callinectes similus* and *Panopeus herbstii* in the field [98].

The group of phyllidids has also been well studied over the last years [1,4]. These are usually brightly colored tropical animals, very specious, and quite similar in their external morphology, which has often resulted in some misidentifications [2,4,100]. These slugs are characterized by presenting isocyanate compounds that display a wide array of activities, apart from avoiding predation (see below) [1,101,102,103,104,105]. The first species studied was *Phyllidia varicosa* from Hawai’i, where a toxic compound, 9-*iso*cyanopupukeanane (**21**), and a tricyclic sesquiterpene isocyanide were described almost 50 years ago [106]. The compound was also found in its prey, the sponge *Ciocalypta (Hymeniacidon)* sp. [106], and a related compound was subsequently reported in the slug, 2-*iso*cyanopupukeanane (**22**) [107]. The extracts of Palauan *P. varicosa* deterred feeding by sympatric reef fish at natural concentration [108]. Similarly, the extracts from other species from Guam of the related genus *Phyllidia*, *Phyllidiella*, *Phyllidiopsis*, and *Fryeria* are deterrent to the sympatric crabs *Leptodius* sp., the mantle extracts being more deterrent than the viscera extracts [2]. A fast transformation of the secreted compounds was reported and was related to the loss of the deterrent activity [2]. The analysis of the sesquiterpene isocyanides that these slugs present suggests a broad diet of different demosponges, indicating a wide feeding variability [22]. Some experiments with agar-based food combined with different color patterns were also conducted, and the results showed that phyllidiids were defended against fish predators [109]. *P. varicosa* also possesses two 9-thiocyanatopupukeanane sesquiterpenes found in epimeric mixture; these were traced to its prey, the demosponge *Axinyssa aculeata* [110]. One of them is located in the mantle and is probably related to defense, but both compounds are found in the viscera, indicating their dietary origin. *Phyllidia coelestis* from Thailand also contains two pupukeanane sesquiterpenoids suggested to be used as for defense against predators [2,109,111]. *Phyllidia elegans* from Guam was a deterrent against reef fish, although the natural products have not been yet identified [109]. Other *Phyllidia* species contain related compounds, such as *Phyllidia picta* from Bali yielding two axane sesquiterpenoids, pictaisonitrile-1 (**23**) and pictaisonitrile-2, and *Phyllidia* sp. From Sri Lanka presenting the sponge-related 3-*iso*cyano-theonellin (similar to a cyanide from *Axinyssa*), together with some nitrogenous bisabolene sesquiterpenes [112,113,114,115].

*Phyllidia varicosa*, *P. ocellata*, *Phyllidiella pustulosa,* and *Phillidiopsis krempfi* from Australia also present three more sesquiterpene isonitriles, 10-*epi*-axisonitrile-3, 10-*iso*cyano-4-cadinene, and 2-*iso*cyanotrachyopsane, and the peroxide 1,7-*epi*dioxy-5-cadinene, together with some more sesquiterpene isonitriles [102,116]. Moreover, *Phyllidia ocellata* and *Phyllidiella pustulosa* contain stereoisomers of 10-*iso*cyano-4-amorphene and of 4-*iso*cyano-9-amorphene, respectively [102,116]. *Phyllidia coelestis* and *Phyllidiella pustulosa* from South China and their potential prey *Acanthella cavernosa* contain a nitrogenous cadinane-type sesquiterpenoid, xidaoisocyanate A (**24**), together with other sesquiterpenoids and diterpenoids [117]. *P. pustulosa* from Fiji possesses axisonitrile-3 (**25**), an isothiocyanate, and some minor related sesquiterpenes [118]. In China and Vietnam, *P. pustulosa* also presents sesquiterpene isocyanides, isothiocyanate, as well as some sterols, some of them also reported in *Acanthella* sponges, while in Japan, a sesquiterpene isonitrile is reported [103,119,120,121,122]. Samples from Hainan island present diterpenes together with sesquiterpenes, with the diterpenes amphilectene (**26**), kalihinol-A (**27**), and kalihinol-E (**28**) being previously found in sponges, and the sesquiterpene *ent*-stylotelline (**29**) being the enantiomer of the sponge compound stylotellin [120,123]. Amphilectene (**26**), kalihinol-A (**27**), and kalihinol-E (**28**) display deterrence in the laboratory against the allopatric goldfish *C. auratus* at 50 μg/cm^2^ [120]. *P. pustulosa* is therefore a chemically rich species, containing a wide variety of compounds, perhaps related to its unrestricted sponge diet, or to the presence of unknown cryptic species, but only a few of their metabolites have been tested against predation. Moreover, in field experiments, living *Phyllidiella granulatus* were offered to fish but were never consumed, while crude lipophilic extracts of three species of phyllidiids were shown to be effective against fish predation [109]. These were *Phyllidia varicosa* from Palau, *P. elegans* from Guam, and *Phyllidiella pustulosa* from Palau, where crude extracts at natural concentrations deterred feeding by sympatric reef fish, such as *Abudefduf sexfasciatus*, *A. vaigiensis*, *Cheilinus fasciatus*, *Thalassoma lutescens*, *T. hardwickii*, *Naso vlamingii*, and *Bodianus axillaris*, although *P. pustulosa* extracts from Guam did not [109]. In this study, the authors reported that visual and chemical cues are more effective against fish when used together than either of them alone [109]. 

Another exhaustively studied group is that of “chromodoridids”, which possess a huge diversity of compounds from their diet of demosponges, often accumulating them in mantle dermal formations (MDFs) [1,4,124]. This group was recently the subject of important taxonomical revisions that resulted in changes in several genus names [125]. One of the first species studied was *Cadlina luteomarginata*, where natural mixtures of three isocyanides and three isothiocyanates from its sponge prey were found, with the isocyanides (**30**) being deterrent in laboratory assays against goldfish at 10 μg/mL and both mixtures being deterrent against the woolly sculpin *Clinocottus analis* [126,127]. Some terpenoids from *C. luteomarginata* are de novo biosynthesized, while others are obtained from its sponge diet [128]. Specimens from British Columbia present de novo produced albicanyl acetate (**31**), cadlinaldehyde (**32**) and luteone (**33**) [128]. Albicanyl acetate (**31**), which is concentrated in mantle and mucus, was shown to be deterrent [129]. The related 1a,2a-diacetoxyalbicanyl acetate (**34**) was found in their egg masses and was suggested to be involved in defense against predators based on structural similarity [128,130]. 

*Chromodoris* is among the most studied heterobranch genus, although many studies were published using different names [76,131,132,133,134,135,136,137,138,139,140,141,142,143,144,145,146,147,148,149,150,151,152,153,154,155,156,157,158,159,160,161,162,163,164,165]. These slugs accumulate mostly terpenoids from their diet sponges, and many different structures have been reported, including sesquiterpenes, diterpenes and nor-diterpenes, sesterterpenes, macrolides, and bromophenols [131,132,133,135,136,137,138,139,140,141,142,143,144,145,146,147,148,149,150,151,152,153,154,155,156,157,158,159,160,161,162,163,164,165]. Previous studies analyzed the chemistry in the Mediterranean species *C. luterorosea*, *C. purpurea*, *C. krohni*, and *C. britoi* [1,2,4], containing diterpenoids from *Spongilla* sponges, while tropical species such as *C. mandapamensis* from India contain spongiadiol (**35**), previously found in sponges from Australia, within a mixture of related spongiane compounds [166]. In the Red Sea, *C. africana* presents the furanoterpene kurospongin (**36**), as well as a 14-membered macrolide with an attached 2-thiazolidinone unit, latrunculin B (**37**) [167,168,169,170]. Kurospongin (**36**) was obtained also from a *Spongia* sp. in Okinawa and reported to be deterrent [167,168,169]. Latrunculin B (**37**) was also found in *C. (Glossodoris) quadricolor* [171] and in the sponge *Latrunculia magnifica* [169,170]. In fact, also latrunculin A (**38**) is a sponge compound initially found in *L. magnifica* and reported in the MDFs of several *Chromodoris* species [136,141,153,164,169]. Other macrolides, such as laulimalide (**39**) and *iso*laulimalide (**40**), were reported in *C. lochi* and its sponge prey, *Hyattella* sp. [142,172,173,174]. *C. hamiltoni* from South Africa presents hamiltonins A–D (**41,42**), atypical chlorinated homoditerpenes, as well as the sesterterpene hamiltonin E (**42**) and latrunculins A and B (**37,38**), while specimens from Mozambique possess two spongian diterpene lactones in addition to latrunculin B (**37**) [153,155]. Many other compounds have been described in this genus, often located in the MDFs and suggesting a defensive role, but unfortunately very few tests for deterrence have been carried out [1,4].

In the genus *Glossodoris*, *G. vespa* and *G. averni* from Australia, as well as *G. pallida* from China, contain 12-deacetoxy-12-oxoscalaradial (**43**), while *G. pallida* from Guam contains some sesquiterpenes, such as scalaradial (**44**), deacetylscalaradial (**45**), and deoxoscalarin (**46**) [175,176,177]. The sesquiterpenes from *G. pallida* from Guam, located in their MDFs, have been proven to act as deterrents against sympatric reef fish (*Abudefduf sexfasciatus*, among others) and crabs (*Leptodius* sp.) at natural concentrations [176,177]. Further studies with *G. vespa* showed high concentrations of sesquiterpenes in mantle rim tissues that were more unpalatable to the allopatric palaemonid shrimp *Palaemon serenus* than metabolites from the viscera, suggesting selective accumulation of dietary compounds or perhaps even biotransformation to more potent defenses [178]. 

As taxonomical studies progress, many *Chromodoris* and *Glossodoris* species have been renamed, such as *Goniobranchus*, *Ardeadoris*, *Doriprismatica*, *Felimare,* and *Felimida*, respectively [171,175,179,180,181,182,183,184,185]. *Goniobranchus collingwoodi* presents six spongian-16-one diterpenes in the mantle, and the extract of the whole body displayed deterrence against the allopatric palaemonid shrimp *P. serenus* [185]. *G. reticulatus* from Australia contains a dialdehyde sesquiterpene and its ring-closed acetal, also reported in *G. sinensis* from China, where they are described to be deterrents against *Palaemon elegans* [186]. Specimens of *G. splendidus* from different localities in east Australia were described to present different abundances, types, and richness of natural products in addition to high individual variation between specimens from the same population [187]. These variations resulted in different potencies when deterring feeding in the allopatric, generalist rock-pool shrimp *P. serenus*, but in all cases, the specimens showed deterrent activity [187,188]. Other *Goniobranchus* species, such as *G. albonarus*, present diterpenes and nor-diterpenes obtained from their sponge prey, but they have not been tested for feeding deterrence [189,190,191,192].

Another interesting genus within this chromodorid group is *Ceratosoma*, because these species present a dorsal protuberance containing MDFs loaded with furanosesquiterpenoids. Although a defensive role has been suggested and it seems highly probable, it still remains to be demonstrated using sympatric predators [22,193]. These species include *C. trilobatum* and *C. gracillimum* from China, which possess pallescensin-B (**47**), (–)-furodysinin (**48**), (–)-dehydroherbadysidolide (**49**), and (–)-herbadysidolide (**50**) previously reported for *Dysidea* sponges [22,193,194,195,196,197]. From them, (–)-furodysinin (**48**) shows deterrent activity against the goldfish *Carassius auratus* in the laboratory [194]. Another compound, nakafuran-9 (**51**), present in *C. gracillimum* specimens from Hainan, was also reported as a deterrent [131]. In Australia, *C. trilobatum* possesses furodysinin (**48**), furodysin (**58**), and dendrolasin (**55**) in the viscera and, additionally, agassizin (**59**) and dehydroherbadysidolide (**49**) in the mantle, while *C. brevicaudatum* presents mixtures of the same compounds along with some unidentified metabolites [178].

*Hypselodoris* is another well-studied genus, although some species are now named *Felimare* or even *Risbecia* [125,131,165,198,199,200,201,202,203,204,205,206,207,208]. All of these species possess diet-derived furanosesquiterpenes, among other terpenoids, located in their MDFs [131,165,198,199,200,201,202,203,204,205,206,207,208,209]. Longifolin (**52**) is one of the main furanosesquiterpenes found in these groups, is located in MDFs, and is a deterrent in the lab against the goldfish *Carassius auratus*, like several other compounds of theirs [131,198,201,208]. Many of these molecules are obtained from *Dysidea* sponge species [165,200,206,207]. Some of the studied species include the Mediterranean *F. picta webbi*, *F. villafranca*, *F. cantabrica*, *F. tricolor*, *F. fontandraui*, and others, presenting longifolin (**52**) and some related compounds [2,124]. In the laboratory, the crude extracts of *F. cantabrica* displayed stronger deterrence against the allopatric shrimp *Palaemon elegans* than extracts from their prey sponge, *Dysidea fragilis*, suggesting a selective accumulation of compounds [206]. The main chemical behind the deterrence was nakafuran-9 (**51**). The Mediterranean and North Atlantic species mentioned above have aposematic colorations and conform Müllerian mimicry groups [1,210]. *F. fontandraui*, however, does not present MDFs and presents tavacpallescensin (**53**) in its mantle rim [6,205,210,211]. Tavacpallescensin (**53**) is a deterrent against the allopatric shrimp *Palaemon elegans* at 1 mg/mL in the laboratory, a very low concentration compared to that reported in its mantle (25.98 ± 1.41 mg/mL) [205]. In the Atlantic, *F. picta webbi* presents longifolin (**52**) and tavacfuran, while *F. picta azorica* also presents microcionin-1 [212]. *Hypselodoris capensis* presents the feeding deterrents nakafuran-8 (**54**) and -9 (**51**), which are active against the reef fishes *Chaetodon* spp., together with the sesterterpene 22-deoxy-23-hydroxymethyl-variabilin and other sesquiterpenes and sesterterpenes from its presumed prey, the sponges *Fasciospongia* sp. and *Dysidea* sp. [213]. The Australian *H. obscura* contains dendrolasin (**55**), (–)-euryfuran (**56**), and (+)-pallescensin A (**57**), while *H. whitei* presents (–)-euryfuran (**56**), (–)-furodysin (**58**), (–)-furosydinin (**48**), and dendrolasin (**55**), some of which are deterrents against the shrimp *P. elegans*, as previously mentioned [186]. *H. infucata* from Hawai’i also possesses nakafuran-8 (**54**) and -9 (**51**), probably obtained from *Dysidea fragilis* [157]. In Bali, *H. infucata* presents (–)-furodysinin (**48**), and its crude extract is repellent against the sympatric shrimp *Penaeus vannamei* at natural concentration [214]. In Hawai’i, *H. infucata* (*Chromodoris maridadilus*) contains a 3:1 mixture of nakafuran-8 (**54**) and nakafuran-9 (**51**), like its sponge prey *Dysidea fragilis*, both reported to be deterrent [165]*. H. bennetti* and *H. obscura* from Australia contain euryfuran (**56**), but *H. obscura* also has furodysinin (**48**), furodysin (**58**), and dendrolasin (**55**), while *H. bennetti* presents agassizin (**59**), dehydroherbadysidolide (**49**), and pallescensone (**60**) [178]. In addition, in Australia, *H. tryoni* presents dehydroherbadysidolide (**49**), furodysinin (**48**), nakafuran-9 (**51**), and dendrolasin (**55**) [178]. In India, *H. kanga* and its prey sponge *Dysidea* sp. also present furodysinin (**48**) [166]. In Brazil, *H. lajensis* presents furodysinin lactone (**61**), also originated from *Dysidea* species [207]. Other *Hypselodoris* species such as *H. jacksoni* contain similar or related compounds, but no activity against potential predators has been shown [209]. Similarly, the related *Mexichromis festiva* has euryfuran (**56**) and dendrolasin (**55**), while *M. mariei* presents only euryfuran (**56**) [178]. Other chromodoridid genera like *Tyrinna* contain interesting compounds, but none of them have been demonstrated to be used against predation to date [131,179,215,216]. 

The genus *Hexabranchus* mainly contains macrolides. In several locations around the Pacific and the Indo-Pacific, *H. sanguineus* presents several macrocyclic lactones, but only kabiramides and halichondramide derivatives have been proved to be deterrents against the sympatric fish *Thalassoma lunare* and the crab *Dardanus megistos* [217,218,219,220]. Active compounds consist mainly of kabiramide C (**62**) and halichondramide derivatives, such as dihydrohalichondramide (**63**) [217,218,220,221]. These macrolides are found in the slugs and even at higher concentrations in their spawn, suggesting a defensive role [218,222]. Since these compounds are found in mantle and viscera, they are suggested to be obtained and biotransformed from their diet of *Halichondria* sponges [218,221]. *H. sanguineus* from Fiji contains also macrolides along with two thiazole cyclic peptides, sanguinamides A (**64**) and B [219]. 

Finally, within the group of nembrothids, the tambjamines (**65–71**) are alkaloids obtained from their diet of several species [223]. *Tambja abdere* and *T. eliora* in the east Pacific accumulate tambjamines (**65–71**) from the bryozoan *Sessibugula translucens,* and they are in turn preyed on by another nembrothid slug, *Roboastra tigris* [61,224,225]. In Micronesia, *Nembrotha* species present tambjamines (**65–71**) from their ascidian prey, *Atapozoa* sp. [157,226,227]. These compounds include mixtures of tambjamine A (**65**), B (**66**), C (**67**), D (**68**), E (**69**), and F (**70**); a tambjamine aldehyde (**71**); and a blue tetrapyrrol (**72**) [226]. Crude extracts and mixtures containing tambjamine C (**67**) and F (**70**) and the tetrapyrrol (**72**) are reported to be deterrents against fish at (or below) natural concentrations, while tambjamines A (**65**) and E (**69**) are not deterrents [61,226]. *R. tigris* feeds on *T. abdere* and *T. eliora,* accumulating tambjamines A–D (**65–68**) [223]. Both *Tambja* species and *R. tigris* are able to detect the tambjamines released into the mucus by chemoreception and thus chemically locate their prey [61,223]. When the concentration of tambjamines is very high, *R. tigris* may reject its prey [61,223]. Similarly, tambjamines have also been reported in *T. ceutae* and *T. stegosauriformis* and their bryozoan prey, *Bugula dentata* [207,228].

##### Dendronotida

In Florida, *Tritonia hamnerorum* presents julieannafuran (**73**), a furano-germacrene obtained from its diet, the sea fan *Gorgonia ventalina* [229]. Julieannafuran (**73**) has been shown in reliable field assays to be a deterrent at natural concentrations against sympatric reef fish, such as *Thalassoma bifasciatum*, as well as in the laboratory [229]. The Antarctic *Tritonia challengeriana,* instead, has been proved to be chemically protected against feeding by the sympatric sea stars *Odontaster validus*, but no compounds have been identified from it to date ([2], Avila and K Iken, unpublished results). Furthermore, in Antarctica, *Tritoniella belli* sequesters 1-O-hexadecyl glycerol (chimyl alcohol) (**74**) from its diet, the stoloniferan coral *Clavularia frankliniana* [230,231,232]. This compound provides protection against the potential sympatric predator, the sea star *O. validus*, which is also deterred by the mantle tissue of the slug ([2,230,231,232], Avila and Iken, unpublished results). The spawn of *T. belli* is also chemically defended against predators [232,233]. 

*Tritoniopsis elegans* presents the sesquiterpenes tritoniopsins A–D (**75–78**) in the mantle, which are obtained from its diet of the soft coral *Cladiella krempfi* [234]. Tritoniopsins A (**75**) and B (**76**) are the major compounds, with tritoniopsin A (**75**) more abundant in the slug and tritoniopsin B (**76**) in the soft coral, thus suggesting a selective accumulation by the slug, which incorporates it in its mantle possibly for protection against potential predators [234]. 

The Mediterranean *Marionia blainvillea* presents homarine (**79**), a widespread zwitterionic natural product described to be a feeding deterrent, but it has not been tested against sympatric predators of the slug [235]. Homarine (**79**) has been suggested to derive from its cnidarian diet and could be the only defense of this slug that has no nematocysts [235]. Furthermore, homarine (**79**) has been found in other molluscs, for example, in Antarctica (*Marseniopsis mollis*), where it was described to deter feeding in the seastar *Odontaster validus* [235,236].

The colorful *Tethys fimbria* was described to de novo biosynthesize a series of prostaglandins (PG) and PG–lactones [1,237,238,239,240]. These compounds are well known in many organisms as promotors of hormonal responses [28]. Different PGEs, such as PGE_2_-1,15-lactone (**80**) and PGE_3_-1,15-lactone (**81**) are found in *T. fimbria* cerata [237], while PGFs are present in the reproductive system of the slugs [239]. Since cerata are detached when the animal is disturbed, together with a copious amount of mucus and a strong antero-posterior waving movement, PGEs are suggested to be involved somehow in defense, autotomy, and/or tissue protection, as well as further regeneration of cerata, while PGE–lactones (**80,81**) are converted to the free acid forms PGE_2_ and PGE_3_, respectively [237]. Similarly, *Melibe viridis* contains one of these prostaglandin lactones (**80**) in its mucus and cerata, suggested to be used for defense against predators [77].

##### Euarminida

Only one species has been suggested to use defensive compounds in this group [1,2], the Antarctic *Charcotia granulosa* [241,242], although no experiments have proved this yet. This species possesses a unique linear homosesterterpene lactone, granuloside (**82**), probably stored in its MDF-like structures [242]. Granuloside (**82**) was isolated from the lipophilic extract of the mantle of the slug, while it was absent in the gut and digestive gland as well as in the prey of the nudibranch, the bryozoan *Beania erecta*, strongly supporting its de novo biosynthetic origin. Sesterterpenes are known in nudibranchs [4], but, to date, granuloside (**82**) is the only known linear homosesterterpene in nature. 

##### Aeolidida

Homarine (**79**), previously mentioned above, has been also found in the Atlantic aeolidids *Cratena pilata* and *Cuthona gymnota,* the Pacific *Hermissenda crassicornis*, the Australian *Phestilla lugubris,* and the Mediterranean *Cuthona coerulea* [2,92,235]. It has been suggested that the slugs obtain homarine (**79**) from their diet of hydrozoans or other cnidarians [235]. *Flabellina exoptata*, *F. ischitana*, *F. pedate,* and *F. affinis* also contain homarine (**79**) [235,243]. Despite the fact that homarine (**79**) has not been tested specifically for these species, its potential deterrent role cannot be overruled (see above) and may complement their cnidocyst defenses. 

*Phyllodesmium* species do not to present functional cnidocysts, and, thus, their chemical defenses become their only protective shield, together with their cryptic behavior [244,245,246,247,248]. *P. magnum* from China presents an uncommon asteriscane sesquiterpene related to 11β-acetoxypukalide (**83**), as well as some other sesquiterpenes [249]. 11β-acetoxypukalide (**83**) was previously reported to be the chemical defense of *P. guamensis* from Guam, which accumulate it in their cerata, and it was suggested to be obtained from feeding on *Sinularia* soft corals [246]. 11β-Acetoxypukalide (**83**) was shown to deter feeding by the sympatric omnivorous pufferfish *Canthigaster solandri* at concentrations at least an order of magnitude lower than those found in their cerata (0.5% of dry mass in artificial food) [246]. Previously, trocheliophorol (**84**) was also found to be accumulated in the cerata of the Australian *P. longicirrum* and in its prey, the soft coral *Sarcophyton trocheliophorum* [245]. Four more polycyclic diterpenes and other compounds were described from *P. longicirrum*, some of them (for example, 4-oxochatancin (**85**), (2S)-*iso*sarcophytoxide (**86**), and cembranoid bisepoxide 12) being deterrent also to the pufferfish *C. solandri* [250,251]. The 4-oxochatancin (**85**) is probably obtained from a diet of *Sarcophyton* corals [28,250,251]. *P. longicirrum* also possesses many other compounds, including steroids, cembranoid diterpenes, biscembranoids, and the above-mentioned chatancin diterpenes [251]. Other *Phyllodesmium* species have been reported to contain other interesting natural products, but its role in deterring potential predators has not been proved to date [244,248]. 

#### 2.1.2. Pleurobranchoidea 

This group is well known for presenting acidic secretions that may deter putative predators [1,2]. Examples, with pHs as low as 1–2 include *Pleurobranchaea californica, Berthellina citrina*, and *Pleurobranchus strongi* from the Pacific, as well as *Berthella plumula* and *Pleurobranchus membranaceus* from the North Atlantic. In addition, *Berthella* sp. 1 from the Mediterranean and *Berthella* sp. 2 from Antarctica display pH ~1 [2]. *P. californica* and *P. membranaceus* have also been described to possess buccal acid glands [124,252]. Both *Berthella* and *Berthellina* are usually consumers of demosponges and occasionally of calcareous sponges and corals [253], and no chemical defenses have been described for them besides the acid secretions mentioned above. Similarly, the Antarctic *Bathyberthella antarctica* presents defensive acid secretions in its mantle [254,255].

#### 2.1.3. Tylodinoidea

*Tylodina* species seem to be protected from predation by using sponge compounds and crypsis. *Tylodina fungina* from the Pacific contains an ester derivative of the brominated isoxazoline alkaloid 3,5-dibromotyrosine (**87**), which is a known feeding deterrent in sponges of the genus *Aplysina* [256]. *T. perversa* from the Mediterranean possesses similar metabolites from the sponge *Aplysina aerophoba* [257]. Finally, *T. corticalis* from Australia selectively accumulates several bromotyrosine-derived alkaloids from its sponge diet, *Pseudoceratina purpurea*, which contains a larger variety of these compounds [258]. In all cases, the natural products are sequestered by the molluscs and can then be found in the mantle, mucus, reproductive organs, and egg masses [259,260]. In the case of *T. perversa,* they feed preferentially on the symbiotic tissues of sponge prey loaded with cyanobacteria [261]. Furthermore, the slugs combine chemical defense with crypsis, while their mimetic yellow color (as well as that of their egg masses) on *Aplysina* species is due to uranidine, a phenolic pigment that becomes dark by oxidation when exposed to air, and it is also derived from the sponge [262,263].

#### 2.1.4. Cephalaspidea

Species of the genus *Philine* often secrete sulfuric acid from subepithelial notal glands, and this is supposed to be a defense against predators, similarly to acid-secreting nudibranchs and pleurobranchoids [124,264]. *P. quadripartita* from the Mediterranean, Atlantic, South Africa, and Indo-Pacific is an example, possessing sulfuric and hydrochloric acid in acidic glands [265,266]. Some other cephalaspideans are able to de novo biosynthesize their own chemical defenses, such as *Bulla striata,* a generalist algal feeder found in the Atlantic and the Mediterranean [267,268]. Remarkably, cephalaspideans, such as the voracious predator *Philinopsis depicta*, are able to prey on *B. striata*, thus obtaining chemical defenses from them—in this case, the polypropionates aglajnes 1–3 (**88**), using them for their own defense, with aglajne-1 being the most deterrent [269,270,271]. Similarly, the Pacific species *P. speciosa* contains the polypropionates niuhinones A and B (**89**), as well as a pyridine derivate pulo’upone (**90**) reported to be deterrent, and although their origin is not yet known, *P. speciosa* probably also relies on other cephalaspideans [272,273]. In fact, niuhinones A and B (**89**) have also been found in the Atlantic species *B. occidentalis*, along with the acyclic polypropionate, niuhinone C (**89**) [274]. *P. speciosa* also presents other compounds, such as the depsipeptide kulolide-1, a linear tetrapeptide (see below), pupukeamide, additional peptides, and the macrolide tolytoxin-23-acetate [275,276,277]. Similarly, *Bulla gouldiana* possesses an isomer of pulo’upone (**90**) which is further found in its cephalaspidean predator, *Navanax inermis,* and suggested to be used for its own protection [278]. Moreover, *Nakamigawaia spiralis* from Guam has been reported to chemically deter sympatric reef fish, but the active compounds have not been identified to date [279].

Homarine (**79**), again, could be used against predators in this group of heterobranchs, since it has been found in the Mediterranean *Aglaja tricolorata*, probably from its diet of sea slugs, such as dendronotaceans and/or aeolidids [235].

Another interesting group is that of *Haminoea* species. In Guam, *H. cymbalum* uses a halogenated polyacetate, kumepaloxane (**91**), which it secretes when it is disturbed and which deters porcupine fish [280]. Similarly, a chemically related brominated tetrahydropyran has been found in the same species from India, as well as in *H. cyanomarginata* from the Mediterranean, strongly deterring predation by the generalist crustacean *Palaemon elegans* [77,166]. Moreover, the spawn of *H. virescens* from the Pacific has been shown to deter feeding in decapod crustaceans, although the compound(s) has not yet been identified [281].

In Guam, *Sagaminopteron* species concentrate polybrominated diphenyl ethers, probably for defense against potential predators, although this has not yet been demonstrated. *S. nigropunctatum* and *S. psychedelicum* both feed on the sponge *Dysidea granulosa* and sequester the sponge-polybrominated diphenyl ethers, concentrating them in their mantle and parapodia [282]. One of the compounds, 3,5 dibromo-2-(2′,4′-dibromo-phenoxy)phenol (**92**), is found at higher concentrations in the slug’s parapodia (8–10%) than in the sponge or the rest of tissues of the slug (2–4%), thus supporting a potential defensive role [282].

#### 2.1.5. Anaspidea

Although sea hares are among the most studied heterobranch groups and many compounds have been described, not so many studies have focused on metabolites used to avoid predation [1,4,283]. Usually, sea hares obtain natural products from their red algal food and are often able to biotransform them [284,285,286]. Surprising reports on sea hares include specimens of *Aplysia fasciata* (*A. brasiliana*) being rejected by sharks, even when hidden in fish fillets [287]. The sharks avoided all of the pieces, except for the buccal mass, presumably containing no defensive metabolites [287]. In fact, it is well known that sea hares present glandular structures containing deterring compounds, which may be secreted or stored in their external tissues. *A. juliana* is known to use opaline and ink secretions to deter crabs, while *A. californica, A. dactylomela*, and *A. parvula* present aplysioviolin (**93**) and phycoerythrobilin, biotransformed from their algal food in the ink gland and used to avoid blue crabs’ predation [288,289,290]. Enzymatic interactions between opaline and ink secretions in *A. californica* involving escapin result in hydrogen peroxide production, and this induces deterrence against crabs, spiny lobsters, fishes, and anemones, as widely described in the literature [2,291,292,293,294,295,296,297]. Significant deterrence was also described when *A. californica* was fed on *Ulva* (green algae) and on *Plocamium* (red algae) and given to kelp bass (*Paralabrax clathratus*), and the effect proved to be stronger when the sea hare had fed on *Plocamium* (richer in natural products) [298]. *A. parvula* from Guam accumulates apakaochtodenes A (**94**) and B, two halogenated monoterpenes, from their red algal food *Portieria hornemanii*, using them as repellents against potential sympatric reef fish predators at natural concentrations [299]. In New Zealand, the same species contains several brominated and chlorinated terpenoids from the red algae *Plocamium costatum*, among which costatone (**95**) is found 14 times more concentrated in the slug than in the algae, supporting a potential defensive role [88,300].

*Stylocheilus* feeds on cyanobacteria using compounds from their diet to deter predators [301]. In Hawai’i, *S. longicauda* presents aplysiatoxin (**96**), debromoaplysiatoxin (**97**), stylocheilamide (**98**) and some complex proline esters (makalika ester (**99**) and makalikone ester (**100**)) together with lyngbyatoxin A acetate (**101**) [302,303,304,305]. Stylocheilamide (**98**) was later considered to be identical to acetyl malyngamide I, previously described from the Hawaiian cyanobacteria *Lyngbya majuscula* [306]. Moreover, the alkaloids malyngamides O (**102**) and P (**103**) were also found in the sea hare, being also structurally related to *L. majuscula* compounds [307]. Malyngamides A (**104**) and B were first found in *Microcoleus lyngbyaceus* (probably *L. majuscula*) [308]. In Guam, *S. longicauda* contains malyngamydes from the cyanobacteria and biotransforms malyngamyde B into an acetate. It has been proved that *S. longicauda* compounds are deterrents against sympatric fish (such as the pufferfish *Canthigaster solandri*), amphipods, crabs (*Leptodius* spp.), and even the herbivorous cephalaspidean *Diniatys dentifer* [309,310].

*Bursatella leachii plei* from Puerto Rico presents bursatellin (**105**), a diol nitrile alkaloid, structurally related to chloramphenicol, while *B. leachii* from the Mediterranean possesses the (+) and (–) isomers of bursatellin (**105**), in their external extracts, but no deterrent activity has been reported to date [311,312].

#### 2.1.6. Pteropoda

The amazing case of the Antarctic pelagic slug *Clione limacina* is worth mentioning here. *C. limacina* possesses a polypropionate-derived compound, pteroenone (**106**), which is a strong feeding deterrent against fish predators, such as *Pagothenia borchgrevincki* and *Pseudotrematomas bernachii* [313]. Pteroenone seems to be de novo biosynthesized, since it is not found in the prey of *C. limacina*, the thecosomate *Limacina helicina* [314]. The pelagic hyperiid crustacean *Hyperiella dilatata* captures and carries the chemically protected pteropods on its dorsum, thus increasing its chances of survival [315].

#### 2.1.7. Sacoglossa 

Despite the fact that the variety of compounds described in sacoglossa is huge [2], very few studies have tested deterrence at natural concentrations and against sympatric predators. The shelled sacoglossa *Ascobulla ulla* presents ascobullin A (**107**) and B, structurally related to oxytoxins (see below), but with less reactive molecules [316]. *Elysia crispata* from Venezuela contains, among other compounds, crispatenine and onchidal (**108**), the latter also found in the pulmonate *Onchidella* (see below) where it is presumably used to deter potential predators in its active form, ancistrodial (**109**) [316,317,318,319]. *Elysia translucens* contains udoteal as a main component from the green algae *Udotea petiolata*, which induces significant avoidance in the fish *Pomacentrus coeruleus* at 800 ppm [320].

Among the shell-less sacoglossans, the Mediterranean *Thuridilla hopei* contains the diterpenoids thuridillins (**110**), possessing a central α,β-epoxy-δ-lactone ring which is substituted by an uncyclized or cyclized isoprenoid chain and a 2,5-diacetoxy-2,5-dihydrofuran unit [321,322]. *T. hopei* also possesses *nor*-thuridillonal (**111**), the epoxylactone from the algae *Pseudochlorodesmis furcellata* [323], considered the putative precursor of thuridillins (**110**), and which is active in laboratory feeding deterrence tests against the shrimp *Palaemon elegans* at a concentration of 5.0 mg/mL [322]. *Thuridilla splendens* from Australia also presents thuridillins (**110**), but contrastingly, these thuridillins did not deter feeding by the sympatric shrimp *Palaemon serenus* in the laboratory [186,324].

The Caribbean *Costasiella ocellifera* (*C. lilianae*) contains avrainvilleol (**112**), a brominated diphenylmethane dietary algal derivative, from feeding on the algae *Avrainvillea longicaulis* [316,325]. Avrainvilleol (**112**) possesses deterrent properties against the tropical damselfish *Pomatocentrus coeruleus* at 100 ppm [316,325].

The Mediterranean *Cyerce cristallina* presents cyercene polypropionates (**113**) [326]. This slug has unknown feeding habits and may autotomize its cerata [326,327]. Cyercenes (**113**) are also found in the Australian *C. nigricans*, which feeds on *Chlorodesmis* algae and presents the algal diterpenoid chlorodesmin (**114**) [328]. The Atlantic *Mourgona germaineae* secrets a toxic mucus when disturbed and may also autotomize the cerata [329]. *M. germaineae* retains active chloroplasts form its algal diet, the calcareous green alga *Cymopolia barbata,* from which it also accumulates prenylated bromohydroquinones, such as cyclocymopol (**115**) [330]. Cyclocymopol (**115**) is similar to the deterrent avrainvilleol (**112**) mentioned above [325]. *Caliphylla mediterranea*, instead, seems to rely only on a defensive cryptic behavior to avoid predators, lacking propionates or other defensive chemistry [331]. This species captures chloroplasts from the algae *Bryopsis plumula* for camouflage and does not autotomize [331]. Contrastingly, *Placida dendritica* possesses polypropionate γ-pyrones such as *iso*-placidene A (**116**) that are probably used for deterrence; this species also uses crypsis as a defensive mechanism but does not autotomize [332]. 

#### 2.1.8. Pulmonata

While many different compounds have been described in pulmonates, very few have been appropriately tested using natural concentrations and against sympatric predators [2]. *Trimusculus costatus* from South Africa presents the diterpenoid labdanes 6β,7a-diacetoxylab-8,13-dien-15-ol (**117**) and 2α,6β,7a-triacetoxylabda-8,13-dien-15-ol (**118**), which produce feeding deterrence against the predatory fish *Pomadasys commersonnii* [333]. *T. reticulatus* from New Zealand, instead, possesses some deterrent diterpenes, such as 6β-*iso*valeroxylabda-8,13-dien-7α,15-diol and 2α,7α-diacetoxy-6/3-*iso*valeroxylabda-8,13-dien-15-ol, which are located in the mantle and foot are effective against sea star predators [334]. Other species of this genus also display antifeeding activities, such as *T. costatus* from Chile and *T. peruvianus* from South Africa [333,335,336,337].

Contrastingly, species of the genus *Siphonaria* present two different classes of polypropionates, some of which are found in the mucus and mantle border, thus indicating some sort of deterrent role, and are considered to be de novo biosynthesized [338,339]. The first type of polypropionates is represented by acyclic compounds with a 2-pyrone and furanone rings, such as siphonarienolone (**119**), structurally related to the polypropionates of the cephalaspideans (see above). This type of polypropionate is found in some species from Australia, Atlantic Ocean, and South Africa [340,341,342,343,344,345,346]. The second type possesses variable lengths in the alkyl chain, producing a polyoxygenated network that often cyclizes, for example siphonarin A (**120**), similar to polypropionates from actinomycetes, and found in *Siphonaria* from Australia, New Zealand, Pacific Ocean, and South Africa [347,348,349,350,351,352]. The species that have been analyzed to date include *S. capensis*, *S. concinna*, *S. cristatus*, and *S. serrata*, and some of their polypropionates are deterrents against fish [353]. 

The Onchidiidae possess repugnatorial glands which may contain sesquiterpenoids, depsipeptide acetates, or propionates. *Onchidella binneyi* presents onchidal (**108**), which is secreted as ancistrodial (**109**), its active form, to deter potential predators [319]. Many species of *Onchidella* present variable amounts of natural products at different geographical locations, all of them being deterrent for sea stars, such as the sympatric *Leptasterias hexactis* for *Onchidella borealis* [354,355]. *Peronia peronii* and several *Onchidium* species present polypropionates similar to those of *Siphonaria* mentioned above [356,357], as well as some depsipeptides, such as onchidin (**121**) [358,359]. Finally, *Onchidium* sp. From China presents onchidione (**122**) in the mucus and mantle [360], with a potential defensive role, as well as onchidiol and 4-*epi*-onchidiol (see below) [361,362]. 

### 2.2. Toxicity

**Figure 6:**
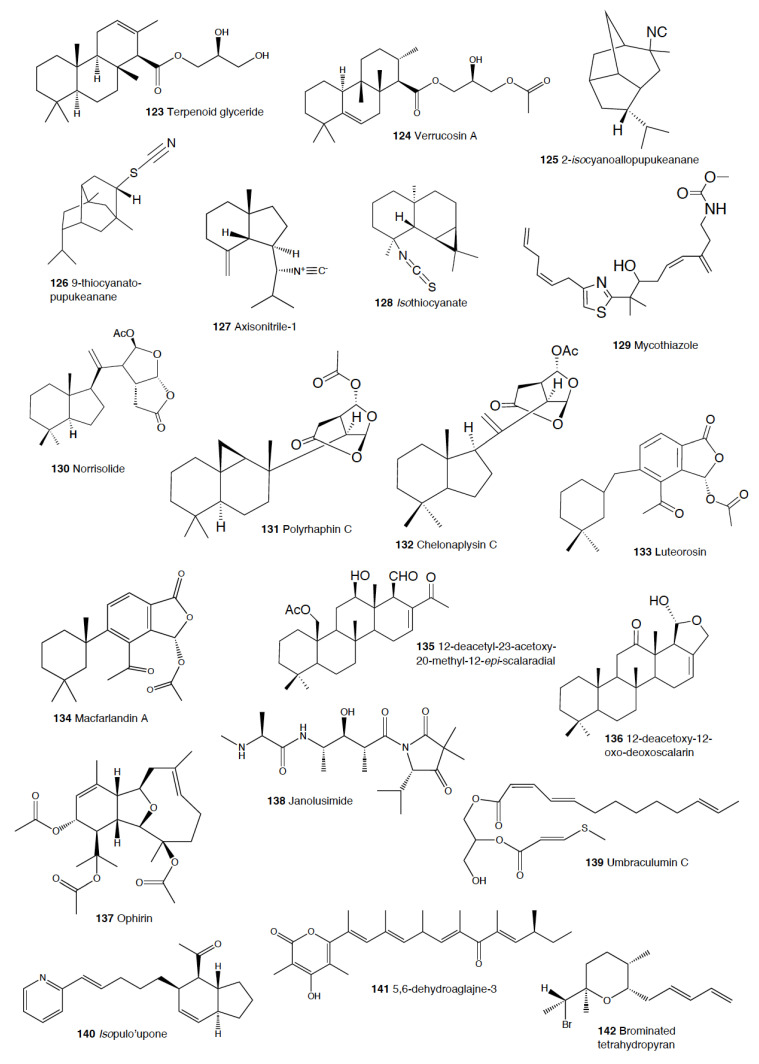
Structures of selected compounds displaying toxicity in Doridacea, Dendronotida, Euarminida, Tylodinoidea, and Cephalaspidea. These molecules may also display other activities, as reported in the text.

**Figure 7:**
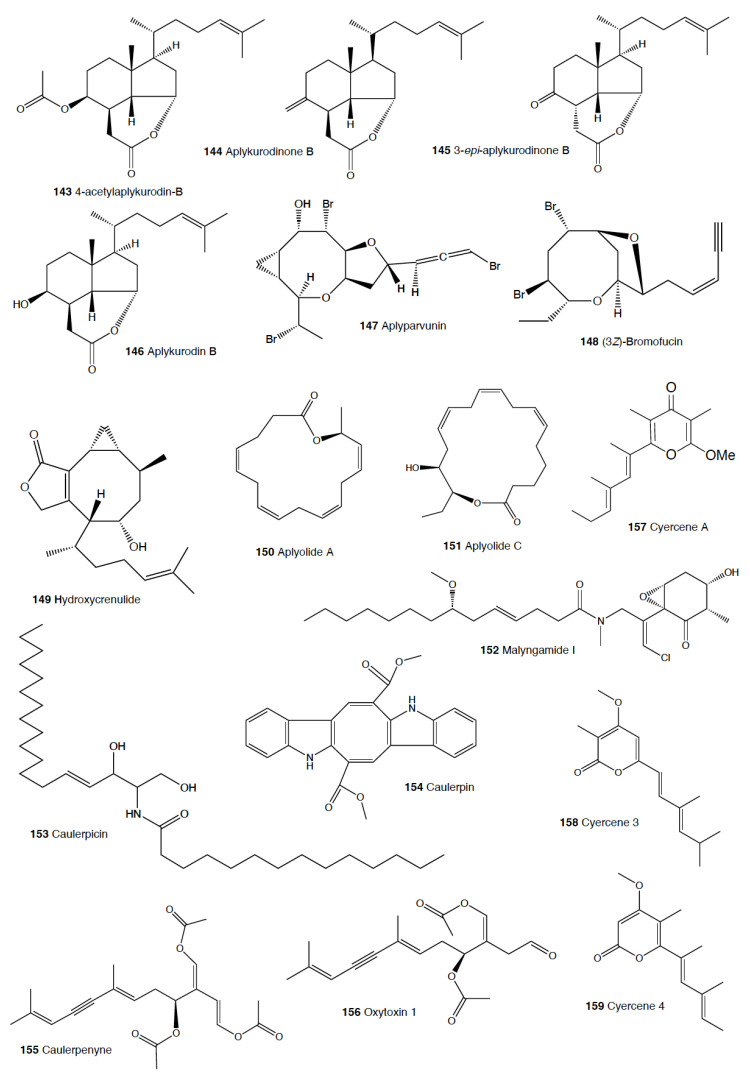
Structures of selected compounds displaying toxicity in Anaspidea, Sacoglossa, and Pulmonata. These molecules may also display other activities, as reported in the text.

Toxicity was the first described activity in heterobranch molluscs, when the mucus secretion of *Phyllidia varicosa* was reported to be toxic to fish and crustaceans [106,109]. All nudibranchs except aeolidids, and all the other groups except pleurobranchoideans and pteropods, have been described to use toxic compounds for protection and survival (Figure 6 and Figure 7). Toxicity may affect putative macropredators, such as fish, crabs, or others; small micropredators, such as amphipods or other crustaceans; and even gametes and early embryos of potential competitors or predators (Table 3). As mentioned above, the problem of assays that use species that are not sympatric puts in question the ecological validity of some of the results.

#### 2.2.1. Nudibranchia

##### Doridacea

Species of the genus *Archidoris* present de novo biosynthesized ichthyotoxic diterpene glycerides (**123**) [71,363,364,365,366,367]. In the Atlantic, *A. pseudoargus* locates them in the mantle and egg masses [363]. Their compounds include a wide variety of terpenoids and related compounds (sesquiterpenoic and diterpenoic acid glycerides and glyceryl ether), although not all of them have been tested for ichthyotoxicity [367,368,369,370,371,372,373]. *Doris verrucosa* also presents ichthyotoxic diterpenoid acid glycerides, the verrucosins (**124**), active in the laboratory against *Gambusia affinis*, and most probably biosynthesized [370,371,372].

*Phyllidia varicosa* accumulates sponge compounds and secretes them in the mucus, producing toxicity in fish and crustaceans [106,109]. Among several other bioactive compounds, 9-*iso*cyanopupukeanane (**21**) and 2-*iso*cyanopupukeanane (**22**) are obtained from the demosponge *Ciocalypta* (*Hymeniacidon)* [106,107]. When 9-*iso*cyanopupukanane (**21**) was tested using the killifish *Oryzias latipes*, it was more toxic than its 9-epi-isomer, while 2-*iso*cyanoallopupukeanane (**125**) was toxic at 10 μg/mL [101,185]. In Indonesia, *P. varicosa* feeds on *Axinyssa aculeata* sequestering two epimeric 9-thiocyanatopupekeanane sesquiterpenes (**126**), which, together with 9-*iso*cyanopupukeanane (**21**), are mildly toxic to brine shrimp (LC_50_ 5 ppm) in the laboratory [110]. *P. pulitzeri* and its sponge food, *Axinella cannabina*, possess axisonitrile-1 (**127**), which was toxic against the marine fish *Chromis chromis* and the freshwater fish *Carassius carassius* [184]. Many other phyllidid species (*P. rosans (P. bourguini)*, *P. coelestis*, *P. ocellata*, *Phyllidia* sp, *Phyllidiella pustulosa*, *Phyllidiopsis krempfi*, etc.) contain a wide variety of these and other nitrogenated compounds, but these have not been tested for toxicity [101,102,103,111,114,118,119,120,122,373,374,375,376,377].

Within chromodoridids, *Cadlina luteomarginata* presents three isocyanides (**30**) and three isothiocyanates (**128**) obtained from its sponge diet [126,127]. These metabolites resulted toxic in laboratory at 100 μg/mL, but no studies at natural concentrations and sympatric species are reported [126,127]. Further, as previously mentioned, the well-studied genus *Chromodoris* possess toxic compounds [1,6]. Kurospongin (**36**), a furanoterpene found in *C. africana* from the Red Sea, was obtained from an Okinawan *Spongia* sp. and reported to be strongly ichthyotoxic to the freshwater goldfish (*C. auratus*) at 5 μg/mL [167]. *C. hamiltoni* from South Africa and Mozambique presents one or both latrunculins A and B (**38,37**), among other compounds, as does *C. africana* from the Red Sea, and *C. quadricolor* (*Glossodoris quadricolor*) [153,168,169,170,171]. Latrunculin B (**37**) has been reported to be ichthyotoxic and was described from the sponge *Latrunculia* magnifica [168,169]. The Mediterranean *Felimida (Chromodoris) luteorosea* contains many ichthyotoxic sponge-derived diterpenes tested in the laboratory, including norrisolide (**130**), polyrhaphin C (**131**), chelonaplysin C (**132**), luteorosin (**133**), macfarlandin A (**134**), and closely related compounds [149]. Although many other *Chromodoris* species possess interesting chemicals, they have not been tested for toxicity.

Among the scalarane sesterterpenes described in *Doriprismatica (Glossodoris) sedna* from Costa Rica, 12-deacetyl-23-acetoxy-20-methyl-12-*epi*-scalaradial (**135**) was ichthyotoxic to the allopatric fish *Gambusia affinis* at 0.1 ppm [183]. *Goniobranchus splendidus* from Australia contains many sponge compounds, mainly spongian diterpenes, rearranged diterpenes, and nor-diterpenes [187]. Its chemical extracts have been proven to be toxic to brine shrimp (*Artemia* sp.) at natural concentrations, with potency depending on the mixture of chemicals present in each population analyzed, from no activity to toxicity [187]. *Doriprismatica (Glossodoris) atromarginata* presents furanoditerpenoids and scalarane sesterterpenes from its dietary sponges *Spongia* (*Hyatella*) sp. and *Hyrtios* spp., and these compounds display ichthyotoxicity against the mosquito fish, *G. affinis*—particularly, the activity of 12-deacetoxy-12-oxodeoxoscalarin (**136**) is noticeable [92,175,180,378,379,380,381,382,383,384,385,386]. Other NPs from chromodoridids were analyzed for ichthyotoxicity against *G. affinis*, including homoscalarane and scalarane compounds from *Felimida (Glossodoris) dalli*, *Glossodoris rufomarginata*, *Glossodoris pallida*, *Glossodoris vespa*, and *Ardeadoris (Glossodoris) averni*, and 12-deacetyl-23-acetoxy-20-methyl-12-*epi-*scalaradial (**135**) was the most potent of them [175,183,383].

*Ceratosoma trilobatum* and *C. gracillimum* from China contain the furanosesquiterpenes pallescensin B (**47**), (–)-furodysinin (**48**), (–)-dehydroherbadysidolide (**49**), and (–)-herbadysidolide (**50**), previously found in *Dysidea* sponges. These were tested for toxicity in the laboratory against mosquito fish and were all observed to be non-toxic except (–)-furodysinin (**48**) [22,131,193].

##### Dendronotida

The Mediterranean species *Tethys fimbria* contains a variety of de novo synthesized prostaglandins with diverse functions [1,240], among which is a prostaglandin lactone, PGE_2_-1,15-lactone (**80**), later also found in *Melibe viridis* [77]. This prostaglandin lactone (**80**) is located in the mucus and cerata of *T. fimbria* and is ichthyotoxic in the laboratory against the mosquito fish [77].

##### Euarminida

Two euarminid species are reported to present toxic compounds. In China, *Dermatobranchus ornatus* has been reported to possess compounds inhibiting cell division in fertilized starfish eggs [9]. *D. ornatus* possesses four diterpenoids of the eunicellin class in the mantle, ophirin (**137**), calicophirin B, 13-deacetoxyl calicophirin B, and 13-deacetoxyl-3-deacetyl calicophirin B, two of them probably from its diet on the gorgonian *Muricella sinensis*, and another one previously found in an unidentified soft coral from the Pacific Ocean [22,387]. Among them, ophirin (**137**) is reported to induce brine shrimp (*Artemia* sp.) lethality. The second case is that of *Janolus cristatus*, which possesses janolusimide (**138**), a toxic tripeptide which is toxic to mice at LD 5 mg/kg [388,389]. The N-methyl analogue, janolusimide B, has been further isolated from *Bugula flabellata*, a bryozoan from New Zealand, thus suggesting a putative dietary origin for janolusimide (**138**) [390].

#### 2.2.2. Tylodinoidea

The Mediterranean *Umbraculum mediterraneum* contains diacylglycerid fatty acid esters that are ichthyotoxic to the mosquito fish in the laboratory [391,392,393]. These natural products, umbraculumins A, B, and C (**139**), are suggested to be obtained from their sponge prey [263].

#### 2.2.3. Cephalaspidea

Several compounds from *Bulla* species, such as niuhinone-B, isopulo’upone (**140**), and 5,6-dehydroaglajne-3 (**141**), are polypronionates described to be toxic to fish and shrimp [274,278]. Niuhinone-B is found in the Pacific *B. gouldiana* and the Mexican *B. occidentalis* [274,278]. In the Pacific Ocean, *Navanax inermis* also uses these compounds after ingesting *B. gouldiana* specimens, while in Hawai’i, *Philinopsis depicta* probably obtains niuhinone-B from other cephalaspideans [272,273,278]. *N. inermis* also contains *iso*pulo’upone (**140**), which is reported to be a strong ichthyotoxin that significantly affects the mosquito fish *Gambusia affinis* at 10 ppm and *Artemia salina* at 2 ppm in the laboratory [271,394]. The Mediterranean *P. depicta* contains aglajne-3 (**88**), a polypropionate toxic to *Artemia salina* (LD_50_ < 35 ppm) and *Gambusia affinis* [270].

*Haminoea* species also possess some toxic compounds. In the Mediterranean, *H. cyanomarginata* presents a brominated tetrahydropyran (**142**) reported to be highly toxic to the mosquito fish *G. affinis* at 1 ppm in the laboratory [77]. This tetrahydropyran (**142**) was also found in the Indian *H. cymbalum*, where it could play the same role, and it is structurally similar to kumepaloxane (**91**) from conspecifics of Guam [280].

#### 2.2.4. Anaspidea

Several sea hares are reported to use toxic compounds. In the Mediterranean, *Aplysia fasciata* presents different compounds in different locations, with polyhalogenated monoterpenes similar to those of *Plocamium* red algae in some places [395], but some degraded sterols in other localities, such as 4-acetylaplykurodin-B (**143**), aplykurodinone B (**144**), and 3-*epi*-aplykurodinone B (**145**), which are located in the mantle and are described to be ichthyotoxic to the mosquito fish *G. affinis* in the laboratory [396]. These compounds are also related to the steroids found in the Atlantic *A. fasciata* [397] and to aplykurodin B (**146**) from the Pacific *A. kurodai* [398]. In Japan, instead, *A. parvula* possesses the ichthyotoxic brominated acetogenin dicyclic ether, aplyparvunin (**147**), which possesses strong activity (LC_100_ 3 ppm in 24h) against *G. affinis* in the laboratory [399], while specimens from South East Africa present (3*Z*)-bromofucin (**148**), a halogenated cyclic acetogenin obtained from its red algal food, *Laurencia implicata* [400]. *A. vaccaria* from the Pacific Ocean presents also ichthyotoxic compounds, in this case, the crenulides (**149**), non-halogenated diterpenoids obtained from its brown algal food, *Dictyota crenulata,* and located in their digestive gland [401,402]. Crenulides (**149**) are toxic to the reef-dwelling fish *Eupomacentrus leucosticus* at 10 µg/mL [401,402]. *A. depilans* also possesses ichthyotoxic fatty acid lactones, the aplyolides A−E (**150,151**), which are toxic in the laboratory to the mosquito fish *G. affinis* at 10 ppm [403]. In the Caribbean, *A. argus* presents ichthyotoxic biotransformed compounds from its diet, the brown algae *Stypopodium zonale*, but it possesses the bioactive diphenyl ether 2-(2′,4′dibromophenoxy)-dibromoanisole from the green alga *Cladophora vagabunda* in the digestive gland when it feeds on it [404,405].

Several bioactive compounds have also been isolated from *Stylocheilus*, mostly related to cyanobacterial metabolites [301,302,303,304,305,306,307,308,309,310]. However, only the related acetyl malyngamide I (**152**) from the Hawaiian *Lyngbya majuscula* was found to be ichthyotoxic [306], being structurally similar to stylocheilamide (**98**), a non-toxic amide from the Hawaiian *S. longicauda* [304].

#### 2.2.5. Sacoglossa 

The first toxic species reported in this group was *Oxynoe panamensis* from California, containing caulerpicin (**153**) and caulerpin (**154**) from its green algal food, *Caulerpa sertularioides* [406]. Later, in the Mediterranean, the shelled sacoglossans *Oxynoe olivacea* and *Ascobulla* (*Cylindrobulla*) *fragilis* were described to biotransform the sesquiterpenoid caulerpenyne (**155**) from its green algal food (*Caulerpa prolifera*) into the more potent ichthyotoxic aldehydes, oxytoxin-1 (**156**) and oxytoxin-2 [316,407]. In particular, oxytoxin-1 (**156**) is toxic to the mosquito fish *G. affinis* at >10 µg/mL in the laboratory, while oxytoxin-2 is toxic at 1 µg/mL. These animals are able to transport the compounds from the digestive gland to the mantle and secrete them into toxic whitish mucus [407]. Similarly, *Lobiger serradifalci*, also feeding on *C. prolifera,* presents only oxytoxin-1 (**156**) in its parapodial lobes and defensive secretion [407,408]. In the Caribbean species *Ascobulla ulla* (eating *Caulerpa fastigiata*), *Oxynoe antillarum* (eating *Caulerpa* sp.), and *Lobiger souberveii* (eating *Caulerpa racemosa*), also caulerpenyne (**155**) is also found [316]. In fact, only caulerpenyne (**155**) is detected in *L. souberveii*, while the rest of species modify it to oxytoxins (**156**) [316]. Caulerpenyne (**155**) is also found in *Volvatella* sp. in India [409].

Some shell-less species use the same system, transforming caulerpenyne (**155**) from *Caulerpa* species into oxytoxins (**156**) [410]. The Caribbean *Elysia subornata* feeds on *Caulerpa prolifera*, while *E. patina* and *E. nisbeti* feed on *Caulerpa* sp., and they all present caulerpenyne (**155**) and oxytoxin-1 (**156**) [316]. In India, *E.* cf. *expansa* also contains caulerpenyne (**155**), along with dihydrocaulerpenyne and expansinol, some minor reduced derivatives, similar to *Ascobulla ulla* compounds (see above) [411]. In *A. ulla,* ascobullin A (**107**) and ascobullin B have replaced oxytoxins, being structurally related but less reactive compounds detoxification process [316,411]. 

Avrainvilleol (**112**) from *Costasiella ocellifera* (*C. lilianae*) from the Caribbean is toxic to sympatric reef fishes at 10 µg/mL [325].

Cyercenes (**113**) are pyrone compounds found in several shell-less sacoglossans, displaying a very strong ichthyotoxicity against the mosquito fish, *G. affinis* in the laboratory [326,327]. The Mediterranean *Cyerce cristallina* de novo biosynthesizes the *α*- and *γ*-pyrones cyercene A (**157**) and B, as well as cyercenes 1–5 (**158,159**) [326,327]. In the toxicity assays, the most active compounds were cyercene A (**157**), cyercene-3 (**158**), and cyercene-4 (**159**), all at 10 µg/mL [326,327]. Although many other compounds of interest have been described in this group [19,412,413,414,415,416,417,418,419], they have not been proven to be toxic against sympatric species.

#### 2.2.6. Pulmonata

*Trimusculus costatus* from South Africa presents the labdanes 6β,7a-diacetoxylab-8,13-dien-15-ol (**117**) and 2α,6β,7a-triacetoxylabda-8,13-dien-15-ol (**118**), both toxic to the brine shrimp *Artemia salina* in the laboratory [333]. *Siphonaria* species present two different types of polypropionates, some of them located in the mucus and mantle border and reported to be ichthyotoxic [27,350]. *Siphonaria maura* from Mexico presents Vallartanone B, which in laboratory assays was rejected when applied to krill at 100 µg/mg and offered to the fish *Thallasoma lunare* [350].

### 2.3. Antimicrobials

**Figure 8:**
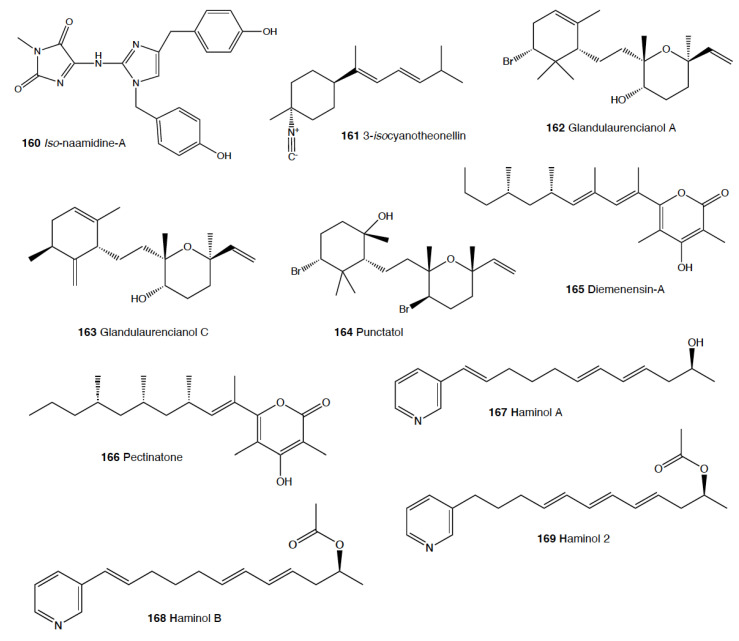
Structures of selected antimicrobial compounds in heterobranch molluscs. These molecules may also display other activities, as reported in the text.

Many marine organisms possess compounds to avoid microbial infections, and heterobranchs are no exception. Antimicrobial compounds against marine microorganisms described in heterobranchs are reported here (Figure 8, Table 4). To the best of our knowledge, however, euarminids, pleurobranchoids, tylodinoids, pteropods, and sacoglossans have not been studied for this activity to date.

#### 2.3.1. Nudibranchia

##### Doridacea

*Notodoris citrina* from the Red Sea presents several imidazole alkaloids, among which *iso*naamidine-A (**160**) has been reported to strongly inhibit the AI-2 channel of the marine pathogen *Vibrio harveyi*, acting as a quorum sensing inhibitor [424,425]. Some of the compounds of *N. citrina* have been also found in the calcareous sponge *Leucetta chagosensis,* which is the slug diet at different geographical localities [424,425]. *Iso*naamidine-A (**160**) has also been found in *Notodoris gardineri* from the Philippines [426].

Several species of the colorful Phyllidids have been reported to contain isocyanate compounds with diverse bioactive properties [1,101,102,103,104,105]. As previously mentioned, this is a particularly difficult group to study since many species and genera are similar in shape and color, resulting in many misidentifications over the years [99], although some species have been studied in depth [101,102,103,104,105,427]. *Phyllidiella pustulosa* presents compounds obtained from the sponge *Acanthella cavernosa* [119]. *Acanthella* sponges are the dietary origin for different sesquiterpene isocyanides and related compounds in specimens from China and Vietnam [119,120,121,122]. Recent chemical analysis of the South China Sea nudibranchs, *P. pustulosa* and *Phyllidia coelestis*, as well as *A. cavernosa*, reported a nitrogenous cadinane-type sesquiterpenoid, xidaoisocyanate A (**24**), among other sesqui- and di-terpenoids [117]. Moreover, axisonitrile-3 (**25**) and several minor related sesquiterpenes were isolated from the same species, *P. pustulosa,* from Fiji [118]. Moreover, *P. pustulosa* and *Phyllidia ocellata* from Australia also present some stereoisomers of 4-isocyano-9-amorphene and of 10-isocyano-4-amorphene, respectively, while *Phyllidia picta* from Bali contains the axane sesquiterpenoids pictaisonitrile-1 (**23**) and pictaisonitrile-2 [112]. *Phyllidia* sp. from Sri Lanka contains 3-*iso*cyano-theonellin (**161**), closely related to a cyanide obtained from the demosponge *Axinyssa* [113]. *P. varicosa* presents two 9-thiocyanatopupukeanane sesquiterpenes (**126**), found also in its demosponge prey *Axinyssa aculeata* [110]. Several of these compounds are reported to have an antimicrobial role. 

##### Dendronotida

Only homarine (**79**) has been described in this group as a potential antimicrobial [428], and it is found in the species *Marionia blainvillea* [235]. 

##### Aeolidida

Again, homarine (**79**) has been found in nine aeolid species as mentioned above (Table 2), and it has been reported as a potential antimicrobial, probably derived from their cnidarian food sources [235,428]. 

#### 2.3.2. Cephalaspidea

In cephalaspideans, homarine (**79**) has also been described in *Aglaja tricolorata*, originating probably from their diet of other heterobranchs [235].

#### 2.3.3. Anaspidea

Regarding sea hares, *Aplysia punctata* possesses three brominated diterpenes, glandulaurencianols A–C (**162,163**), obtained from the red algae *Laurencia glandulifera*, along with punctatol (**164**) [429,430]. All these compounds showed a laurencianol skeleton, known for its antibacterial activity and a common algal dietary source [431]. Moreover, the cosmopolitan *Aplysia juliana* presents two toxic chlorophyll derivatives, pyropheophorbides *a* and *b,* and a halogenated diterpenoid lactone, while its purple secretion also includes an antibacterial and cytotoxic peptide, julianin-S, and their egg masses are protected from microbial infections by unsaturated fatty acids [288,432,433,434].

*Dolabella auricularia* is another anaspidean known for protecting their eggs from bacterial pathogens, with a de novo biosynthesized glycoprotein, dolabellanin A, located in the albumen gland, showing antibacterial activity [435].

#### 2.3.4. Pulmonata

Some *Siphonaria* species possess polypropionates in their mucus and mantle border [27]. Compounds with a 2-pyrone and furanone rings, such as siphonarienolone (**119**), structurally related to the polypropionates of cephalaspideans, are present in several species from Australia, the West and East Atlantic, and South Africa [27,340]. Both *S. diemenensis* and *S. pectinata* display antimicrobial activity due to the presence of diemenensin-A (**165**) and pectinatone (**166**), respectively [340,341,343].

### 2.4. Antifouling

**Figure 9:**
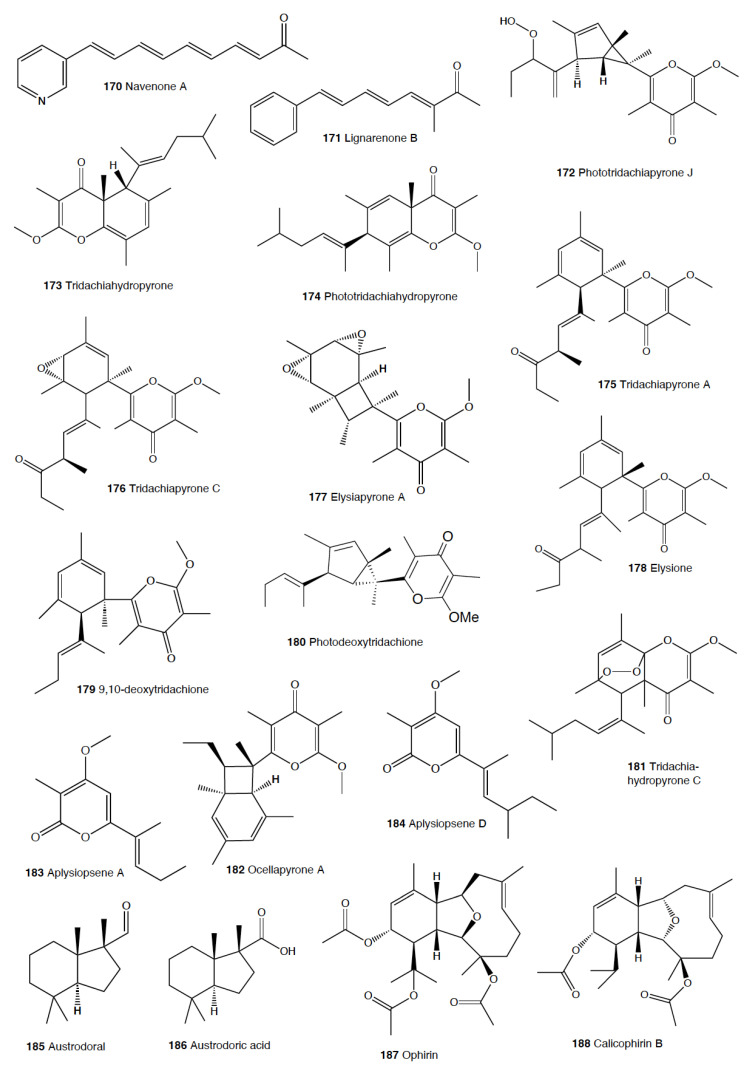
Structures of selected antifouling compounds, pheromones, tissue regeneration compounds, sunscreens, and other ecological activities in heterobranch molluscs. These molecules may also display other activities, as reported in the text.

Potentially, all surfaces under water are possible substrates for fouling colonization. Marine organisms have developed an amazing array of mechanisms to avoid fouling, and these include the use of chemicals [436]. In heterobranch molluscs, all nudibranchs except euarminids, as well as cephalaspideans, have been reported to possess antifouling compounds (Figure 9, Table 5).

#### 2.4.1. Nudibranchia

##### Doridacea

Since the isolation of 9-*iso*cyanopupukeanane (**21**) from *Phillidia varicosa* [106], phyllidids have been shown to be chemically rich, presenting many nitrogenous mono-, bi- and tri-cyclic sesquiterpenes, usually traced back to their sponge prey [1,4,22,101,102,103,104,105,106,107,110,111,114,116,118,119,120,122,374,375,376,377,437]. Some of these compounds are potent antifouling agents, effective against barnacle larvae, such as the bisabolene 3-*iso*cyanotheonellin (**161**) of *P. varicosa* from Sri Lanka, and a sesquiterpene isonitrile from the Japanese *Phyllidiella pustulosa* [102,103,114,115,116,437]. Moreover, from *Phyllidia* sp. collected at Sri Lanka, some nitrogenous bisabolene sesquiterpenes exhibited a potent in vitro antifouling activity against barnacle larvae [114,115]. Different studies on *Phyllidia ocelata*, *P. varicosa*, *Phyllidiella pustulosa*, and *Phillidiopsis krempfi* with the aim of identifying antifouling activity reported three sesquiterpene isonitriles, namely, 10-*epi*-axisonitrile-3, 10-*iso*cyano-4-cadinene, and 2-*iso*cyanotrachyopsane, as well as a peroxide, 1,7-*epi*dioxy-5-cadinene, among others [102,116]. These molecules display potent antifouling activity against larvae of the barnacle *Balanus amphitrite* (EC_50_ = 0.14 μg/mL), while axisonitrile-3 (**25**) has an EC_50_ value of 3,2 μg/mL [437]. In fact, these natural products are present in many phyllidid species, such as *P. varicosa*, *P. coelestis*, *P. ocellata*, *P. picta*, *Phyllidia* sp., *Phillidiopsis krempfi*, *Phyllidiella pustulosa,* and *P. rosans (P. bourguini)* [102,103,116,119,120,121,122,373,437]. Moreover, *Reticulidia fungia* presents sesquiterpenes such as reticulidin A (**215**) with antifouling activity [438].

##### Dendronotida

As reported above, some species present homarine (**79**), such as the Mediterranean *Marionia blainvillea*, a compound that has also been proven to display potent antifouling activity [235,428,439]. This activity was previously reported for the gorgonian *Eunicella singularis* and the soft coral *Gersemia antarctica,* and homarine (**79**) has been suggested to be incorporated in dendronotids from their octocoral cnidarian food prey [235,428,439]. The presence of homarine (**79**) in the mucus secretion of the slugs would inhibit the growth of microorganisms in the mucus [235].

##### Aeolidida

Homarine (**79**) is also found in this group, particularly in *Cratena pilata* and *Cuthona gymnota* from the Atlantic, *Hermissenda crassicornis* from the Pacific (locations mistaken in [235]), *Cuthona coerulea* from the Mediterranean, and *Phestilla lugubris* from Australia [235,440]. In this case, homarine (**79**) is originated from their hydrozan cnidarian prey and may also be used to avoid microbial colonization of the slug mucus [235,441,442]. 

#### 2.4.2. Cephalaspidea

Again, homarine (**79**) is reported here, although in a different context. The Mediterranean *Aglaja tricolorata* feeds on other heterobranchs, and may, therefore, accumulate their natural products, in this case homarine (**79**) [235,442]. 

Another compound has been reported to present antifouling activity in the mantle of *Sagaminopteron nigropunctatum* and *S. psychedelicum* [282]. This is the polybrominated diphenyl ether 3,5-dibromo-2-(2′,4′-dibromo-phenoxy)-phenol (**92**), which is also found in their prey, the demosponge *Dysidea granulosa*, together with other polybrominated diphenyl ethers [282]. This compound (**92**) is selectively accumulated in the parapodia of the slugs at a concentration of 8–10%, while it is present in other tissues of the slug and in the sponge at only 2–4% [282]. Its antifouling activity has been reported to be very high against marine bacteria, diatoms, barnacle larvae, and mussel juveniles [443].

*Haminoea cyanomarginata* from the Mediterranean is also protected against fouling by a brominated tetrahydropyran (**142**) [77]. This compound was previously found in an Australian sponge and later reported in *H. cymbalum* from India [77]. Haminols are 3-alkylpyridines also found in *Haminoea* species, which, among other activities, have been tested as antifouling molecules. These compounds include haminols A–C (**167–168**) and haminols 1–6 (**169**) [444], which are structurally related to the navenones (**170**) mentioned below. Haminol 2 (**169**) was the most potent compound, with low toxicity and strong activity against larvae of the barnacle *Amphibalanus amphitrite* [445]. De novo biosynthesis of haminols was proven in the Mediterranean *H. orbignyana* [446].

### 2.5. Trail Following and Alarm Pheromones

In nature, behavioral and chemical defenses of heterobranchs are often combined to increase survival chances [1,2]. Heterobranchs display a high number of strategies, among which alarm pheromones are used to communicate and induce behavioral changes (Figure 9, Table 6) [1,2]. Doridacean nudibranchs and cephalaspideans, and perhaps some anaspideans, display trail-following behavior, which also implies some chemoreception mechanisms.

#### 2.5.1. Nudibranchia

##### Doridacea

A trail-following behavior has been reported in some chromodoridids, such as *Hypselodoris* (*Risbecia) tryoni*, but the potential chemicals involved have not yet been described [447]. Whether this is related to defense or to reproduction remains to be further investigated.

*Tambja* and *Roboastra* are protected by the potent alkaloids tambjamines (**65–70**), which are obtained from their prey, for example, the bryozoan *Sessibugula translucens* for *Tambja abdere* and *T. eliora*, and these two slug species in turn for the voracious *Roboastra tigris* [61,226,227,228]. All these slugs are able to detect the tambjamines (**65–70**) secreted in mucous trails through chemoreception mechanisms, thus being able to find conspecifics and food (bryozoan) for *Tambja* species or detect food (slugs) for *Roboastra* [61,226]. When *Tambja abdere* specimens are disturbed, they release higher amounts of tambjamines (**65–70**), and, thus, they are also considered to be alarm pheromones [61,226]. Similar examples include *Tambja ceutae* and the bryozoan *Bugula dentata*, *Tambja stegosauriformis* and *B. dentata*, as well as some *Nembrotha* species and their ascidian prey, *Atapozoa* sp. [207,229,230,231]. 

#### 2.5.2. Cephalaspidea

Several genera in this group have been reported to secrete alarm pheromones and display trail-following behavior. Navenones A–C (**170**) were isolated long ago from *Navanax inermis* and reported to be used as alarm signals [394]. Navenones (**170**) are accumulated in a specialized ventral gland and are released within its yellow slime trail when the animals are disturbed, inducing an avoidance alarm response in the conspecifics that follow them [269,394,448]. Navenones (**170**) are suggested to be de novo biosynthesized [448]. These slugs may also be cannibalistic, but, interestingly, the secretion is not released when a small slug is attacked by a larger conspecific [318]. 

*Haminoea* species similarly release alarm pheromones that induce escape behavior in the trail-following conspecifics [271,449]. The Mediterranean species *H. exigua, H. fusari*, *H. orbignyana*, *H. orteai*, and *H. navicula* present haminols (**167–169**), oxygenated 3-alkylpyridines that are secreted in their mucous trail [19,446]. Haminols (**167–169**) are biosynthesized through sequential addition of acetic acid units to nicotinic acid [446,450]. Haminols consist of several structures similar to navenones (**170**), the haminols A–C (**167–168**) and the haminols 1–6 (**169**) [444]. Similarly, *H callidegenita* presents haminols 7–11, which are also suggested to act as alarm pheromones [271,451]. The Pacific *H. japonica* also presents a series of alkylphenols, which are structurally similar to navenone-C and are suggested to act as alarm pheromones [271,451].

The widespread *Scaphander lignarius* lives in sandy bottoms, feeding usually on foraminiferans [452,453]. This species presents the ω-arylmethylketones, lignarenones (**171**), de novo biosynthesized through a polyketide pathway from benzoic acid and accumulated in the Blochmann’s gland [454,455,456]. Lignarenones (**171**) are released within the bright yellow slime that *S. lignarius* secretes when disturbed and are also suggested to be used as alarm pheromones [454,455,456].

#### 2.5.3. Anaspidea

Mycosporine-like amino acids (MAAs) have been suggested to act as alarm cues in the sea hare *Aplysia californica* [457]. 

### 2.6. Sunscreens and UV Protection

Avoiding UV radiation (UVR) is very relevant for marine organisms that live in shallow waters, since the damaging effects of UVR on cells and tissues are potentially huge [459]. In heterobranch molluscs, those living close to the surface (plankton and pteropods) or living on algae (for food and camouflage; sea hares and sacoglossans) are the most susceptible to UVR, and UV-protecting compounds have indeed been described in anaspideans, pteropods, and sacoglossa (Figure 9, Table 7).

#### 2.6.1. Anaspidea

As mentioned above, sea hares are generalist herbivores. Among them, *Aplysia* species possess many dietary compounds from their algal food, such as many halogenated terpenoids and some carotenoids, which may provide UVR protection [459,460]. Furthermore, *A. californica* contains mycosporine-like amino acids (MAAs), the most common photoprotective molecules reported in marine organisms [457,459].

#### 2.6.2. Pteropoda

The sea butterflies *Limacina helicina* and *Clione limacina* also possess MAAs originated from phytoplanktonic microalgal species, which provide them with UV protection [461].

#### 2.6.3. Sacoglossa 

Polypropionates from sacoglossans have been reported to be used as sunscreens in several species [412,461]. These compounds are de novo biosynthesized by the slugs, and their biosynthesis is affected by UVR [462,463]. *Elysia* species possess γ-pyrone propionates, such as phototridachiapyrone J (**172**) in the Atlantic *E. patagonica* [412], and tridachiahydropyrone (**173**) and phototridachiahydropyrone (**174**) in the Caribbean *Elysia crispata* [78,317]. Phototridachiahydropyrone (**174**) seems to originate from a rearrangement mechanism during the photochemical electrocyclic formation of tridachiahydropyrone (**173**) under prolonged UVR exposition [464]. Similar γ-pyrone compounds, tridachiapyrones A–F (**175,176**), as well as elysiapyrones (**177**), along with other compounds, were found in the Pacific *E. (Tridachiella) diomedea* [413,415,416,417,418,419]. *E. viridis* feeds on the green algae *Codium vermiliara* and biosynthesizes the polypropionate (+) elysione (**178**) [463,465]. Elysione (**178**) is also found in *E. chlorotica*, which feeds on *Cladophora* algae [414]. Tridachiapyrone A (**175**) is the enantiomer of (+) elysione (**178**), and like tridachiapyrone C (**176**) and crispatene, it has been suggested that these metabolites represent the protected forms of (+) elysione (**178**) under strong light irradiation [414,466]. All of these polyene γ-pyrone compounds are localized in cell membranes, where they may act as sunscreens, thus protecting the photosynthetic apparatus of the chloroplasts [466,467]. 

*Placobranchus ocellatus* and *Placobranchus* sp. from the Pacific Ocean also present propionate-derived γ-pyrones, such as 9,10-deoxytridachione (**179**), photodeoxytridachione (**180**), tridachiahydropyrone B and C (**181**), and *iso*-9,10-deoxy-tridachione, probably also used as sunscreens [19,412,466,468,469]. In specimens from India, the elysiapyrone-related compounds ocellapyrones (**182**) were also found [468].

### 2.7. Tissue Regeneration

As in terrestrial organisms, vagile marine organisms, as well as heterobranchs, may use autotomy and tissue regeneration as a mechanism to distract predators and escape [1,2]. In fact, many nudibranchs and sacoglossa may lose part of their mantle or their cerata [1,2]. The known compounds involved in these mechanisms are reported here (Figure 9, Table 8).

#### 2.7.1. Nudibranchia

##### Doridacea

*Peltodoris atromaculata* feeds on the sponge *Petrosia ficiformis,* accumulating polyacetylenes, petroformynes, and other compounds, although the autotomy mechanism of the slug does not seem to be related with this chemistry, and, in fact, may be related to stress or senescence instead [1,210,470].

##### Dendronotida

*Tethys fimbria* presents de novo biosynthesized prostaglandin (PG) lactones (**80–81**) involved in predator distraction by the release of their cerata [237,238,239]. When *T. fimbria* is molested, cerata are detached together with a copious amount of mucus and continue to move for some time. Although PGEs and PGFs have been suggested to be involved in defense and reproduction, respectively, a role in tissue regeneration has also been suggested, since they are common hormonal compounds [237,238,239,240,471,472]. In fact, PGE_2_-1,15-lactone (**80**) and PGE_3_-1,15-lactone (**81**) are found in the cerata, detached when disturbed to distract potential predators and reverting to the free acid forms, PGE_2_ and PGE_3_, which are suggested to be the defensive compounds [237].

#### 2.7.2. Sacoglossa 

Many sacoglossans also release their cerata when disturbed. Among them, the Mediterranean *Ercolania viridis* presents γ- and α-pyrone polypropionates, including cyercenes (**113, 157–159**) [473]. Cyercene polypropionates (**113, 157–159**) are also found in the Mediterranean *Cyerce cristallina*, which also displays cerata autotomy [326,327], as well as in *C. nigricans* from Australia [328]. Moreover, in *Aplysiopsis formosa* from the Atlantic Ocean, the α-pyrones aplysiopsenes A–D (**183,184**) have been found [325]. The implication of polypropionates in autotomy and further cerata regeneration has been further demonstrated by experiments with *Hydra* [474]. However, some sacoglossa may also present cerata autotomy without possessing polypropionates, such as the Atlantic *Mourgona germaineae*, which instead possesses prenylated bromohydroquinones obtained from the calcareous green alga *Cymopolia barbata* [330], similarly to *Costasiella ocellifera* from *Avrainvillea longicaulis* [325]. In contrast, some species may present polypropionates without displaying autotomy behavior, such as *Placida dendritica*, which possesses polypropionate γ-pyrones (**116**) with no regenerative activity on the *Hydra* assay [332]. Evolutionary trends are probably behind this behavioral and chemical variability [2].

### 2.8. Other Ecological Activities

Some nudibranchs have been reported to display activities other than those mentioned above [1,2]. These may include metabolites that may be considered stress metabolites, as well as compounds involved in reproduction or in hormonal responses, as reported below (Figure 9, Table 9).

#### 2.8.1. Nudibranchia

##### Doridacea

The Antarctic slug *Doris kerguelenensis* possesses diterpenoid glycerols (**2**) and terpenoid glyceryl esters of different types (*ent*-labdane, halimane, isocopalane diterpenoids) in its mantle many [59,62,64,370,381,476]. *D. kerguelenensis* may also present some nor-sesquiterpenes in its mantle, austrodoral (**185**) and its oxidized derivative austrodoric acid (**186**), which have been detected in higher amounts after the animals were kept in aquarium for 15 days before freezing [64]. This is the reason why austrodoral (**185**) has been suggested to be a stress metabolite, although perhaps some physiological alterations may also be behind these higher amounts of austrodoral (**185**) [64].

##### Dendronotida

Prostaglandins (PGs) (**80–81**) from *Tethys fimbria* have been reported to be de novo biosynthesized, having different roles in the species, and being located in different body parts of the slug [237,238,239]. As previously mentioned, PGE_2_-1,15-lactone (**80**) and PGE_3_-1,15-lactone (**81**) are found in the cerata, reverting to the free acid forms PGE_2_ and PGE_3_ probably for defense [237]. In their reproductive system, instead, PGF–lactones are found, and these have been suggested to have a role in reproduction [239].

##### Euarminida

Some species have been reported to inhibit cell division in embryos or eggs of sympatric species to avoid competition and/or further predation by their adults, for example, against sea urchins or sea stars [1,2]. An example is *Dermatobranchus ornatus* from China, which presents several types of diterpenes that inhibit cell division in fertilized starfish eggs [22]. These compounds include four eunicellin diterpenes, such as ophirin (**187**). In fact, two of these compounds are probably taken from its gorgonian prey, *Muricella sinensis* [22], while another one was previously found in an unidentified Pacific soft coral [387]. Furthermore, *D. ornatus* possesses a calicophirin diterpenoid (**188**) probably from a gorgonian prey, *Muricella* sp. [477].

## 3. Pharmacological Activity

Marine natural products may have many diverse applications that are beneficial for humans and, thus, may become potentially very useful drugs [5,6,8,10,29,30,31,33]. Some recent reviews offer wide summaries of the different compounds and activities, as well as a historical perspective [31,32,33,35,36,37,38,39,40,42,43,123,478,479,480,481,482]. Overall, it remains clear that natural products are still the best option to find novel bioactive entities and potentially be modified to find leads to fight several human diseases. 

### 3.1. Cytotoxicity and Antitumoral Activity

**Figure 10:**
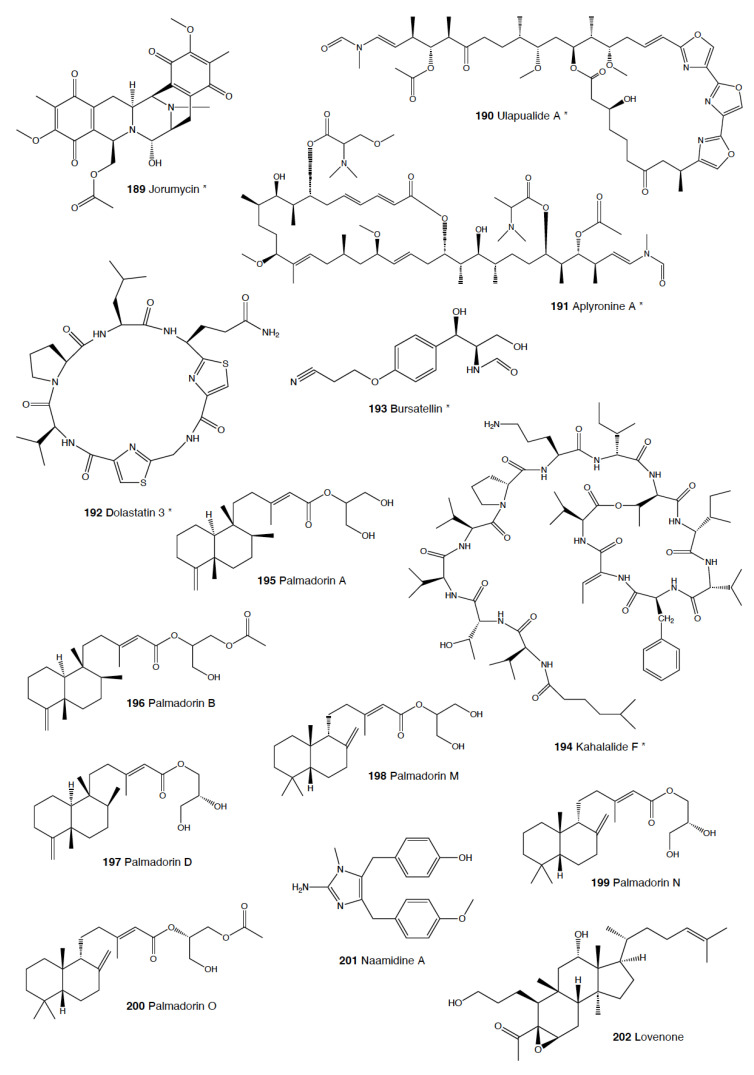
Structures of selected cytotoxic and antitumoral compounds in some heterobranch molluscs. These molecules may also display other activities, as reported in the text. * = compounds in clinical trials.

**Figure 11:**
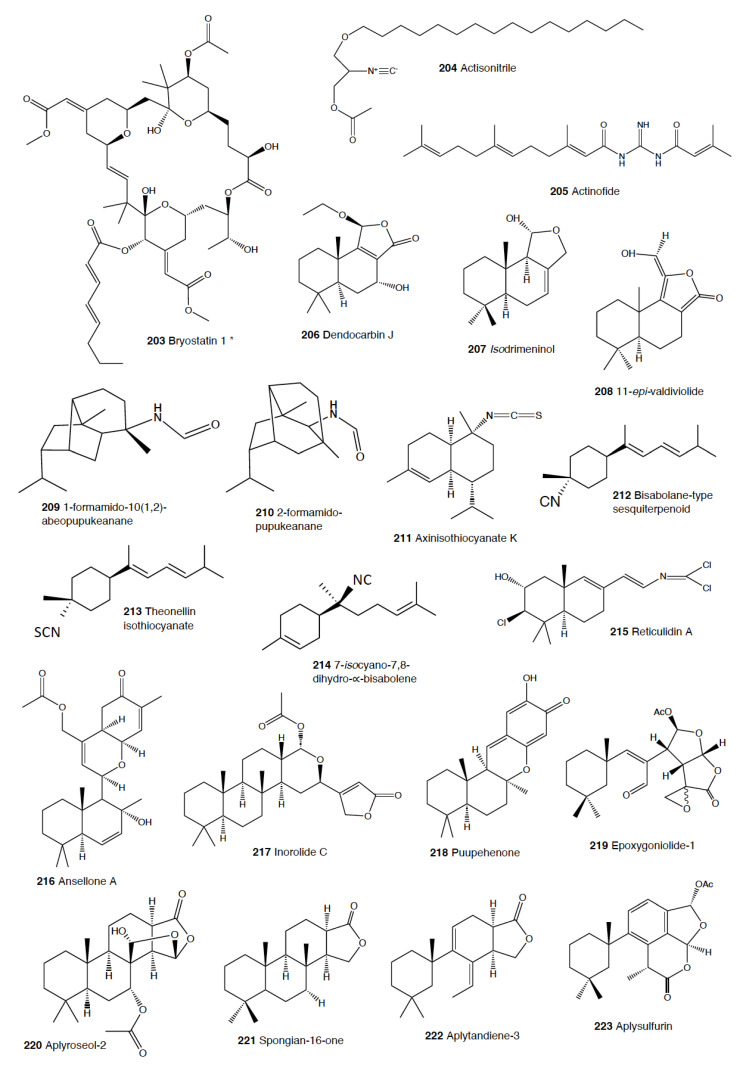
Structures of selected cytotoxic and antitumoral compounds in some Doridacea nudibranchs. These molecules may also display other activities, as reported in the text. * = compounds in clinical trials.

**Figure 12:**
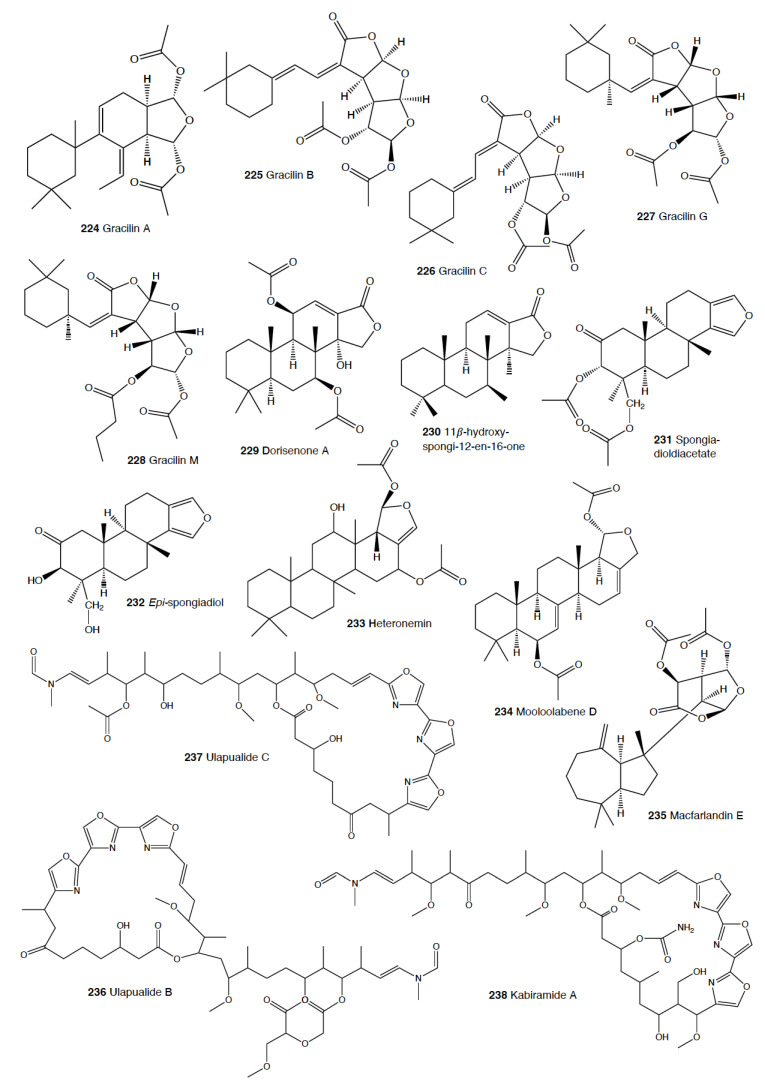
Structures of selected cytotoxic and antitumoral compounds in some Doridacea nudibranchs. These molecules may also display other activities, as reported in the text.

**Figure 13:**
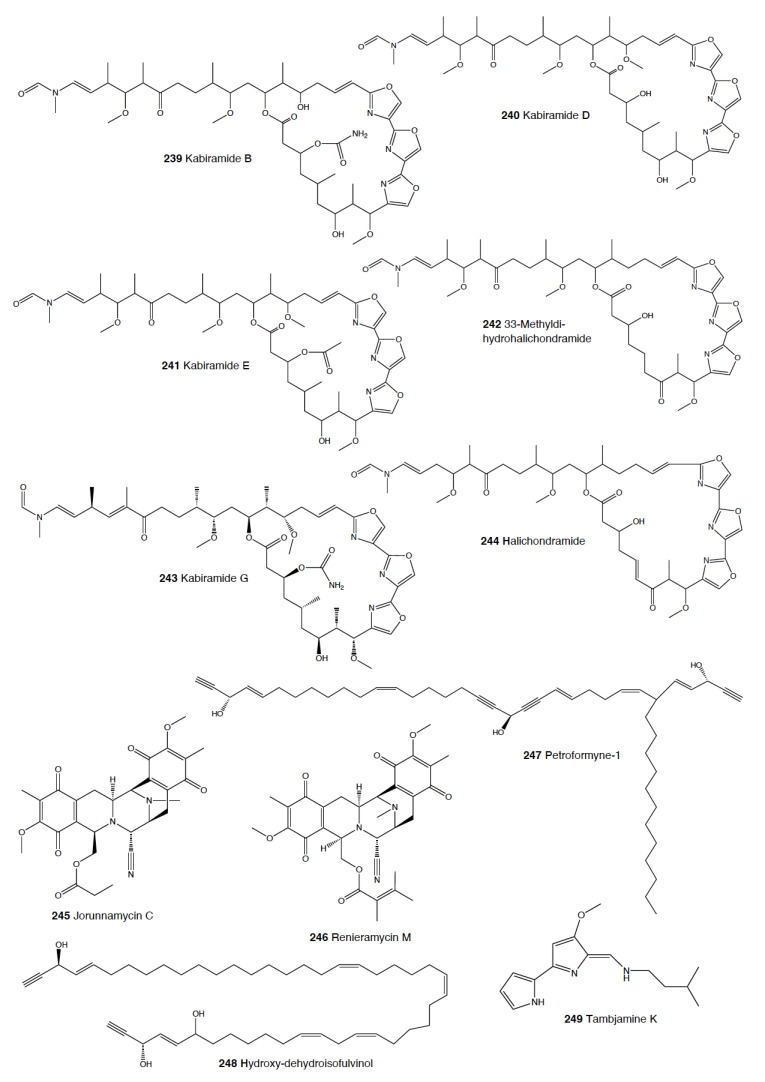
Structures of selected cytotoxic and antitumoral compounds in some Doridacea nudibranchs. These molecules may also display other activities, as reported in the text.

**Figure 14:**
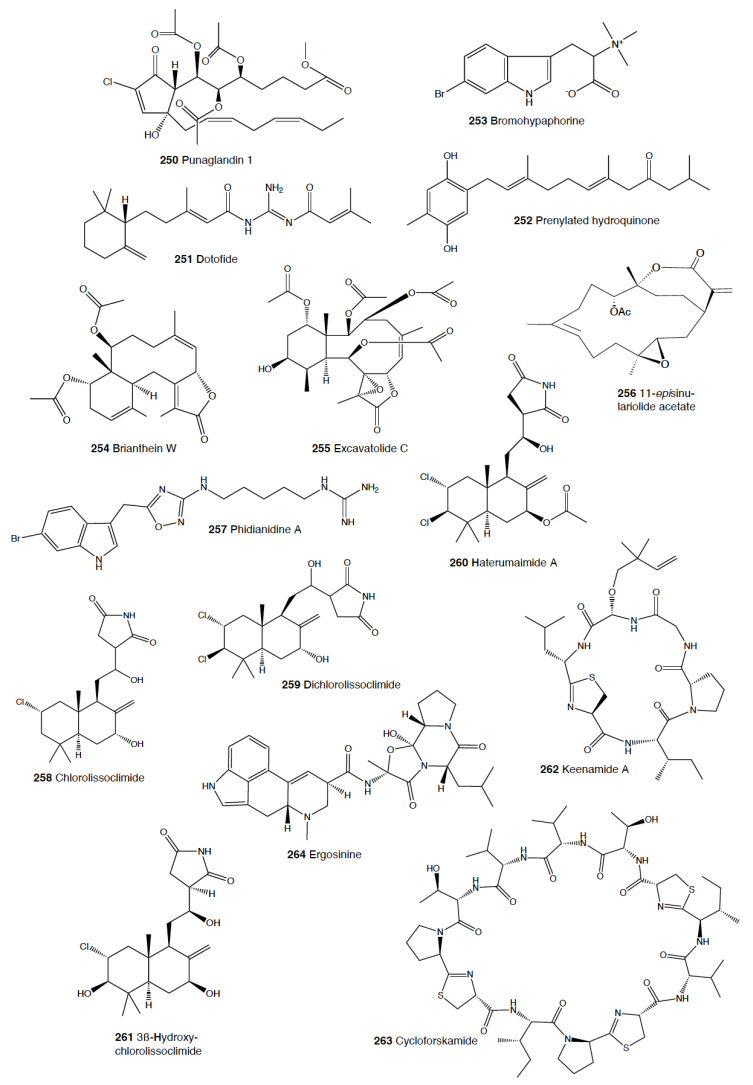
Structures of selected cytotoxic and antitumoral compounds in some Dendronotida, Euarminida, and Aeolidida nudibranchs, and Pleurobranchoidea. These molecules may also display other activities, as reported in the text.

**Figure 15:**
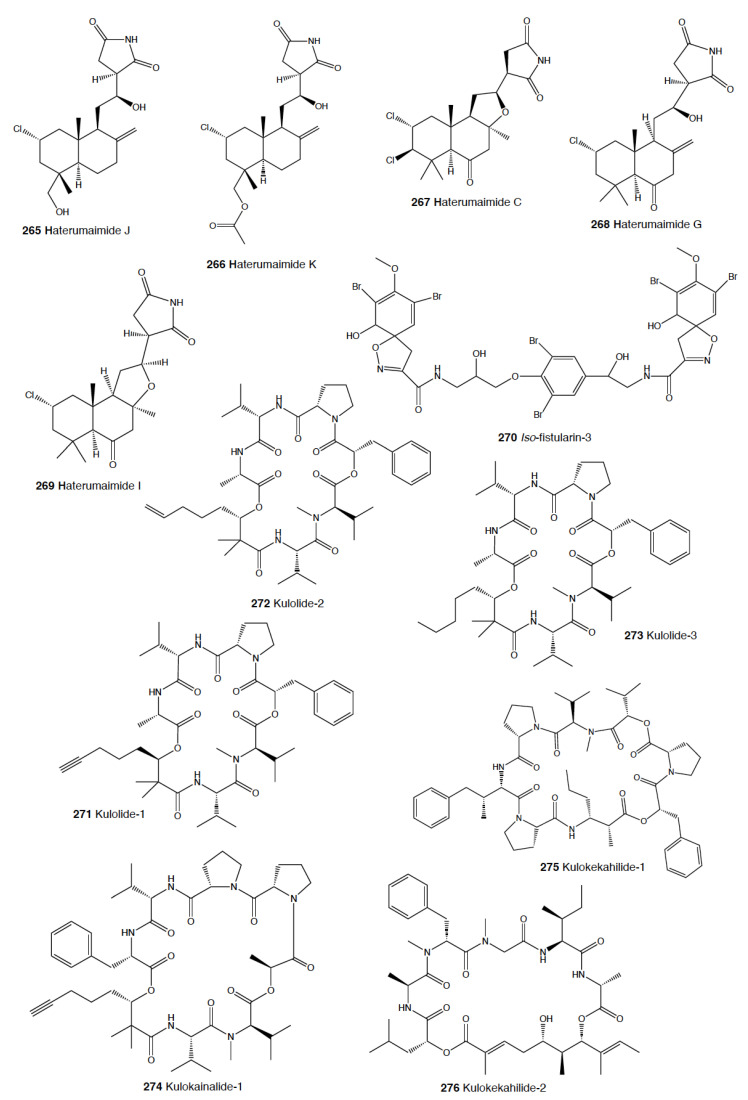
Structures of selected cytotoxic and antitumoral compounds in some Pleurobranchoidea, Tylodinoidea, and Cephalaspidea. These molecules may also display other activities, as reported in the text.

**Figure 16:**
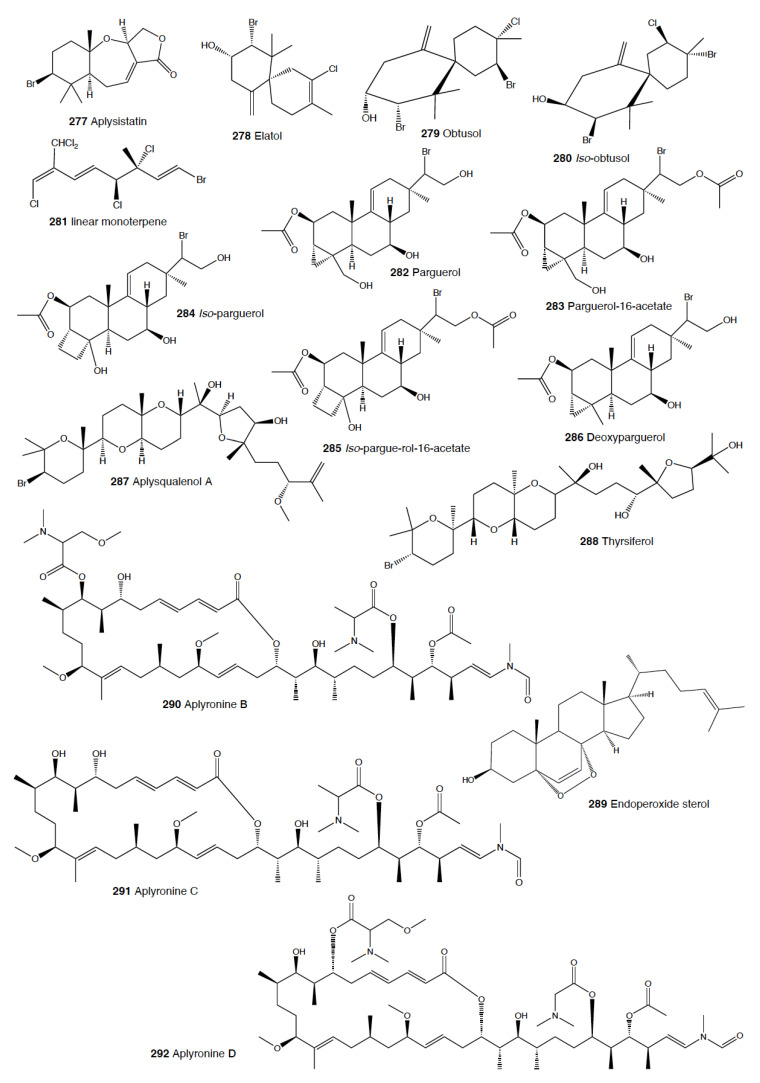
Structures of selected cytotoxic and antitumoral compounds in some Anaspidea. These molecules may also display other activities, as reported in the text.

**Figure 17:**
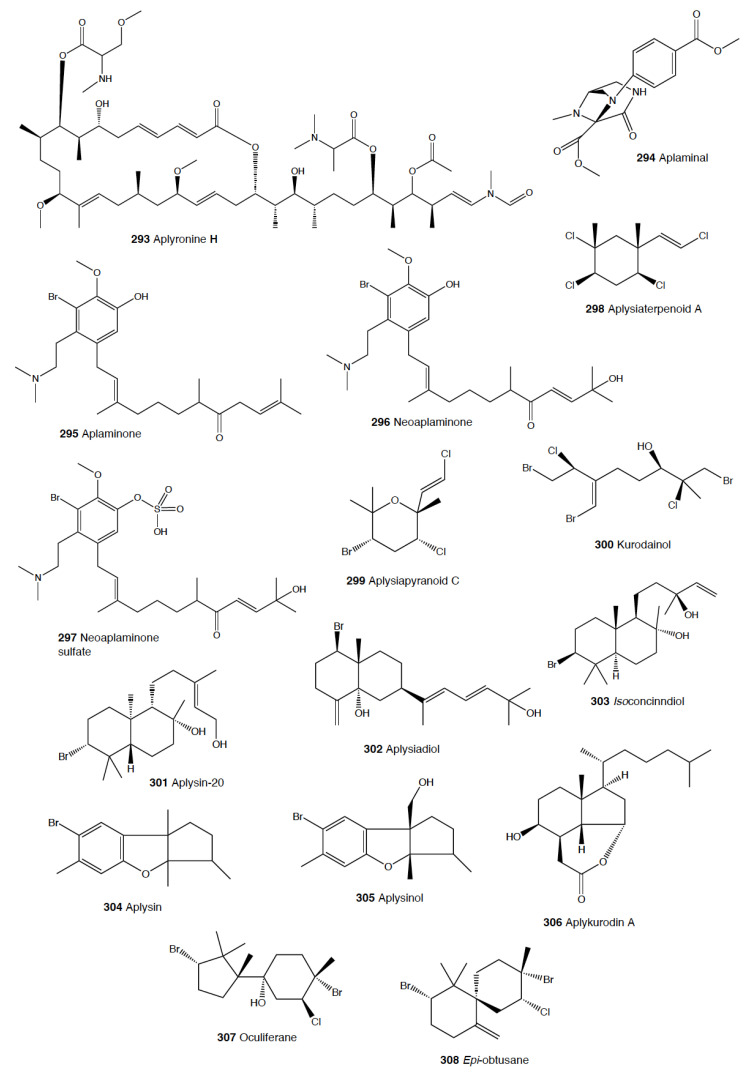
Structures of selected cytotoxic and antitumoral compounds in some Anaspidea. These molecules may also display other activities, as reported in the text.

**Figure 18:**
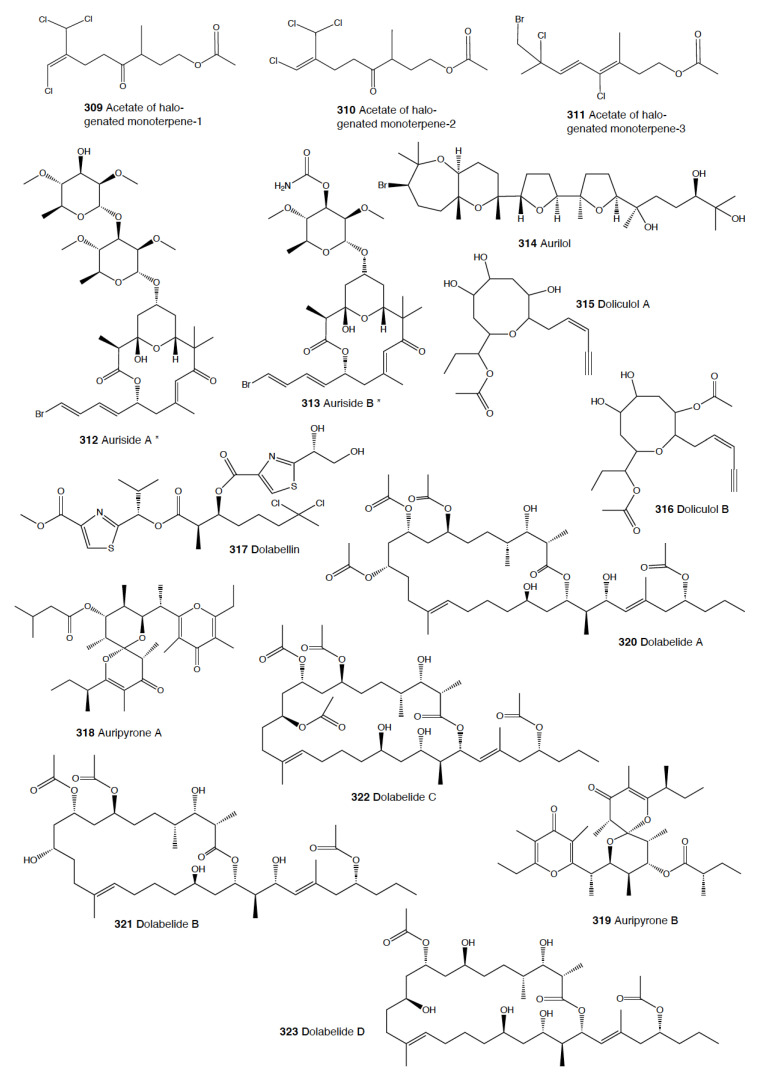
Structures of selected cytotoxic and antitumoral compounds in some Anaspidea. These molecules may also display other activities, as reported in the text. * = compounds in clinical trials.

**Figure 19:**
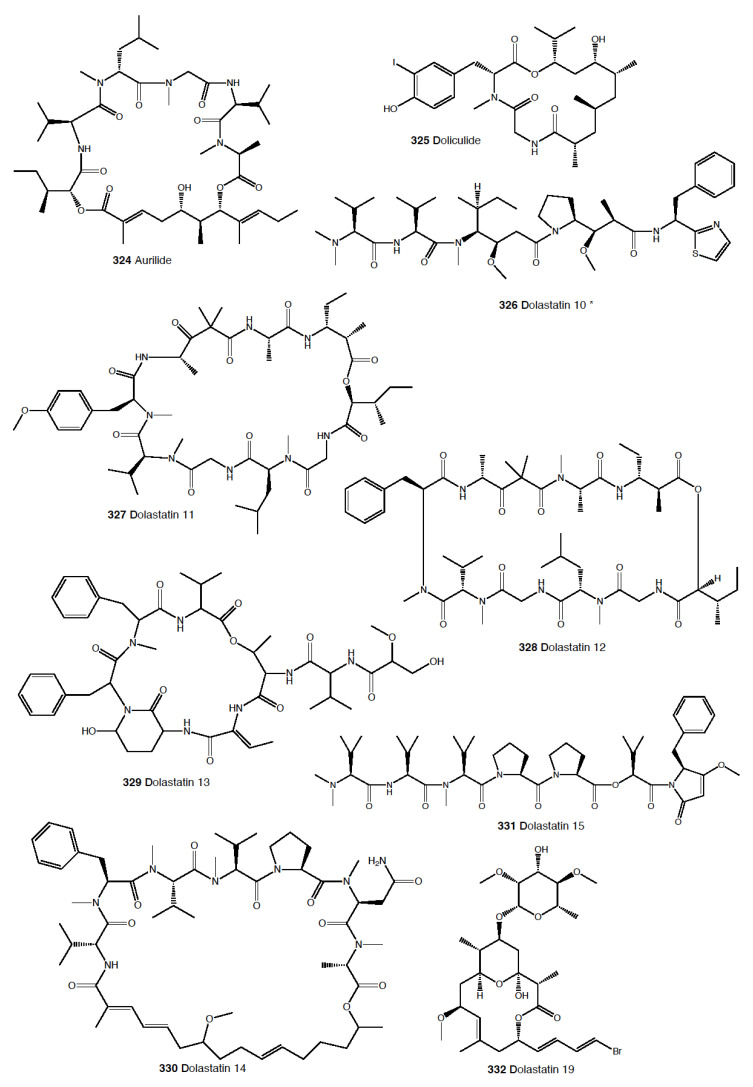
Structures of selected cytotoxic and antitumoral compounds in some Anaspidea. These molecules may also display other activities, as reported in the text. * = compounds in clinical trials.

**Figure 20:**
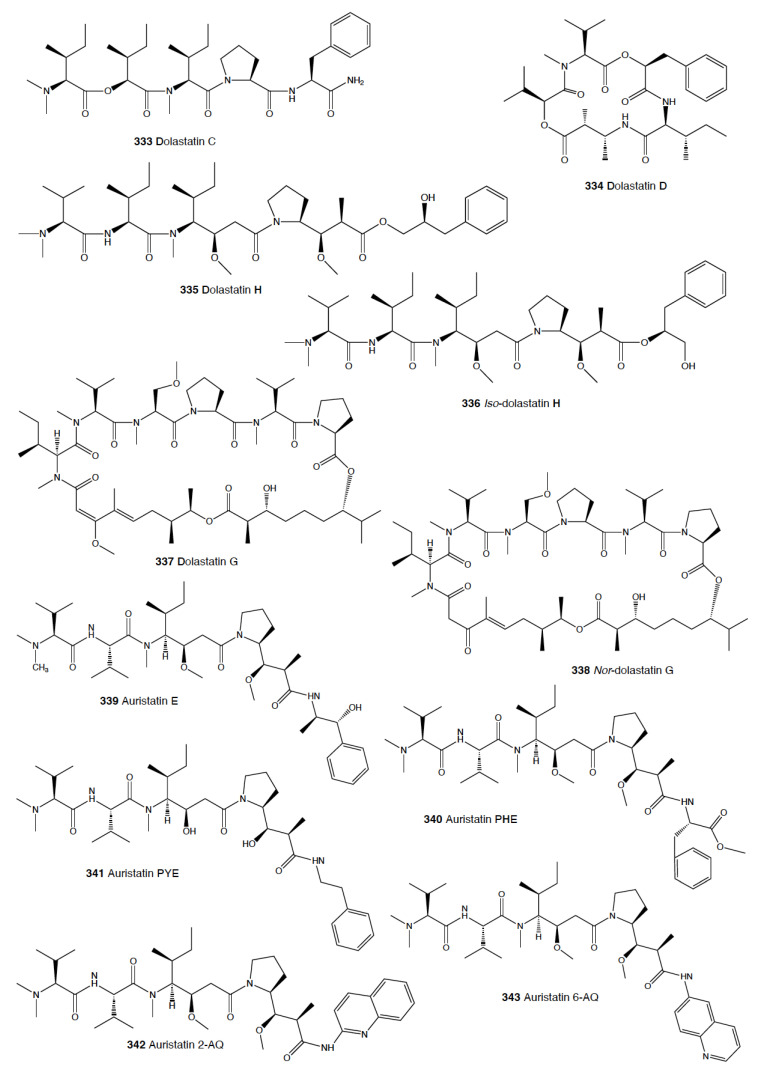
Structures of selected cytotoxic and antitumoral compounds in some Anaspidea. These molecules may also display other activities, as reported in the text.

**Figure 21:**
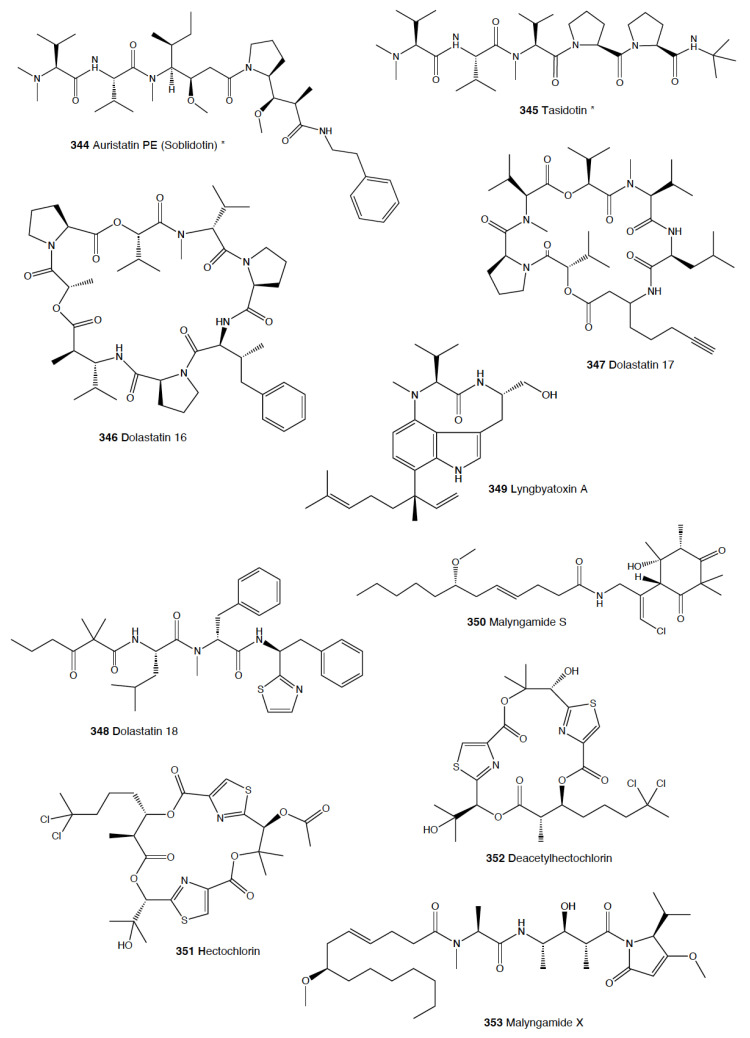
Structures of selected cytotoxic and antitumoral compounds in some Anaspidea. These molecules may also display other activities, as reported in the text. * = compounds in clinical trials.

**Figure 22:**
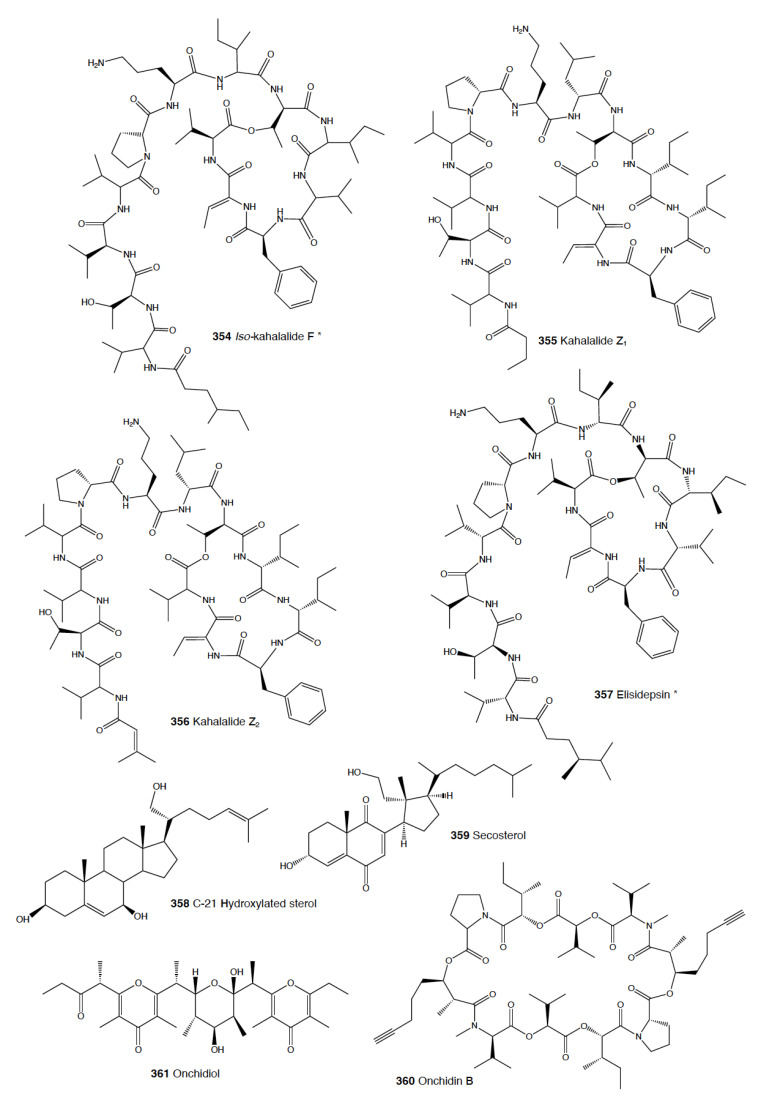
Structures of selected cytotoxic and antitumoral compounds in some Sacoglossa and Pulmonata. These molecules may also display other activities, as reported in the text. * = compounds in clinical trials.

**Figure 23:**
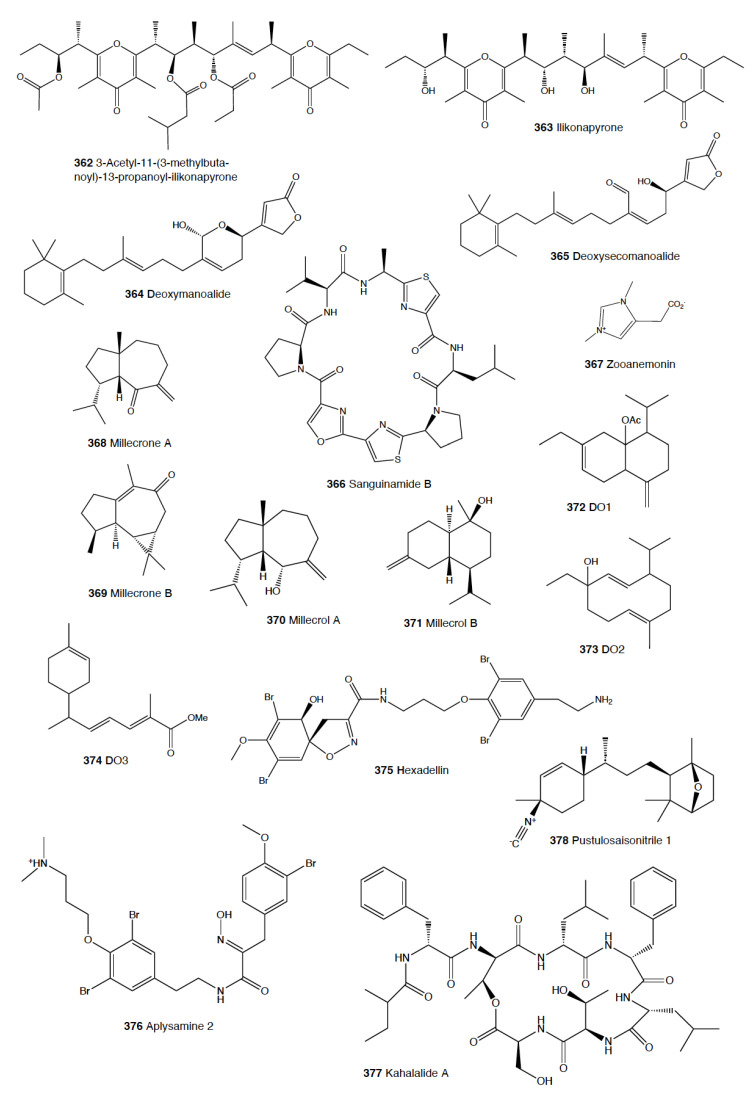
Structures of selected cytotoxic and antitumoral compounds in some Pulmonata, as well as selected antibiotic compounds in Doridacea, Euarminida, Tylodinoidea, Anaspidea, and Sacoglossa, and an antiparasitic compound from a Doridacea. These molecules may also display other activities, as reported in the text.

Many reviews have dealt with this topic in depth, and, therefore, we include here only a brief summary of all the activities described [31,42,479]. Among the most active compounds, it is worth mentioning jorumycin (**189**) from the doridacean *Jorunna funebris*, ulapualides (**190**) from *Hexabranchus sanguineus*, kabiramides (**62**) from *Hexabranchus* sp., aplyronines (**191**) and dolastatins (**192**) from the anaspidean *Dolabella auricularia*, and bursatellanins (**193**) from *Bursatella leachii*, as well as kahalalides (**194**) from the sacoglossan *Elysia rufescens*, all of which are under clinical trials for antitumoral activity [36,43,45,46,479]. It is important to remember that cytotoxic, anticancer, and antitumoral compounds are strongly needed, since cancer remains one of the deadliest diseases worldwide [42]. In 2018, about 18 million new cancer cases were reported globally, producing around 10 million deaths [483]. Thus, many studies are being developed to find new compounds with novel modes of action, and marine organisms, such as heterobranch molluscs, are considered to be reservoirs of new bioactive compounds that are potentially useful [42]. In fact, about 30% of the top twenty drugs currently on the market originate from a natural source (mostly plants), while approximately 50% of the marketed drugs are naturally derived or based on natural compounds [31,480,484]. Comparing marine and terrestrial natural products, it has been reported by the NCI (USA) that more than 1% of marine compounds present antitumoral activity compared to the 0.01% for terrestrial compounds [481]. Many natural compounds present cytotoxic effects against macromolecules expressed by cancer cells, such as in oncogenic signal transduction pathways, and display growth inhibition of human tumor cells [39,42,43,482]. Moreover, many studies on the mechanism of action of marine natural compounds inhibiting tumor growth indicate that it involves processes of apoptosis, necrosis, and lysis of the tumor cells, both in vitro and in vivo [42,485]. All heterobranch groups reviewed here possess some anticancer, antitumoral, and/or cytotoxic compounds, except pteropods (Figure 10, Figure 11, Figure 12, Figure 13, Figure 14, Figure 15, Figure 16, Figure 17, Figure 18, Figure 19, Figure 20, Figure 21, Figure 22 and Figure 23, Table 10). The natural products described include a wide variety of compounds, from terpenoids and steroids, to peptides, polyketides, as well as nitrogen-containing metabolites.

#### 3.1.1. Nudibranchia

##### Doridacea

The Antarctic slug *Doris kerguelenensis* presents clerodane and labdane diterpenes, such as palmadorins (**195–200**), among other compounds [66,67]. Palmadorins A (**195**), B (**196**), D (**197**), M (**198**), N (**199**), and O (**200**) have been described to inhibit human erythroleukemia cells (HEL) at low IC_50_ (micromolar), and palmadorin M (**198**) has been reported to inhibit Jak2, STAT5, and Erk1/2 activation in HEL cells, producing apoptosis at 5 μM [67]. The Mediterranean and Atlantic *Doris verrucosa*, contains the de novo biosynthesized verrucosins, diterpenoid acid glycerides, among which verrucosins A (**124**) and B are potent activators of protein kinase C, and they promote tentacle regeneration in the freshwater hydrozoan *Hydra vulgaris* [370,371,486]. *Notodoris* nudibranchs feed on *Leucetta* calcareous sponges, presenting sponge-derived imidazole alkaloids [424,426,487,488]. In the Red Sea, *N. citrina* and *N. gardineri* obtain their chemicals from *Leucetta chagosensis*, presenting among others, naamidine A (**201**) and *iso*-naamidine-A (**160**) [424,426]. Naamidine A (**201**) was later tested from the sponge (from different localities) as a selective inhibitor of the epidermal growth factor (EGF) and was found to inhibit human tumor xenografts in mice, as well as displaying antitumour activity that promotes caspase-dependent apoptosis in tumor cells [489,490].

*Adalaria loveni* from the North Sea presents lovenone (**202**), a degraded triterpenoid suggested to come from an unidentified bryozoan prey [491]. Lovenone (**202**) was reported to present modest cytotoxicity to two HTCLs (human tumor cell lines) [491]. Another bryozoan-feeder, *Polycera atra* accumulates bryostatins (**203**) from *Bugula neritina*, also including them in their spawn [340,492,493,494]. Bryostatins (**203**) are polyketide macrolides known to be biosynthesized by a microbial symbiont, *Endobugula sertula* [495]. Bryostatins (**203**) are highly bioactive compounds, with bryostatin 1 (**203**), for example, being investigated in more than 20 clinical trials (two phase I trials) against multiple carcinomas and Alzheimer’s disease [31,42,123,496,497]. Bryostatin 1 (**203**) modulates the Bcl-2 and p53 oncoproteins in human diffuse large-cell lymphoma WSU-DLCL2, inducing a decrease in the expression of Bcl-2 [498]. Furthermore, bryostatin 1 (**203**) showed a notable activity of modulating the paclitaxel inhibitor of protein kinase C (PKC) [499,500,501], as well as inducing ubiquitination and proteasome degradation of Bcl-2 in lymphoblastic leukemia, allowing for the growth of progenitor cells from bone marrow [502]. Moreover, bryostatins (**203**) are strong activators of PKC, regulating the activation, growth, and differentiation of cells [503].

Another interesting species is the Chinese *Actinocyclus papillatus*, which presents the mildly cytotoxic (–)-actisonitrile (**204**) in its mantle, along with actinofide (**205**), a terpenoid diacylguanidine [361,504,505]. Both enantiomers, (–)- and (+)-actisonitrile (**204**), were tested for cytotoxicity against tumor and non-tumor mammalian cells, resulting in a parallel concentration-dependent toxic profile at a micromolar concentration [504]. On the other hand, actinofide (**205**), a guanidine moiety acylated by two terpenoid acid units, allowed for the synthesis of a series of structural analogues which were tested for growth inhibitory activity against some cancer cell lines in vitro [506,507,508]. Actinofide (**205**) and some of its analogues were tested against six cancer cell lines: two human carcinoma cancer cell lines (MCF-7 (breast) and A549 (non–small-cell lung cancer, NSCLC)) of epithelial origin, two human cancer cell lines from glial origin (Hs683 oligodendroglioma and U373 glioblastoma of astrocytic origin), and two melanoma models (mouse B16F10 and human SKMEL-28 cell lines), resulting in many relevant activities [479]. Actinofide (**205**) presents GI_50_ values of 8.3 ± 1.8 for Hs683, 15.7 ± 10.1 for U373, 23.4 ± 5.5 for A549, 23.4 ± 5.9 for MCF-7, 24.2 ± 8.2 for SKMEL-28, and 7.5 ± 3.1 for B16F10 [479,505]. The most active compounds are reported to be those with one or two N-C_15_ residues, and a preliminary correlation between structure and activity was proposed [479].

*Aldisa andersoni* from the Indo-Pacific contains some phorbazoles, peculiar chlorinated phenyl-pyrrolyloxazoles, such as 9-chloro-phorbazole D (**5**) and *N*1-methyl-phorbazole A (**6**), along with phorbazoles A (**7**), B (**8**), and C described in the sponge *Phorbas* aff. *clathrata* [55,56,75,76]. Both 9-chloro-phorbazole D (**5**) and *N*1-methyl-phorbazole A (**6**) produce cytostatic effects in vitro against several HTCLs [75]. More specifically, *N*1-methyl-phorbazole A (**6**) inhibits human SKMEL-28 melanoma and U373 glioblastoma cells [75].

*Dendrodoris carbunculosa* from Japan possesses drimane sesquiterpenoids, the dendocarbins A–N (**16,206**), along with *iso*drimeninol (**207**) and 11-*epi*valdiviolide (**208**), some of them displaying cytotoxicity against murine leukemia P388 cell lines [87]. In particular, the IC_50_ values against adriamycin (ADR)- and vincristine (VCR)-resistant P388 cells (P388/ADR and P388/VCR, respectively), as well as those against sensitive P388 strain (P388/S) were reported for all compounds, with dendocarbin J (**206**) and 11-*epi*valdiviolide (**208**) showing moderate cytotoxicity against both sensitive and resistant cell strains [86]. The IC_50_ values for dendocarbin J (**206**) were 17 µg/mL for P388/S, 4 µg/mL for P388/VCR(-), 4 µg/mL for P388/VCR(+), 11 µg/mL for P388/ADR(-), and 8 µg/mL for P388/ADR(+), while for 11-*epi*valdiviolide (**208**) the values were 3.2, 2.5, 2.5, 2.5, and 2.5 µg/mL, respectively [86]. The origin of 11-*epi*valdiviolide (**208**) could be dietary, since it was first described in the sponge *Dysidea fusca* [509].

Regarding phyllidids, several species are described to present bioactive compounds. In Thailand, *Phyllidia coelestis* presents two cytotoxic pupukeanane sesquiterpenoids, 1-formamido-10(1,2)-abeopupukeanane (**209**) and 2-formamidopupukeanane (**210**), which show in vitro growth inhibitory activity against four human cancer cell lines [111]. In particular, this activity was tested against HeLa (cervical), MCF-7 (breast), KB (oral cavity), and HT-29 (colon) cancer cell lines with IC_50_ values between 0.05 and 10 μM [111,510]. Furthermore, both molecules (**209,210**) present selectivity, weakly inhibiting the growth of human gingival fibroblasts by 65 and 25% at 20 μM, respectively [111,510]. *Phyllidiella pustulosa* also contains many sesquiterpenoids, as abovementioned, and specimens from Okinawa, for example, possess the moderately cytotoxic substituted axinisothiocyanate K derivative (**211**) and an isocyano compound [377]. In Fiji, *P. pustulosa* contains the isothiocyanate axisonitrile-3 (**25**), which is weakly cytotoxic (IC_50_ > 20 μg/mL), in addition to some related sesquiterpenes [118,427]. In southern China, both *P. coelestis* and *P. pustulosa* present several nitrogenous terpenoids from their demosponge prey, *Acanthella cavernosa* [117]. Among these compounds, a bisabolane-type sesquiterpenoid (**212**), a theonellin *iso*thiocyanate (**213**), and 7-*iso*cyano-7,8-dihydro-∝-bisabolene (**214**) display cytotoxicity against several HCCLs [117]. In particular, they all show strong cytotoxicity against HCCL SNU-398 with IC_50_ values of 0.50, 2.15, and 0.50 µM, respectively [117]. Furthermore, the bisabolane sesquiterpenoid (**212**) presents broad cytotoxicity, being active against HCCLs A549, HT-29, and Capan-1, with IC_50_ values of 8.60, 3.35, and 1.98 µM, respectively [117]. Contrastingly, *Reticulidia fungia* from Okinawa presents the cytotoxic carbonimidic dichlorides, reticulidins A (**215**) and B, which are two uncommon sponge sesquiterpenes, probably obtained from their diet of *Pseudaxinyssa* sponges [438,511,512]. Both compounds are moderately cytotoxic in vitro against KB cells, with IC_50_ values of ~1 μM for both, and against mouse L1210 leukemia cells, with IC_50_ values of ~2 and ~0.3 μM, respectively [438]. 

Chromodoridids are also reported to possess cytotoxic and anticancer compounds. In the Paficic, *Cadlina luteomarginata* and its sponge prey *Phorbas* sp. Contain, among other compounds, ansellone A (**216**), a sesterterpenoid that moderately activates the cyclic adenosine monophosphate (cAMP) signaling pathway, with an EC_50_ value of 14 mM in the HEK293 cell-based test [513]. The modulation of the cAMP signaling pathway is used in stem cell techniques, and it is relevant to treat diseases such as cancer, heart failure, and neurodegenerative diseases [514]. On the other hand, several *Chromodoris* species possess latrunculin A (**38**), a PKS-NRPS-derived macrolide, reported to be cytotoxic [141]. Latrunculin A (**38**) was found in *C. lochi* and its sponge prey *Spongia* (*Cacospongia*) *mycofijiensis* in Fiji [141]. In fact, latrunculins A (**38**) and B (**37**) were first described from the Red Sea *Negombata* (*Latrunculia*) *magnifica* and later in other sponges (*Hyattela* sp.) [168]. Latrunculins (**37,38**) were then reported in several *Chromodoris* species at different localities, including *C. africana, C. annae*, *C. elisabethina*, *C. hamiltoni*, *C. kuiteri*, *C. magnifica*, and *C. quadricolor* [153,155,164,170,171]. *C.* (*Glossodoris*) also obtains latrunculin B (**37**) from the demosponge *Latrunculia magnifica* [171]. Latrunculins (**37,38**) interfere with the cytoskeleton, disrupting the organization of cell microfilaments, and inhibit the proliferation of cancer cells due to their strong actin binding properties [153,164,515,516,517]. A PKS-NRPS-derived mycothiazole (**129**) found in *C. lochi* from Vanuatu and its prey sponge (see above) possesses selective cytotoxicity, inhibits the hypoxia-inducible factor-1 (HIF-1), and also suppresses the mitochondrial respiration at complex I in sensitive cell lines (IC_50_ values of 0.36–13.8 nM for HeLa, P815, RAW 264.7, MDCK, HeLa S3, 4T1, B16, and CD4/CD8 T cells) [103,104,105,106]. Latrunculins A (**38**) and B (**37**) display antimigratory activity against highly metastatic human prostate cancer PC-3M-CT^+^ cells and murine brain-metastatic melanoma B16B15b cells [515,518]. Latrunculin A (**38**) presented IC_50_ values of ~0.5 μM against murine P388 leukemia, human HT-29 colon cancer, and human A549 NSCLC, with more than a fivefold in vitro growth inhibitory effects against A549 NSCLC than to P388 leukemia [519]. Latrunculin A (**38**), coded NSC613011 on the NCI database, has an IC_50_ mean value of ~0.7 μM in the 60 cancer cell line panel, with a more than twofold log magnitude difference between the most sensitive and the most resistant cancer cell lines, being as active against MDR NCI/ADR-RES as against cells without the MDR phenotype [47]. Finally, latrunculin A (**38**) induces apoptosis in cancer cells via activation of the caspase-3/caspase-7 pathway and displays strong anticancer effects in peritoneal dissemination models of MKN45 and NUGC-4 human gastric cancer in mice [455]. In vivo anticancer assays using A549 NSCLC xenografts also show that latrunculin A (**38**) increased the life span of treated tumor-bearing mice by 46% compared to controls [520].

*Chromodoris lochi* from Indonesia, instead, presents sponge-derived polyketides, such as laulimalide (**39**) and *iso*laulimalide (**40**) from the sponge *Hyattella* sp., both compounds being cytotoxic due to their microtubule-stabilizing action at a different binding site to taxanes, located on two adjacent β-tubulin units between tubulin protofilaments of a microtubule [521,522]. Therefore, these compounds (**39–40**), with IC_50_ values of 15 ng/mL in the KB cell line, are being tested as potential antitumor agents [142,523,524]. Laulimalide (**39**) inhibits growth in more than ten cancer cell lines at low nanomolar concentrations, while *iso*laulimalide (**40**) is effective at low micromolar values [142,525,526]. As previously mentioned, laulimalide (**39**) is a microtubule stabilizer, like the plant compounds taccalonolide and paclitaxel, but laulimalide (**39**) has been reported to cause the formation of aberrant, structurally distinct mitotic spindles, differently from the other two molecules [527]. Moreover, laulimalide (**39**) has an effect in P-gp-overexpressing cancer cells and against cell lines resistant to paclitaxel and epothilones [525,528]. Further studies have shown that in ovarian cancer cells, the increased expression of βII- and βIII tubulin isotypes induces resistance to laulimalide (**39**), as does the downregulation of vimentin expression in human ovarian carcinoma cells [529,530]. Moreover, assays in vivo tested the anticancer activity of laulimalide (**39**) in two xenograft models, the human MDA-MB-435 breast cancer and the human HT-1080 fibrosarcoma models, describing little tumor growth inhibition accompanied by a strong toxicity and mortality, contrasting to paclitaxel [525]. 

Furthermore, inorolides A–C (**217**), sesquiterpenoids found in the Japanese *Chromodoris inornata* (*C. aspersa*) and other scalarane terpenoids are cytotoxic against murine L1210 leukemia and human epidermoid carcinoma KB cell lines [156]. Particularly, inorolides A–C (**217**) display IC_50_ values of ~7, ~5, and ~4 μM, respectively, while sesterterpenoids like deoxoscalarin (**46**) and its analogues, deoxoscalarin-3-one, 21-hydroxydeoxoscalarin, 21-acetoxydeoxoscalarin, and 12-O-acetyl-16-O-deacetyl-12,16-episcalarolbutenolide, display values of ~3, ~2, ~9, ~1, and ~5 μM, respectively [156]. Some of these metabolites have been reported also in *Hyrtios* sponges and reviewed for their bioactivity [156,531]. 

Similarly, cytoxicity has been reported in several compounds from other *Chromodoris* species, such as a sponge diterpene found in *C. petechialis* from Hawai’i, puupehenone (**218**), an oxygenated diterpene of *C. elisabethina* from Australia, a spongian diterpene of *C. kunei* from Okinawa, furanoditerpenoids found in *C. reticulata* from China, and the mildly toxic diterpenes from a *Chromodoris* sp. from Australia [22,154,158,160,186]. Puupehenone (**218**) is active at IC_50_ values of 1 μg/mL to P388, 0.1–1 μg/mL to A-549 and MCF-7, as well as 1–10 μg/mL to HCT-8 [22,154,158,160,186].

*Goniobranchus* species also possess some cytotoxic compounds. In Australia, *G. splendidus* has a cytotoxic spiroepoxide lactone, epoxygoniolide-1 (**219**), suspected to originate from its dietary sponge prey [532]. Epoxygoniolide-1 (**219**) shows moderate cytotoxicity to NCIH-460, SW60, and HepG2 cancer cells [532]. *G. (Chromodoris) sinensis* from China presents aplyroseol-2 (**220**) [131]. *G. reticulatus* from Australia presents a dialdehyde sesquiterpene, together with the ring-closed acetal, both bioactive against P388 mouse leukemia cells [161,186]. *G. reticulatus* contains spongian-16-one (**221**), aplytandiene-3 (**222**), aplysulfurin (**223**), aplyroseol-2 (**220**), and gracilins A (**224**), B (**225**), C (**226**), G (**227**), and M (**228**), all of which display cytotoxicity against HeLa S3 cells [190]. Moreover, gracilins B (**225**) and C (**226**), as well as some isomers, obtained from the demosponge *Spongionella* sp. are cytotoxic against a wider panel of HTCLs, and have also been reported as cyclosporine A mimics and BACE1 and ERK inhibitors (see below) [533,534]. Gracilins M–Q (**228**) showed a significant potency against the HeLa S3 cell line [113]. In Japan, *G. (Chromodoris) obsoletus* contains dorisenones A–D (**229**), cytotoxic sponge diterpenoids, along with related compounds, such as 11*β*-hydroxyspongi-12-en-16-one (**230**), spongian-16-one (**221**) [154]. All of these compounds are active against murine lymphoma L1210 and human epidermoid carcinoma KB cells at IC_50_ submicromolar (as low as IC_50_ 0.2 μg/mL) and low micromolar values, respectively, and although dorisenone D and a related compound have also been tested in vivo against P388 leukemia, they show no activity [154]. Similarly, in *G. collingwoodii*, some spongian-16-one diterpenes are reported to be inactive to a range of HTCLs [186].

The spongian diterpenes of *Glossodoris cincta* from Egypt and Sri Lanka have also been reported to be cytotoxic [22,166,380,381,535,536,537]. The compound 12-*epi*-scalaradial found in *G. cincta* and *G. hikuerensis* is the most active, inhibiting the epidermal growth factor receptor (EGFR) implicated in many cancers, and also inhibiting the human recombinant PLA2 at 0.02 μM. *Doriprismatica (Glossodoris) atromarginata* presents furanoditerpenoids and scalarane sesterterpenes originating from its dietary sponges *Spongia* (*Hyatella*) sp. and *Hyrtios* spp., depending on the geographical location (Australia, Sri Lanka, India) [93,176,181,386,387,388]. The most active metabolites were spongiadiol (**35**), spongiadiol diacetate (**231**), *epi*-spongiadiol (**232**), 12-deacetoxy-12-oxodeoxoscalarin (**136**), heteronemin (**233**), and mooloolabene D (**234**) [180,381,383,384,385,386,519,535,538,539,540]. In particular, spongiadiol (**35**) is active against P388 murine leukemia cells at an IC_50_ of 0.5 μg/mL. Other species, such as *Felimida (Glossodoris) dalli*, *Doriprismatica (Glossodoris) sedna*, *Glossodoris rufomarginata*, *Glossodoris pallida*, *Glossodoris vespa*, and *Ardeadoris (Glossodoris) averni*, also present homoscalarane and scalarane metabolites, among which 12-deacetyl-23-acetoxy-20-methyl-12*-epi*-scalaradial (**135**) moderately inhibits mammalian phospholipase A2 (IC_50_ = 18 μM) [175,183,383]. Heteronemin (**233**), also found in several chromodoridid species and derived from dietary sponges, such as *Heteronema erecta,* blocks tumor cell intravasation through the lymph-endothelial barrier in a three-dimensional (3D) cell culture model, using spheroids of the MCF-7 breast cancer cell [541,542].

*Hypselodoris infucata* from Bali presents (–)-furodysinin (**48**), which is active against the HeLa cell line with an IC_50_ of 102.7 μg/mL [214]. *Felimida (Chromodoris) macfarlandi* from California presents macfarlandines A–E (**134,235**), from which macfarlandin E (**235**) displays unique Golgi-modifying properties [139,140,149,543].

*Hexabranchus sanguineus* is a chemically rich species, transferring several compounds to their egg masses, including the macrolides ulapualides A (**190**) and B (**236**) in Hawai’i, presenting three contiguous oxazole rings with the attached lipid-like side chain ending in the N-methyl-N-alkenylformamide group [222]. Ulapualide A (**190**) has also been found in sponges and, thus, is suggested to be of dietary origin in the slug [544]. Both compounds are reported to display activity against murine L1210 leukemia cells, with IC_50_ values of ~10 and ~30 nM, respectively [222]. Ulapualide A (**190**) has been described to be a potent actin-depolymerizer [545]. Later, ulapualides C–E (**237**) have been found in egg masses from Hawai’i, and ulapualide C (**237**) is reported to display cytotoxicity against several HTCLs, although it is 2–4 times less potent than ulapualides A (**190**) and B (**236**) [546]. A different study found several kabiramides, including kabiramide A (**238**), B (**239**), D (**240**), and E (**241**), along with the halichondramide derivatives, dihydrohalichondramide (**63**) and 33-methylhalichondramide (**242**), in egg masses of the same species from a different locality [217,218,219,220,221]. Kabiramides (**238–241**) contain a macrolide ring with contiguous trisoxazole rings [547]. Kabiramide C (**62**) was traced to a *Halichondria* sponge and further reported in the sponge *Pachastrissa nux* together with other kabiramides, as well as to some adult slug specimens at a lower concentration [221,222,548]. Kabiramide C (**62**) presents growth inhibitory effects in vitro against human MCF-7 breast cancer cells (IC_50_ ~0.5 μM), ten times higher than for human fibroblasts (IC_50_ ~8 μM), displaying bioselectivity [548]. The mechanism of action has been described as kabiramide C (**62**) binding to actin and its actin complex, which is achieved through a two-step binding reaction and forms a very stable and long-lived complex [549]. Kabiramide G (**243**) presents an even stronger bioselectivity, with IC_50_ values of 0.02 μM against MCF-7 cancer cells and >2 μM for human fibroblasts [548]. Kabiramides A (**238**) and B (**239**) were active with IC_50_ values of ~10 nM, and kabiramides D (**240**) and E (**241**) with IC_50_ values of ~30 nM, against the murine L1210 leukemia cell line [220]. On the other hand, dihydrohalichondramide (**63**) and 33-methylhalichondramide (**242**) inhibit growth in the murine L1210 leukemia cell line with IC_50_ values of ~40 and ~60 nM, respectively, in this case by disrupting actin microfilaments [220,550]. Halichondramide (**244**) presents also a wide array of bioactivities, including cytotoxic and cytostatic activities and antiproliferative and antimigratory effects in vitro, as reported elsewhere [479,551,552]. Most of these compounds probably originate from a sponge diet [218]. A mechanism of detoxification has been suggested for the slug to deal with halichondramide and transform it into less toxic compounds [218,221].

Another interesting species is *Jorunna funebris*, which in India presents the cytotoxic isoquinoline alkaloid jorumycin (**189**) [166,553,554]. Jorumycin (**189**) is cytotoxic at very low concentrations, with an IC_50_ of 12.5 ng/mL against some cancer cell lines, such as P388, A549, HT29, and MEL28, including cells resistant to apopototic stimuli, and having a saframycin-like structure similar to ecteinascidin 743, one of the most active marine-derived antitumor agents isolated from the tunicate *Ecteinascidia turbinata* and an approved drug already on the market [43,553,555,556,557]. A synthetic compound derived from jorumycin (**189**) is in phase II clinical studies for endometrial and cervical cancer, as well as solid human tumors and hematological diseases (Ewing sarcoma, urothelial carcinoma, and multiple myeloma) [556,558,559]. Specimens from Thailand, instead, present jorunnamycins A–C (**245**), along with renieramycins (**246**), which are also cytotoxic, while specimens from Sri Lanka presented several isoquinoline–quinone metabolites from the sponge *Xestospongia* sp. [22,560]. Jorunnamycins A–C (**245**) are reported to be obtained after treatment of the samples with potassium cyanide, yielding more stable compounds while still conserving high cytotoxicity against HTCLs [560]. Jorunnamycin C (**245**) and renieramycin M (**246**) present IC_50_ values at low nanomolar ranges against human colon (HCT-116) and breast (MDA-MB-435) cancer cells [561]. Their mechanism of action has been reported as the downregulation of protein tyrosine phosphatase receptor type K (PTPRK) in vitro, since PTPRK is a tumor suppressor gene product that may be involved in colon cancer [516,561,562,563]. Some structurally related compounds were also found in specimens from South China, such as the fennebricins A and B, and other molecules, probably also from a *Xestospongia* sp. [564,565].

The Mediterranean *Peltodoris atromaculata*, and the sponges on which it feeds on, *Petrosia ficiformis* and *Haliclona fulva*, contain cytotoxic long-chain fulvinol-like polyacetylenes, namely, petroformynes (**247**) [566,567,568,569]. These compounds are structurally very similar to the sponge compounds neopetroformynes and are active against murine P388 leukemia cells with IC_50_ values of 0.09–0.45 μM [570]. Further metabolites from this slug include some other polyacetylenes, such as the hydroxy-dehydroisofulvinol (**248**), very similar to fulvinol, which is active against four cell lines, i.e., murine P388 leukemia, A549 NSCLC, HT-29 colon cancer, and SKMEL-28, at IC50 ~2 μM [571]. Hydroxy-dehydroisofulvinol (**248**) presents an IC_50_ value of ~3 μM against the SKMEL28 melanoma cell line [571].

Finally, nembrothids also present some cytoxocity. The alkaloids tambjamines (**65–70**), as mentioned above, are found in several *Tambja* species (*T. capensis*, *T. ceutae, T. eliora*, *T. morosa*, *T. stegosauriformis, T. verconis*) along with their bryozoan prey (*Bugula dentata* or *Sessibugula translucens)* in different geographical localities [21,88,207,223,228,572,573]. *Roboastra* species feeding on *Tambja* species also present tambjamines (**65–70**), and *R. tigris* obtains them from *T. abdere* and *T. eliora* [223]. Tambjamines (**65–70**) have also been found in *Nembrotha* spp. and the tunicate *Atapozoa* sp. [226,227]. Some tambjamines (**65–70**), which are similar to the bacterial compounds prodigiosins, have been described to cause DNA damage and induce apoptosis [223,574,575,576,577]. Tambjamine D (**68**) is active against several tumor cell lines by intercalating into DNA, as well as by promoting single-strand DNA oxidative cleavage, although a lack of selectivity was described [228,578,579,580,581,582,583,584,585,586]. Tambjamine K (**249**) and the tetrapyrrole (**72**) display concentration-dependent cytotoxicity against tumor and non-tumor mammalian cells, with IC_50_ values between ~0.004 and 15 μM and IC_50_ ~19 μM against mouse 3T3-L1 fibroblasts for tambjamine K (**249**) [232,584,587]. In fact, tambjamine K (**249**) is very selective, showing a ~4000-fold differential sensitivity between human Caco-2 colon cancer cells and HeLa cervix cancer cells [229]. Tambjamine C (**67**), instead, is a good transmembrane anion transporter, similar to prodigiosins, which are relevant in cancer cell biology and cancer cell migration, and they are expressed differently in diverse cancer cells [581,582,583,584,585,586]. 

##### Dendronotida

Within this group, several species have been described to present cytotoxic compounds. Punaglandins (**250**) are cytotoxic PGs obtained by *Tritonia* sp. From its octocoral prey, *Telesto riisei,* active at an IC_50_ of 0.03 µM to mouse leukemia cells [587]. Dotofide (**251**) is found in *Doto pinnatifida* and is active against several cell lines using the MTT colorimetric assay [505]. Against human glioma, dotofide (**251**) shows a GI_50_ value of 18.1 µM for Hs683 oligodendroglioma (ATCC HTB-138), and of 28.8 µM for U373 glioblastoma (ECACC 08061901) [505]. For human carcinoma, dotofide (**251**) displays GI_50_ values of 29.4 µM for A549 NSCLC (DSMZ ACC107), and 28.1 µM for MCF-7 breast carcinoma (DSMZ ACC115) [505]. Finally, for melanoma, it displays GI_50_ values of 60.5 µM for human SKMEL-28 (ATCC HTB-72) and 9.6 µM for mouse B16F10 (ATCC CRL-6475) [505].

The sesquiterpenes tritoniopsins A–D (**75–78**) are found in *Tritoniopsis elegans* and its soft coral prey *Cladiella krempfi*, where the slug accumulates tritoniopsin A (**75**) in its mantle at higher concentrations than the coral or other slug tissues [238]. Rat cell lines were used to test the cytotoxicity of tritoniopsin A (**75**), resulting in a weak to moderated activity [234].

##### Euarminida

The previously mentioned eunicellin diterpenes (**187**) from *Dermatobranchus ornatus* display moderate cytotoxicity to A-549, SKOV-3, SK-MEL-2, and HCT-15, along with inhibition of cell division in fertilized starfish eggs [22]. The South African slug *Leminda millecra* possesses sesquiterpenes and prenylquinones from its diet of octocorals, mainly of the genus *Alcyonium* and gorgonians such as *Leptogorgia palma* [588,589,590]. Among them, a prenylated hydroquinone (**252**) possesses moderate inhibitory activity, with values of GI_50_ around 6–9 μM against WHCO1 and WHCO6 esophageal cancer cell lines, inducing apoptosis via generation of reactive oxygen moieties [589,590].

##### Aeolidida

*Hermissenda crassicornis* presents L-6-bromohypaphorine (**253**), reported to be an agonist of human a7 nicotinic acetylcholine receptor [591]. In *Phyllodesmium briareum*, brianthein W (**254**) is reported to have an ED_50_ of 0.76 μg/mL against P-388, while excavatolide C (**255**) displays an ED_50_ of 0.3μg/mL for P-388, and an ED_50_ of 1.9μg/mL for KB, A-549, HT-29 [248]. Both compounds were traced to the diet the octocoral *Briareum* sp. [248]. Similarly, *P. magnum* possesses 11-*epi*sinulariolide acetate (**256**) with an ED_50_ of 1.2μg/mL for P-388, ED_50_ of 1.9μg/mL for HT-29, and ED_50_ of 0.8μg/mL for HL-60 [248]. Moreover, the previously mentioned diterpene trocheliophorol (**84**) from *Phyllodesmium longicirrum* has been reported as cytotoxic [245]. 

Phidianidines A (**257**) and B are bromoindole alkaloids from the Chinese slug *Phidiana militaris* [361,592]. Phidianidines (**257**) are the only known marine source of the 1,2,4-oxadiazole system, and their interesting structure promoted their synthesis, as well as that of several analogues [593,594,595,596,597,598,599,600,601,602,603,604,605]. Phidianidines (**257**) show cytotoxicity against several cell lines, such as C6 and HeLa tumor cells at nanomolar concentrations [592]. Their IC_50_ values stand from ~0.4 to >100 μM in three cancer cell lines, with no selectivity against mouse 3T3-L1 fibroblasts and rat H9c2 cardiomyocytes (IC_50_: ~0.1 and ~5 μM) [592,606]. Human HeLa cervix cancer cells are very sensitive to the growth inhibitory effects of phidianidines (**257**), contrarily to Caco-2 colon cancer cells [606]. Phidianidines (**257**) are also selective and potent ligands with partial agonist activity against the μ opiod receptor (when compared to δ- and κ-opiod receptors), which is involved in cancer progression [593,607]. Virtual screening allowed for the identification of phidianidine A (**257**) as a potential ligand for CXCR4, a chemokine receptor involved in several diseases, including cancer progression, metastasis, and immunodeficiency disorders, and competing with natural ligand CXCL12 as observed by molecular docking, proving that it is a CXCR4 antagonist [608]. Moreover, phidianidine A (**257**) significantly reduced the CXCL12-induced migration at 50 μM in a rat cell line of pituitary adenoma [609].

#### 3.1.2. Pleurobranchoidea 

*Pleurobranchus albiguttatus* and *P. forskalii* from the Philippines contain the cytotoxic chlorinated diterpenes chlorolissoclimide (**258**), dichlorolissoclimide (**259**), haterumaimides (**260**), and 3β-hydroxychlorolissoclimide (**261**), which are acquired from its tunicate prey *Lissoclinum* [610]. These were tested against the NCI panel of 60 tumor cell lines [610]. In Indonesia, *P. forskalii* presents a cyclic hexapeptide instead, keenamide A (**262**), containing a thiazoline and an isoprene residue, which has been reported active against four cancer cell lines (P388, A549, MEL20, and HT29) with IC_50_ values of 2.5–8 μM [611,612]. In Japan, *P. forskalii* contains a macrocyclic dodecapeptide, cycloforskamide (**263**), contaning three thiazoline heterocycles [612]. Cycloforskamide (**263**) displays an IC_50_ value of 5.8 μM against murine P388 leukemia cells [387]. Unfortunately, the low sensitivity of this model resulted in the NCI not pursuing this compound further [613,614]. In Japan, *P. forskalii* also presented ergosinine (**264**), an alkaloid only described previously in terrestrial higher plants and fungi, which is a known activator of caspase-3 [615,616]. The tunicate-derived compounds chlorolissoclimide (**258**) and dichlorolissoclimide (**259**) have been further tested, resulting in IC_50_ values of 0.7 and 1.25 μM against the NCI 60 cell lines panel, respectively, and also displaying selectivity toward melanoma cell lines [610,613,617,618]. Furthermore, both compounds (**258,259**) inhibit growth in the Corbett–Valeriote soft agar disk diffusion test, but with no selectivity for solid tumors, while the related 3β-hydroxychlorolissoclimide (**261**) presents some selectivity for solid tumors in the same test [610]. In particular, chlorolissoclimide (**258**) and dichlorolissoclimide (**259**) were active against murine P388 leukemia cells resistant to adriamycin by blocking the translation elongation via inhibition of translocation, thus producing an accumulation of ribosomes on mRNA [610,618,619]. On the other hand, haterumaimides A–Q (**260,265–269**) also present interesting activities, as do their synthetic derivatives, against the NCI 60 cell lines panel, with IC_50_ values of 0.08–1 μM for haterumaimide D, and from 0.5 nM to >20 μM for haterumaimides J (**265**) and K (**266**) and haterumaimides C (**267**), G (**268**), and I (**269**), respectively [610,617,620,621,622,623,624].

#### 3.1.3. Tylodinoidea

As previously mentioned, *Tylodina perversa* obtains brominated isoxazoline alkaloids from sponges of the genus *Aplysina* [257,625]. Among them, *iso*-fistularin-3 (**270**) presents a IC_50_ value of ~9 μM against human HeLa cervix carcinoma cells [625].

#### 3.1.4. Cephalaspidea

The most active species in this group is *Philinopsis speciosa*. Because its molecules are structurally similar to cyanobacterial compounds, it has been hypothesized that *P. speciosa* obtains them through its diet of the sea hares *Stylocheilus longicauda* and *Dolabella auricularia,* which in turn feed on cyanobacteria [276]. In Hawai’i, *P. speciosa* presents the cyclodepsipeptide kulolide-1 (**271**), which displays potent antitumoral activity against L-1210 leukemia and P388 murine leukemia cell lines, with IC_50_ values of 0.7 and 2.1 μg/mL, respectively, although these assays are highly sensitive to proapoptotic stimuli [277,614]. Kulolide-1 (**271**) also produced morphological changes in rat 3Y1 fibroblast cells at 50 μM [277]. Further chemical analysis of the species yielded some more related peptides, such as kulolide-2 (**272**), kulolide-3 (**273**), kulokainalide-1 (**274**) and the bidepsipeptides kulokekahilide-1 (**275**) and kulokekahilide-2 (**276**) [276,626,627]. Kulokainalide-1 (**274**) and kulokekahilide-1 (**275**) are reported to be moderately cytotoxic, while kulokekahilide-2 (**276**) is highly effective. Kulokekahilide-1 (**275**) shows growth inhibitory effects with an IC_50_ of ~2 μM in murine P388 leukemia cells, while kulokekahilide-2 (**276**) presents a higher potency with an IC_50_ of 4.2 nM [626,627]. Kulokekahilide-2 (**276**) also displays activity against human SK-OV-3 ovarian and tMDA-MB-435 breast cancer cell lines, with IC_50_ values of 7.5 and 14.6 nM, respectively [627]. For A-10 (non-transformed rat (*Rattus norvegicus*) aortic cells), kulokekahilide-2 (**276**) shows an IC_50_ of 59.1 nM, acting selectively [627]. In other cancer cell lines, namely, human A549 NSCLC, K562 chronic myelogenous leukemia, and MCF-7 breast cancer, kulokekahilide-2 (**276**) shows IC_50_ values of ~0.2 nM [628]. In a methodologically different study, the IC_50_ values for kulokekahilide-2 (**276**) were not the same, with ~19 and ~4 nM for murine P388 leukemia and human HeLa cervix carcinoma cells, respectively [629]. Some very potent analogues have also been synthesized, with IC_50_ values of ~0.001 and ~0.008 nM, respectively, allowing the authors to ascertain the best substituents for cytotoxic activity [628].

Moreover, several polyunsaturated fatty acids with cytotoxic activity against a range of human cancer cells lines, including HT-29, MCF7, and A2058, were described from Arctic specimens of *Scaphander lignarius* [630].

#### 3.1.5. Anaspidea

Sea hares have been proven to contain many interesting cytotoxic compounds, many of them derived from the algae or the cyanobacteria that they feed on [1,2]. Within the genus *Aplysia*, many species have been studied, including *A. angasi, A. dactylomela, A. depilans, A. fasciata, A. juliana, A. kurodai, A. oculifera*, and *A. punctata* [1,2]. *A. angasi* (also reported as *A. dactylomela*), which showed growth inhibitory activity against murine lymphocytic leukemia P338 cells due to the presence of the brominated tricyclic 6-7-5-fused sesquiterpene, aplysistatin (**277**) [631]. Aplysistatin (**277**) showed an IC_50_ value of ~8 μM for P388 leukemia cells and human KB cancer cells [613,631].

*A. dactylomela* presents a wide variety of metabolites [631,632,633,634]. Some dietary halogenated chamigrene sesquiterpenes were described in specimens from the Canary Islands, such as elatol (**278**) and obtusol (**279**), previously found in the red algae *Laurencia elata* and *L. microcladia,* while *iso*-obtusol (**280**) was found in *L. obtusa* [631,632,633,634]. Elatol (**278**) is a potent, non-selective, cytotoxic natural product, with IC_50_ values between 1–10 μM against ten cancer cell lines in several studies [631,635,636]. Remarkably, elatol (**278**) is proapoptotic in murine B16F10 melanoma cells by decreasing Bcl-x and increasing Bak, caspase-9 and p53 expression, although it is known that B16F10 melanoma cells are very sensitive to these stimuli [636,637,638]. Obtusol (**279**) and *iso*-obtusol (**280**) show a much weaker effect inhibiting growth in vitro [631,633,635]. A linear halogenated monoterpene (**281**) was also found, showing growth inhibitory effects towards HM02 (gastric carcinoma), HEP-G2 (liver carcinoma), and MCF-7 (breast carcinoma) cancer cells with IC_50_ values of ~1 μM [631]. Some tricyclic monobromoditerpenes were also found in *A. dactylomela* specimens, such as parguerol (**282**), parguerol-16-acetate (**283**), *iso*-parguerol (**284**), *iso*-parguerol-16-acetate (**285**), and deoxyparguerol (**286**), which were also present in red algae, such as *Jania rubens* [639,640]. In Bahamas and Puerto Rico, however, specimens of this sea hare contain different chemistry [639]. Parguerol (**282**) and the previously mentioned related compounds present in vitro growth inhibitory effects at low micromolar values on murine P388 leukemia, are highly sensitive to proapoptotic stimuli as previously discussed, and are also significantly sensitive to Ehrlich ascite carcinoma [614,639,640]. These activities were more potent with *iso*-parguerol (**284**) derivatives [639,640]. In the Caribbean, some other compounds are present, such as the bromotriterpene polyether aplysqualenol A (**287**), which displays potent IC_50_ values in the NCI assay (60 cancer cell lines panel), particularly an IC_50_ of ~0.4 μM in human SNB-19 central nervous system cancer and T-47D breast cancer cells [641]. Aplysqualenol A (**287**) has been described to be a ligand for DYNLL1 (light chain of dynein type 1), also indicating some anticancer potential [642,643,644,645]. In China, specimens of *A. dactylomela* also present a brominated triterpene polyether, thyrsiferol (**288**), previously reported in the red algae *Laurencia thyrsifera* [646,647]. Thyrsiferol (**288**) has been reported to display a strong growth inhibitory in vitro activity against mouse P388 leukemia cells, and only moderate activity in solid tumors cell lines, with IC_50_ values of ~0.02 and ~17 μM for P388 and A549 NSCLC cancer cell lines, respectively [614,648,649]. Apparently, thyrsiferol (**288**) inhibits hypoxia-induced HIF-1 activation in T47D human breast tumor cells and suppresses hypoxic induction of HIF-1 target genes (VEGF and GLUT-1) at the mRNA level, also suppressing mitochondrial respiration in complex I [648].

*A. depilans* presents an endoperoxide sterol (**289**) with an IC_50_ value of 2.5 μM for human HCT-116 colorectal cancer cells [606,650,651]. *A. fasciata* from Spain presents a degraded sterol, 3-*epi*-aplykurodinone B (**145**) with an IC_50_ value of ~8 μM against mouse P388 leukemia, human A549 NSCLC, HT-29 colon cancer, and SKMEl-28 melanoma [397]. *A. juliana* contains two chlorophyll derivatives, pyropheophorbides a and b, and a halogenated diterpenoid lactone, derived from its diet of green algae, as well as a cytotoxic peptide, julianin-S, which is secreted within its purple ink [288,432,433,434].

*A. kurodai* has been widely studied over the years, containing a series of compounds that include polyketide macrolides, halogenated and brominated mono- and di-terpenes, brominated sesquiterpenoids, sterols, alkaloids, peptides, and others [1,2]. Aplyronines A–H (**191,290–293**) are polyketide macrolides found in Pacific specimens along with aplaminal (**294**), some of them being tested in antitumor clinical trials [46,652,653,654,655,656]. For human HeLa-S3 cancer cells, IC_50_ values were 0.5 nM for aplyronine A (**191**), 3 nM for aplyronine B (**290**), 22 nM for aplyronine C (**291**), 0.08 nM for aplyronine D (**292**), and 10 nM for aplyronine H (**293**) [653,657,658]. However, in a different study with the same cell line, IC_50_ values were ~0.4 nM for aplyronine A (**191**), ~4 nM for aplyronine B (**290**), and ~20 nM for aplyronine C (**291**) [652]. It has been suggested that the methylated amino acids (*N*,*N*,O-trimethyl-serine or *N*,*N*-dimethyl-alanine) in position 22 are important for the inhibition activity of these compounds [653]. Aplyronine A (**191**) is registered at the NCI database as NSC687160, with a mean IC_50_ value of ~0.2 nM in the 60 cancer cell lines, but being selective against some cell lines and most active against MDR cancer cell line NCI/ADR-RES, with an IC_50_ value of ~0.2 nM, as well as for P388 murine leukemia, Lewis murine lung carcinoma, Erhlich murine carcinoma, colon 26 murine carcinoma, and B16 murine melanoma, but very moderated against HOP-92 (NSCLC), OVCAR-4 (ovarian cancer), TK-10 and UO-31 (renal cancers), and BT-549 and T47-D (breast cancers) [652]. Aplyronine A (**191**) also presents proapoptotic effects in cancer cells [46]. Aplyronine A (**191**) was in fact suggested to inhibit the actin microfilaments, since it can depolymerize F-actin and inhibit actin polymerization, forming a complex with monomeric actin (1:1), in a similar way to the well-known tubulin inhibitors vincristine and vinblastine currently employed in some cancer treatments [479,659,660]. Later, other studies suggested that aplyronine A (**191**) forms a 1:1:1 heterotrimeric complex with actin and tubulin, and this is what actually inhibits tubulin polymerization; thus, synthesis is being carried out [661,662,663,664]. Furthermore, an analogue of aplyronine D (**292**) is being analyzed as an antibody–drug conjugate (ADC) and for bearing a linker suitable for bioconjugation [665]. Overall, aplyronines (**191,290–293**) are extremely toxic molecules with huge potential as leads, but they are not yet known to be attached to a suitable mAb [666]. Aplaminal (**294**) is a triazabicyclo-[3.2.1]-octane, displaying an IC_50_ value of ~2 μM against human HeLa S3 cervix carcinoma cells [654]. 

In Japan, *A. kurodai* presents aplaminone (**295**), neoaplaminone (**296**), and neoaplaminone sulfate (**297**), formed from a bromine-containing dopamine and a sesquiterpenoid [667]. Both aplaminone (**295**) and neoaplaminone sulfate (**297**) show IC_50_ values of ~1 μM for human HeLa cervix cancer cells, while neoaplaminone (**296**) is active at IC_50_ ~1 nM [667]. In Japan, *A. kurodai* specimens also contain aplysiaterpenoid A (**298**) and aplysiapyranoids A–D (**299**), displaying moderate cytotoxicities against Vero, MDCK, and B16 cell lines (IC_50_ 19–96 μg/mL) [668,669]. Moreover, mono- and di-terpenes, such as kurodainol (**300**), aplysiaterpenoids A–D (**298**), aplysin-20 (**301**), *iso*-aplysin-20, aplysiadiol (**302**), *epi*-aplysin-20, and *ent*-isoconcinndiol (**303**), are found in *A. kurodai* specimens and are suggested to originate from isoconncindiol of the red algae *Laurencia snyderae* [668,669,670,671,672,673,674]. *A. kurodai* also contains other compounds, including aplydilactone, a dieicosanoid lactone, aplysepsine, and a 1,4-benzoidiasepine alkaloid [675,676]. The egg masses and albumen gland moreover contain cytotoxic peptides, such as aplysianin A or aplysianin E, the latter highly tumor-lytic at 10 ng/mL against MM 46 and MM 48 mice tumor cells [294,677,678,679].

Furthermore, *A. kurodai* presents some brominated sesquiterpenoids, such as (-)-aplysin (**304**), aplysinol (**305**), and aplykurodin A (**306**) and B (**146**) [398,680]. (-)-Aplysin (**304**), originating from its diet on algae, was in fact one of the first halogenated compounds found in marine organisms, and it shows IC_50_ values of 4–8 μM for several cancer cells, with a mean IC_50_ value of ~30 μM at the NCI 60 cell line panel and no selectivity [680,681]. (-)-Aplysin (**304**) was active against the human A549 NSCLC xenograft with 18% of tumor growth reduction in vivo as compared to the control, supposedly by acting as a sensitizer for tumor necrosis factor-related apoptosis, producing TRAIL-induced apoptosis in cancer cells via the P38 MAPK/survivin pathway [682]. (-)-Aplysin (**304**) is also effective against human glioma cells by increasing miR-181 expression, sensitizing the cytotoxic effects of the alkylating drug temozolomide, and inducing cell cycle arrest and apoptosis through the inhibition of the PI3K/Akt signaling, which is relevant in the survival of glioma cells [683]. 

In Egypt, *A. oculifera* contains two halogenated sesquiterpenes, oculiferane (**307**) and *epi*-obtusane (**308**), which displayed IC_50_ values between 2 and 8 μM against human PC-3 prostate cancer, A549 NSCLC, MCF-7 breast cancer, HepG2 liver cancer, and HCT116 colon cancer [684]. *A. punctata* from Spanish coasts present atypical acetates of halogenated monoterpenes, among which three compounds (**309–311**) show IC_50_ values between 1.5 and 2.5 μM against P388, HT-29, A-549, and MEL-28 cancer cell lines [685]. 

Another sea hare, *Dolabella auricularia,* is one of the most studied marine invertebrates, with many bioactive natural products, including polyketides, halogenated terpenes, and peptides, along a wide geographical range [1,2]. Several excellent reviews deal with *D. auricularia* most bioactive compounds [46,478,479], and, therefore, only a brief summary will be included here. This sea hare is able to modify dietary molecules from brown and red algae and also to de novo biosynthesize peptides and polypropionates, while it may also contain cyanobacterial metabolites [686]. We will summarize the cytotoxic activities of aurisides A and B (**312,313**), aurilol (**314**), doliculols A and B (**315,316**), dolabellin (**317**), auripyrones A and B (**318,319**), dolabelides A–D (**320–323**), aurilide (**324**), doliculide (**325**), auristatins (**339–344**), and finally, dolastatins (**192,326–338,346–348**). In Japan, among the dietary compounds from red algae, the macrolide glycosides aurisides A and B (**312,313**) and aurilol (**314**), a polyether bromotriterpene, are cytotoxic to HeLa tumor cell line [687,688]. Aurisides A and B (**312,313**) present a carbon backbone 5,7,13-trihydroxy-3,9-dioxoheptadecanoic acid with a bromine-substituted conjugated diene moiety, a cyclic hemiacetal, and a 14-membered lactone [688]. Auriside A (**312**) displays IC_50_ values of ~0.2 μM against human HeLa S3 cervix cancer cells, while auriside B (**313**) is less potent (IC_50_ 2 μM), and both are being studied for their synthesis [688,689,690]. Aurilol (**314**) presents IC_50_ values of ~7 μM against human HeLa S3 cancer cells, and it is structurally similar to enshuol, a bromo triterpenic polyether with a dioxabicyclo-(5.4.0)-undecane ring system described in *Laurencia*, thus supporting a dietary origin in the slug [687]. Doliculols A and B (**315,316**), non-halogenated acetylenic cyclic ethers similar to *Laurencia* ethers, are moderately cytotoxic macrolides [691,692,693,694]. Dolabellin (**317**), a bisthiazole metabolite found in Indian specimens, showed IC_50_ values of ~10 μM against human HeLa S3 cervix carcinoma cells [695]. 

In *D. auricularia* from Japan, auripyrones A and B (**318,319**) were described to have IC_50_ values of ~0.5 μM against human HeLa S3 carcinoma cells and are being synthesized [696,697,698,699,700]. Furthermore, Japanese specimens contain the macrolides dolabelides A–D (**320–323**), displaying IC_50_ values of ~8 and ~2 μM, for dolabelides A and B (**320,321**) respectively, while dolabelides C and D (**322,323**) showed both values of ~2 μM against human HeLa S3 cervix cancer cells, and synthesis studies are being carried out [692,693,701,702]. 

The cyclic depsipeptide aurilide (**324**) was found in Japanese *D. auricularia* specimens, while aurilides B and C were found in the cyanobacterium *Lyngbya majuscula* from Papua New Guinea [703,704,705]. Aurilide (**324**) displayed an IC_50_ mean value of ~0.01 μM in the NCI 60 cell line panel and was selective for renal, ovarian, and prostate cancer cell lines. It was active in the in vivo NCI hollow fiber assay, but inactive due to high toxicity in a xenograft model [705,706]. The mechanism of action of aurilide (**324**) seems to involve microtubule stabilization, since it does not interact with tubulin, thus being different from taxol [705]. Furthermore, aurilide (**324**) has been reported to selectively bind to prohibitin 1 (PHB1) in the mitochondria, activating the proteolytic processing of optic atrophy 1 (OPA1) and resulting in mitochondria-induced apoptosis [707]. 

Doliculide (**325**) is a mixed peptide–polyketide-originated compound found in Japanese specimens of *D. auricularia*, possessing an iodo-N-Me-tyrosine and a glycine, which inhibits growth of human HeLa S3 cervix carcinoma cells (IC_50_ ~2 nM) [708]. Its action mechanism consists of binding to actin and stopping cancer cells at the G2/M phase of the cell cycle, thus interfering in normal actin assemblage and producing the hyperassemblage of purified actin in the form of F-actin [709]. Synthesis and computational studies are being developed [710,711,712]. Doliculide (**325**) inhibits proliferation and impairs the migratory potential of human MCF-7 and MDA-MB-231 breast cancer cells, while modifying senescence-related genes at non-toxic concentrations in p53 wild-type cancer cells by up to 13% [713].

The most famous compounds of all heterobranchs are the diverse cytotoxic linear and cyclic peptides of *D. auricularia*, the dolastatins (**192,326**–**338,346**–**348**), which were probably used by the Romans [46,197]. Dolastatins (**192,326**–**338,346**–**348**) include many different active molecules, including linear and cyclic peptides, depsipeptides, peptides containing thiazole and oxazole heterocycles, and macrolides [479]. Dolastatins (**192,326**–**338,346**–**348**) are found in small amounts in sea hares and are suggested to originate from their diet, particularly from cyanobacteria of the genera *Symploca, Caldora* and *Lyngbya* [714,715,716]. Among the most known dolastatins, dolastatin 3 (**192**) and dolastatins 10–15 (**326**–**331**) display mild to strong biological activities and were further studied, such as the macrocyclic lactone dolastatin 19 (**332**); dolastatins C (**333**), D (**334**), H (**335**) and *iso*-dolastatin H (**336**); dolastatin G (**337**); and *nor*-dolastatin G (**338**) [694,717,718,719,720,721,722,723,724]. Dolastatin G (**337**) and *nor*-dolastatin G (**338**) show moderate cytotoxicity and are analogs of lyngbyastatin 2 and *nor*-lyngbyastatin 2 described in the cyanobacterium *Lyngbya majuscula* from Guam [725]. Dolastatin 19 (**332**) is structurally similar to the previously mentioned aurisides A (**312**) and B (**313**). In general, these compounds did not pass phase II trials alone, and several studies are being developed to use them in combination with other structures [478,479,726]. 

Dolastatin 3 (**192**), a cyclic peptide with two thiazole rings, was also found in *D. auricularia* from Japan, and further synthesized, showing an IC_50_ < 1 μM in P388 murine leukemia cells [718,727]. Dolastatin 3 (**192**) was also found in *Lyngbya majuscula* from Palau [728]. Dolastatin 3 (**192**) induces a 78% life extension in vivo in the murine P388 lymphocytic leukemia model, and a 52% life extension in murine colon carcinoma 38 [728].

Dolastatin 10 (**326**) is a linear pentapeptide with four unique residues described in 1987 from *D. auricularia* collected at the Indian Ocean, and later found in the cyanobacterium *Symploca hydnoides* together with its methyl derivative, symplostatin 1, and has been often reviewed in the literature [478,714,726,729,730,731]. Dolastatin 10 (**326**) was tested in phase I trials by the NCI but failed later in phase II for advanced and metastatic soft tissue sarcoma, advanced hepatobiliary cancers, pancreatic cancers, and others because of its side effects [478,479,726,731,732]. However, in 2011, brentuximab vedotin (Adcetris^®^), an antibody-dolastatin 10 conjugate (ADC), was approved by the FDA (Food and Drugs Administration) for the treatment of Hodgkin’s lymphoma [43]. This ADC is composed of a highly toxic “warhead” derived from dolastatin 10 (**326**) which is attached to a specific targeting moiety, a monoclonal antibody (mAb) directed to a particular epitope on the cancer cell [726]. Dolastatin 10 (**326**) inhibits microtubule assembly, causing cells to accumulate in metaphase, but it produced bone marrow toxicity in initial trials, local irritation at the injection site, and some mild peripheral neuropathy [733,734]. Dolastatin 10 (**326**) exhibits cytotoxic effects against human lung and breast cancer cell lines via both Bcl-2 phosphorylation and caspase-3 protein activation, and it modulates p53 oncoproteins in human diffuse large-cell lymphoma [735,736]. Other studies describe dolastatin 10 (**326**) activity against ovarian carcinoma xenografts as well as mouse P388 and L1210 leukemia, B16 melanoma, M5076 sarcoma, human LOX melanoma, and MX-1 breast cancer xenografts [736,737,738]. Dolastatin 10 (**326**) is not only an inhibitor of tubulin polymerization, it also inhibits tubulin-dependent GTP hydrolysis as well as the binding of vinblastine, maytansine, and vincristine to tubulin, although the binding site on tubulin is not the same as that of the vinca alkaloids [733,739].

Auristatins (**339–344**) are peptides related to dolastatin 10 (**326**) (see below), approved by the FDA as microtubule-destabilizing agents (MDA), and used as antibody–drug conjugates (ADCs) [740,741,742,743]. Auristatins (**339**–**344**) were synthesized by Pettit’s group in the 1990s while working on dolastatins, and they included auristatin E (**339**), auristatin PHE (**340**), auristatin PYE (**341**), and two aminoquinoline derivatives, auristatin -2-AQ (**342**) and auristatin-6-AQ (**343**), all active against tumor lines (between 10–100 pM) [32,744,745,746]. Several derivatives of auristatins are (or are expected to be soon) in preclinical trials as “ADC warheads”, some reaching phase I, such as DZ-2384 [478,726]. Auristatin PE (**344**), also named soblidotin, TZT-1027, or YHI-501, is a microtubule active drug that exerts a considerable antivascular effect along with a potent cytotoxic effect in several models, including murine P388 leukemia, colon 26 cancer, Lewis lung carcinoma, B16 melanoma, and M5076 sarcoma, as well as human MX-1 breast cancer and LX-1 and SBC-3 SCLC xenografts [747,748]. Auristatin PE (**344**) entered clinical trials for advanced and metastatic soft tissue sarcomas, NSCLCs, and others, but did not proceeded beyond phase II due to toxicity and/or a lack of efficacy in the trials [726]. More than 30 ADCs in clinical trials are based on auristatins [43,555,717,749].

Several excellent reviews, such as those of Newman, have dealt with dolastatin-10 (**326**) and all its derivatives, their evolution as ADC warheads, the auristatin-based ADCs, and the approved and/or tested drugs, such as brentuximab vedotin, polatuzumab vedotin, enfortumab vedotin, ladiratuzumab vedotin, lifastuzumab vedotin, PSMA-ADC, RC-48, telisotuzumab vedotin, tisotumab vedotin, BA-3021, CX-2029, HuMax-AXL-107 (enapotamab vedotin), pinatuzumab vedotin, ABBV-085, AGS67E, ALT-P7, CDX-014, losatuxizumab vedotin, SGN-CD48A, rituximab-MC-vc-MMAE (TRS-005), GM-103, HT-1511, OBI-999, depatuxizumab mafadotin, AGS-16C3F, GSK-2857916 (belantamab mafodotin), W-0101, cofetuzumab pelidotin, NG-HER2 ADC, XMT-1536, ASN-004, ARX-788, lupartumab amadotin, AGS-62P1, and ZW-49, among others [478,666,726]. We strongly recommend these reviews for further details on the different status of the many derivatives that are currently being tested and those that are in clinical trials. 

Dolastatin 11 (**327**), 12 (**328**), 13 (**329**), and 14 (**330**) are further depsipeptides isolated from *D. auricularia*, while dolastatin 12 (**328**) was also reported in *Lyngbya majuscula*–*Schizothrix calcicola* cyanobacterial assemblages [750,751,752,753]. In the NCI cell line panel, dolastatin 11 (**327**) showed an IC_50_ mean value of ~0.07 μM, while dolastatin 12 (**328**) displayed different IC_50_ values in several assays, such as 1 nM for human NCI-H460 NSCLC, 30 nM for human SF-295 CNS cancer, ~0.1 μM for mouse neuro-2a neuroblastoma and 1 μM for P388 leukemia cells [750,754,755]. Dolastatin 11 (**327**) produces a massive rearrangement of the actin filament network in cells, inducing a cytoplasmic retraction and further cell division arrest at the level of cytokinesis [239,241]. Dolastatin 12 (**328**) also targets actin microfilaments [753]. The values of IC_50_ for dolastatin 13 (**329**) and 14 (**330**) against the murine P388 leukemia cell line are reported to be 14 and 20 nM, respectively [751,752]. 

Dolastatin 15 (**331**) is another linear peptide from *D. auricularia* widely used as a potential warhead, with an IC_50_ of 3−5 nM, and with many derivatives being tested after chemical modifications, for example, replacing the C-terminal (S)-dolapyrrolidinone unit with some diverse amides while maintaining its anti-tubulin activity [756,757,758,759,760,761]. Dolastatin 15 (**331**) also produces microtubule depolymerization in vitro, possibly binding to the vinca domain of tubulin, and it is a classical inducer of apoptosis in cancer cells, acting as a conventional proapoptotic cytotoxic agent [762,763,764]. Dolastatin 15 (**331**) presents an IC_50_ against the NCI panel about ten times higher than that of dolastatin 10 (**326**), that is, 2 vs. 0.2 nM, and it is three to four times more potent than vincristine, a clinically used common antiproliferative agent [765]. Tasidotin (**345**), an analog of dolastatin 15 (**331**) where the carboxyl-terminal ester group is replaced by a tert-butyl amide, is also a proapoptotic cytotoxic compound, tested against many different cancer lines, but it did not go beyond phase II clinical trials because of its lack of efficacy [766,767,768].

Dolastatin 16 (**346**), instead, is a cyclic depsipeptide with two amino acids, dolamethylleuine and dolaphenvaline, described in *D. auricularia* from Papua New Guinea, and found also in *Lyngbya majuscula* from Madagascar and *Symploca* cf. *hydnoides* from Guam [769,770,771]. In the NCI cell line panel, dolastatin 16 (**346**) displayed an IC_50_ mean value of ~0.3 μM [770,772]. Specimens of *D. auricularia* from Papua New Guinea also contained dolastatin 17 (**347**), another cyclodepsipeptide with a novel acetylenic β-amino acid named dolayne (Doy), similar to that of onchidin (**121**), which has submicromolar values of IC_50_ against four cancer cell lines [773,774]. Dolastatin 18 (**348**) was found in *D. auricularia* from the Indian Ocean, contains a thiazole ring, and shows submicromolar values of IC_50_ against mouse P388 lymphocytic leukemia and human NCI-H460 NSCLC cell lines [775]. *D. auricularia* from California contains dolastatin 19 (**332**), a macrocyclic lactone related to the previously mentioned aurisides (**312,313**), showing in vitro growth inhibition for breast MCF-7 and colon KM20L2 cancer cells with IC_50_ values of ~1 μM [776]. Similarly, other dolastatins from specimens from Japan and the Indian Ocean, such as dolastatin D (**334**), dolastatin G (**337**), *nor*-dolastatin G (**338**), dolastatin H (**335**), and *iso*-dolastatin H (**336**), also display activity against some cancer cell lines, such as human HeLa S3 cancer cells [721,723,724].

*Stylocheilus* and *Bursatella* are also cyanobacterial feeders containing interesting cytotoxic compounds [1,2,4]. Aplysiatoxin (**96**) and debromoaplysiatoxin (**97**) were found in *S. longicauda* from Hawai’i, originating from *Lyngbya majuscula* [302,303,777]. The mixture of these two compounds was toxic to mice (LD_100_ 0.3 mg/kg), and both compounds are potent PKC activators that are being tested as anticancer lead structures (along with some derivatives) based on their anti-proliferative activity while removing their tumor-promoting activities [778,779,780]. Further studies on different populations of *S. longicauda* also from Hawai’i reported complex proline esters, makalika (**99**) and makalikone (**100**), together with lyngbyatoxin A (**349**) with antitumor properties, again from a diet of *L. majuscula* [304,305]. Makalikone (**100**) shows moderate activity against P388, A549, and HTB38 cancer cell lines, with IC_50_ values between 2.5 and 5 μg/mL [305]. Lyngbyatoxin A (**349**) is toxic to mice (LD_100_ 0.3 mg/kg), and it has been reported to act as a tumor promoter [781]. Lyngbyatoxin A acetate (**101**), also found in the sea hare and its cyanobacterial prey in Hawai’i, displays very potent toxicity against several cancer cell lines, with IC_50_ values ~0.05 μg/mL [305]. Furthermore, some alkaloids such as malyngamides O (**102**) and P (**103**) were found in *S. longicauda* and *L. majuscula* [307,781,782]. Malyngamide O (**102**) shows IC_50_ values of 2 μg/mL against the cancer cell lines P388, A549, and HT29 [123]. In Guam, *S. longicauda* accumulates malyngamydes and transforms malyngamyde B into an acetate [307]. In fact, more than 30 malyngamides have been isolated from cyanobacteria and sea hares, and they have been observed to be N-substituted amides of long-chain methoxylated fatty acids, which are characterized by presenting a trans double bond and a 7S configuration of the oxygen-bearing carbon [479].

*Bursatella leachii* also feeds on “*Lyngbya*”, accumulating lyngbyatoxin A (**349**) and debromoaplysiatoxin (**97**) in the digestive gland [783]. In New Zealand, *B. leachii* presents an alkaloid derived also from cyanobacteria, malyngamide S (**350**), with cytotoxic properties, while in Thailand, it presents hectochlorin (**351**) and deacetylhectochlorin (**352**), which are also cytotoxic compounds previously isolated from *Lyngbya majuscula* and structurally similar to dolabellin from *Dolabella auricularia* [784,785,786]. Malyngamide S (**350**) and malyngamide X (**353**), both found in *B. leachii,* possess in vitro growth inhibitory properties against several cancer cell lines, with IC_50_ values between ~4 and ~8 μM against murine P388 leukemia and human A549 NSCLC, NCI-H187 (SCLC), HT-29 colon cancer, HL60 leukemia, KB and BC breast cancer lines [307,786,787]. Hectochlorin (**351**) and deacetylhectochlorin (**352**) show growth inhibitory effects in vitro against human KB, NCI-H187 SCLCL, and BC breast cancer cell lines, with deacetylhectochlorin (**352**) displaying a mean IC_50_ value of ~1 μM, and hectochlorin (**351**) a mean IC_50_ value of ~5 μMin the NCI cell panel (being more potent against colon, melanoma, ovarian, and renal cell lines) [784,785]. Additionally, hectochlorin (**351**) seems to be cytostatic rather than cytotoxic in regard to the obtained dose–response curves [785].

#### 3.1.6. Sacoglossa 

Within this group, caulerpenyne (**155**)—a sesquiterpene found in *Elysia* spp. and other species (see above), as well as in its diet of the green algae *Caulerpa*—is active against several cancer cell lines at IC_50_ ~10 μM, while it has an IC_50_ ~40 μM in the NCI panel [316,411,788,789,790]. Caulerpenyne (**155**) is not selective for normal (hamster fibroblasts, human keratinocytes, and melanocytes) and cancer cells [790,791]. Caulerpenyne (**155**) induces tubulin aggregation, inhibiting the polymerization of tubulin and bundling of the residual microtubules, but it does not bind to colchicine, taxol, or vinca-alkaloid binding domains [790,792]. It has been shown that caulerpenyne (**155**) may block the stimulation of mitogen-activated protein kinase (MAPK), thus affecting the control of cell proliferation, differentiation, or death [790,793].

Kahalalides (**194,354–356**) are cyclodepsipeptides found in *Elysia* species (*E. rufescens, E. ornata, and E. grandifolia*) and their algal food, *Bryopsis pennata* [331,794,795,796,797]. Kahalalides (**194,354–356**) include more than 20 structurally diverse molecules, ranging from a C-31 tripeptide to a C-77 tridecapeptide, where each peptide contains a different fatty acid chain [794]. Among them, kahalalide F (**194**), a cyclic peptide connected by an amidic bond to a short fatty acid chain, is the most potent, being reported to show antitumour activity and tested in phase I trials in patients with hormone-refractory prostate cancer [797,798]. Treating cancer cells with kahalalide F (**194**) resulted in critical changes in lysosomal membranes and large vacuoles, producing cell swelling, while it is also reported to display specific interactions with cell membrane proteins [482,799]. Kahalalide F (**194**) inhibits the PI3K–AKT signaling pathway in the breast cancer cell lines SKBR3 and BT474 [800]. The IC_50_ values of kahalalide F (**194**) in the NCI 60 cell line panel were from 0.2 to 10 μM, with hormone-independent prostate cancer cells being the most sensitive [267]. Kahalalide F (**194**) also displays in vivo activity against human prostate hormone-independent xenograft models and in the hollow fiber test [794,801,802,803]. Kahalalide F (**194**) was tested in several oncological clinical trials and was taken to phase II, although it failed to be effective [804,805]. Kahalalide F (**194**) is found in nature as a mixture with *iso*-kahalalide F (**354**), also possessing interesting bioactivities [331,806]. *Iso*-kahalalide F (**354**) also entered phase II clinical trials for liver cancer, melanoma, and NSCLC patients, but was also ineffective [807]. The origin of these compounds has been suggested to be *Mycoplasma* spp. Or *Vibrio* spp. Bacteria, since they are affiliated with *E. rufescens* and its mucus [808]. In *E. ornata* from India, two more compounds, kahalalide Z_1_ (**355**) and kahalalide Z_2_ (**356**), were found, differing from kahalalide F (**194**) in the *N*-terminal acid moiety and some aminoacid units of the peptide chain, and displaying a bioactivity profile comparable with kahalalide F (**194**) [795]. Furthermore, elisidepsin trifluoroacetate (PM02734, IrvalecR), a kahalalide-derived synthetic cyclic depsipeptide (**357**), displays cytotoxic activity, causing cell death by inhibiting the AKT/mTOR pathway [331,809]. Elisidepsin (**357**) also underwent clinical development after showing IC_50_ values between 0.4 and ~9 μM in a 23 cancer cell lines, including breast, colon, head and neck, lung, ovary, pancreas, prostate, and melanoma types [810,811,812]. Elisidepsin (**357**) acts at the cell membrane level, interacting directly with glycosylceramides in the membrane of cancer cells, while inducing necrosis-like cell death in the yeast *Saccharomyces cerevisiae* [813,814,815]. Elisidepsin (**357**) is active in vivo against human melanoma, liver, pancreas, breast, and prostate cancer xenografts [816]. However, in clinical trials, elisidepsin (**357**) has been ineffective to date [811,817].

#### 3.1.7. Pulmonata

*Trimusculus* species present a single type of labdane diterpenoids, such as *T. costatus* and *T. reticulatus* from different geographic localities [333,334,818]. In Chile and South Africa, *T. costatus* and *T. peruvianus* metabolites present cytotoxicity [333,335,336,337]. An atypical C-21 hydroxylated sterol (**358**) from *T. peruvianus* presented IC_50_ values of ~6 μM when tested against human HCT-116 and HT29 colon cancer cells [819]. A secosterol (**359**) from *T. costatus* was also active, with an IC_50_ value of ~3 μM against the WHCO1 esophageal cancer cell line [337]. 

*Siphonaria* species, *S. capensis*, *S. concinna*, *S. cristatus*, and *S. serrata,* contain de novo biosynthesized polypropionates, and some of which also present cytotoxic activity [27,339,340,341,342,343,344,345,346,347,348,349,350,351,352]. In particular, *iso*pectinatone, siphonarienolone (**119**) and others are active at 2.5 μg/mL against P388, A549, HT29, and MEL28, while pectinatone (**166**) and siphonarienfuranone are active at 5 μg/mL, and siphonarienedione, siphonarienone, and *iso*siphonarienolone are active at 10 μg/mL [27,339,340,341,342,343,344,345,346,347,348,349,350,351,352]. Onchidiidids present sesquiterpenoids, depsipeptide acetates, and propionates with 32 carbon atoms, two *γ*-pyrone rings, and a number of hydroxyl groups [12]. *Onchidium* species possess cytotoxic cyclic depsipeptides, such as onchidin (**121**), and the tropical *Onchidium* sp. also possesses cytotoxic acetates and propionates [358,359,820]. Onchidin (**121**) and onchidin B (**360**) are active against murine P388 leukemia and human KB oral cancer cells at IC_50_ values of ~ 7 μM [358,359]. In China, *Onchidium* sp. presents bis-γ-pyrone polypropionates, such as onchidione (**122**) and related compounds, such as onchidiol (**361**) and ilikonapyrones (**362,363**) lacking the hemiketal ring, in different populations [12,357,360,362,821,822,823]. Both kinds of compounds were tested against HCCLs, resulting in 3-acetyl-11-(3-methylbutanoyl)-13-propanoyl-ilikonapyrone (**362**) being active, inhibiting growth in all tested cell lines with IC_50_ between 3 and 9 μM (A549 NSCLC, MCF-7 breast cancer, PC-3 prostate cancer, Hs683 oligodendroglioma, U373 glioblastoma, and SKMEL-28 melanoma), and being comparable to etoposide and camptothecin [357]. This compound (**362**) seems to be active against cancer cells that present resistance to proapoptotic stimuli [357]. Moreover, onchidione (**122**) and the related 3-acetyl-onchidionol and 4-*epi*-onchidione were reported to have significant effects on the splicing of XBP1 mRNA, which is an important regulator of some genes related to the growth of tumors [821].

### 3.2. Antibiotic Activity

Most groups of heterobranchs present compounds with antibiotic activity, the exceptions being dendronotacean and aeolidacean nudibranchs, pleurobranchoideans, cephalaspideans, and pteropods (Figure 23, Table 11) [1,2]. This is an open field for research, since bacterial strains are becoming resistant or multiresistant to known antibiotics, and new molecules are strongly needed to target them [31,830]. 

#### 3.2.1. Nudibranchia

##### Doridacea

Both olepupuane (**14**) and polygodial (**13**), previously mentioned above, are found in different dendrodorid nudibranchs and show antifungal activity against *Saccharomyces cerevisiae* IFO 0203 and *Hansenula anomala* IFO 0136 [81,831]. Moreover, extracts of the egg masses of *Dendrodoris fumata* show antibacterial activity against *Escherichia coli*, *Staphylococcus aureus* and *Pseudomonas aeruginosa*, thus protecting embryos against bacterial infection [832].

Phyllidids, well-studied colorful nudibranchs, often contain isocyanate compounds [1,2]. These isocyanates display a wide array of activities, including antibiotic activity, and have been well studied in recent years [101,102,103,104,105]. One of the best-known species is *Phyllidiella pustulosa,* with compounds obtained from its dietary sponges, mainly *Acanthella cavernosa*, from different geographical localities [119,120,121,122]. *P. pustulosa* from Fiji presents axisonitrile-3 (**25**), displaying strong growth inhibition against *Mycobacterium tuberculosis* (MIC 2 μg/mL) [118,427]. Similarly, *Phyllidia picta* from Bali contains the axane sesquiterpenoids pictaisonitrile-1 (**23**) and pictaisonitrile-2, while *Phyllidia coelestis* and *P. pustulosa* from China, as well as their probable sponge prey *A. cavernosa*, present a nitrogenous cadinane-type sesquiterpenoid, xidaoisocyanate A (**24**), among other terpenoids [112,113,117]. Furthermore, *Phyllidia varicosa* presents two 9-thiocyanatopupukeanane sesquiterpenes (**126**), sequestered from the sponge *Axinyssa aculeata* [110]. Altogether, 9-*iso*cyanopupukeanane (**21**) and *epi*-9-*iso*cyanopupukeanane, as well as their thiocyano derivatives, are moderately antibacterial against *Bacillus subtilis* and antifungal against *Candida albicans,* and they were also further isolated from *Phyllidiella rosans* (*P. bourguini*) [110,373].

*Doriprismatica (Glossodoris) atromarginata* from Australia, Sri Lanka, and India, possesses different compounds from its demosponge preys (*Spongia* (*Hyatella*) sp., *Hyrtios* spp., and *Hyattella cribriformis*), two scalarane sesterterpenes in their MDFs, some pentacyclic scalaranes, and heteronemin (**233**) [22,92,166,176,180,380,381,382]. Heteronemin (**233**), a scalarin-skeleton sesterterpene first reported in the sponge *Heteronema erecta*, displays antibacterial activity towards *M. tuberculosis* H_37_Rv, with a MIC of 6.25 μg/mL [85,180,833]. Other *Glossodoris* species from Australia, *G. hikuerensis* and *G. vespa,* also contain heteronemin (**233**) in their viscera, along with scalaradial (**44**), 12-deacetoxy-12-oxoscalaradial (**43**), and 12-deacetoxy-12-oxo-deoxoscalarin (**136**) in their mantle [178]. In particular, 12-*epi*-scalaradial of *G. hikuerensis* and *G. cincta* shows antimicrobial activity at 10 μg/disk against *S. aureus*, *B. subtilis*, and *C. albicans* [166].

Some compounds described in *Chromodoris willani* collected in Okinawa contain two sesterterpenes, deoximanoalide (**364**) and deoxysecomanoalide (**365**), biotransformed from manoalide and secomanoalide, respectively, possessing antimicrobial activity against *E. coli* and *B. subtilis*, and showing inhibition of snake venom phospholipase A2 at 0.2–0.5 µg [159]. On the other hand, nakafuran-8 (**54**) and nakafuran-9 (**51**), found in several chromodorid species as reported above, were tested against *E. coli*, *S. aureus*, *P. aeriginosa* and *B. subtilis* in a disk diffusion assay, displaying no antibacterial activity [157,165]. In contrast, in *Chromodoris petechialis*, puupehenone (**218**) shows a MIC of 3 μg/mL against *C. albicans* [158].

Hawaiian specimens of the bright red “Spanish dancer” nudibranch, *Hexabranchus sanguineus*, yielded several bioactive macrolides (see above) [222]. Specimens of *H. sanguineus* from Fiji additionally yielded two thiazole cyclic peptides, sanguinamide A (**64**) and B (**366**) [219]. Both sanguinamide A (**64**) and B (**366**) were found at very low concentrations, 0.0023 and 0.011% dry weight, respectively [219]. Sanguinamide B (**366**) showed a moderate antibacterial activity against *P. aeruginosa*, reducing twitching motility [834,835]. *H. sanguineus* specimens from different locations showed different compounds, most of them probably from dietary sponges (*Halichondria, Axinella,* and *Dysidea*) [22,219,545,836,837,838]. In Indonesia, Hawai’i and Japan, *H. sanguineus* presents two trisoxazole macrolides, ulapualide A (**190**) and B (**236**), that inhibit the growth of *C. albicans*, while halichondramides (**244**) and kabiramides A–E (**62,238**–**241,243**) inhibited several fungi [218,222,546,547,836].

The Indian *Jorunna funebris* possesses, among other related metabolites, the isoquinoline alkaloid jorumycin (**189**), which is very similar to renieramycin E from the sponge *Reniera* sp. [166,553,839]. Jorumycin (**189**) presents antimicrobial activity against *Bacillus subtilis* and *Staphylococcus aureus* [557]. Jorumycin (**189**) was found in the mucus secretion, and, thus, a defensive role was proposed for it [166]. *J. funebris* and its prey, *Xestospongia* spp., present several isoquinolinequinones and bistetrahydroisoquinolines, among which some isoquinolinequinones also display antibacterial activity [553,560,564,565,840]. 

Although the metabolites have not yet been described, the organic extract of *Halgerda stricklandi* displays modest activity against *Staphylococcus aureus*, but no activity against a range of several other bacteria and fungi [840]. *Halgerda aurantiomaculata* contains a tryptophane derivative called zooanemonin (**367**), previously isolated from several sponges and the sea anemone *Anemonia sulcata*, which is also reported to show antibacterial activity [841].

Finally, within the Nembrothidae, the previously mentioned alkaloids tambjamines (**65**–**70,249**) also possess antimicrobial activity from their diet [223]. Particularly, their blue tetrapyrrol (**72**), presumably derived from a diet of ascidians, is active against *B. subtilis* at 5 µg/disc [157].

##### Euarminida

Chemical studies of the South African *Leminda millecra* described some sesquiterpenes from dietary origin, including millecrones A and B (**368,369**) and millecrols A and B (**370,371**) [588,589]. Millecrone A (**368**), originating from the soft coral *Alcyonium fauri*, inhibited the growth of *Candida albicans* at 50 µg/disk, while millecrone B (**369**) from the gorgonian *Leptogorgia palma* was active against both *Staphylococcus aureus* and *Bacillus subtilis* at 50 µg/disk [21,589]. In contrast, millecrol B (**371**) was only active against *B. subtilis* at 50 µg/disk [21]. 

Furthermore, *Dermatobranchus otome* from Japan presents the germacrane sesquiterpenoids DO1 (**372**), DO2 (**373**), and DO3 (**374**), displaying antibacterial activity against *B. subtilis* [842]. 

Extracts of *Armina babai* also display antimicrobial activity against *Pseudomonas* sp. and *Proteus mirabilis*, although the compounds have not yet been described [843]. *A. babai* from India possesses a ceramide also found in the gorgonian *Acabaria undulata* [842,844].

#### 3.2.2. Tylodinoidea

As mentioned above, the Australian *Tylodina corticalis* selectively accumulates some bromotyrosine-derived alkaloids from the sponge *Pseudoceratina purpurea*, which contains a larger variety of these compounds [258]. Among them, hexadellin (**375**) and aplysamine 2 (**376**) display mild antibiotic activity against *E. coli* and *S. aureus* at concentrations of 125–250 µg/mL [262]. 

#### 3.2.3. Anaspidea

*Aplysia*, as mentioned above, is one of the most studied genera, with many NPs displaying a wide range of activities around the world [1,2]. In particular, the brominated diterpenes, glandulaurencianols A–C (**162,163**), as well as punctatol (**164**) from *Aplysia punctata*, probably from the red algae *Laurencia glandulifera*, possess the laurencianol skeleton, a known antibacterial diterpene from *Laurencia glandulifera* that is active against *Escherichia coli* and *Bacillus subtilis* [429,430,431]. Moreover, a purple secretion of the sea hare *A. juliana* contains julianin-S, an antibacterial peptide suggested to protect its egg masses from microbial infections, together with some unsaturated fatty acids [288,434]. Similarly, aplysianin E from *A. kurodai* eggs shows antifungal activity against *C. albicans* at IC_50_ >16 µg/mL [672,673,674].

As mentioned above, *Dolabella auricularia*, possesses the glycoprotein dolabellanin A, probably de novo biosynthesized [435]. Besides its antineoplastic activity, dolabellanin A also shows antibacterial activity against *E. coli*, which may protect the egg masses from bacterial pathogens [435]. 

Finally, bursatellin (**105**), a diol nitrile alkaloid found in *Bursatella leachii plei* from Puerto Rico, is structurally related to the well-known antibiotic chloramphenicol [311]. In the Mediterranean, both the + and – isomers of bursatellin (**105**) are found in the external extracts of *B. leachii leachii* and *B. leachii savignyana* [312].

#### 3.2.4. Sacoglossa 

In this group, the previously mentioned cyclodepsipeptides kahalalides A (**377**) and F (**194**) from *Elysia rufescens* and its algal food, *Bryopsis* sp., are active against mycobacteria, with kahalalide A (**377**) inhibiting *M. tuberculosis* H37Rv by 83% at 12.5 μg/mL, and kahalalide F (**194**) by 67% at 12.5 μg/mL [331,411,794,797]. Kahalalide F (**194**) also inhibited *Mycobacterium intracellulare* at a MIC of 25 μg/mL [331]. Kahalalides (**194,354–356**) are also found in *E. rufescens*, *E. ornata* and *E. grandifolia,* and their algal diet *Bryopsis pennata* [794,795], with kahalalide F (**194**) always being the most active compound [794,795]. Kahalalide F (**194**) is found in a mixture together with its isomer *iso*-kahalalide F (**354**), which also shows relevant bioactivities [331,806]. Both compounds have been suggested to originate from bacterial symbionts, although more research is needed to prove this [808].

Chlorodesmin (**114**), from the Australian *Cyerce nigricans*, is a diterpenoid previously known from the green algae *Chlorodesmis fastigiata* with antibacterial and antifungal activity [845,846].

#### 3.2.5. Pulmonata

Several species of *Siphonaria* (*S. capensis*, *S. concinna*, *S. cristatus*, and *S. serrata*) possess different types of polypropionates in their mantle and mucous secretion, affecting gramm + bacteria [338]. Species from Australia, West and East Atlantic, and South Africa displayed antimicrobial activity due to acyclic compounds with a 2-pyrone and furanone rings (type I), such as siphonarienolone (**119**), which were similar to the polypropionates of cephalaspideans [340,341,342,343,344,345,346]. On the other hand, polypropionates with a profuse polyoxygenated network that frequently cyclizes (Type II), such as siphonarin A (**120**), similar to those of actinomycetes, are found in *Siphonaria* species from Australia, New Zealand, North-East Pacific, Pacific Islands, and South Africa [347,348,349,350,351,352]. *S. diemenensis* and *S. pectinata* present diemenensin-A (**165**) and pectinatone (**166**) [340,341,343]. Diemenensin-A (**165**) inhibited *S. aureus* and *B. subtilis* at 1 µg/disc and 5 µg/disc, respectively, while pectinatone (**166**) inhibited *S. aureus, B. subtilus*, *C. albicans*, and *S. cerevisiae* [341,343]. 

### 3.3. Antiparasitic Activity

**Figure 24:**
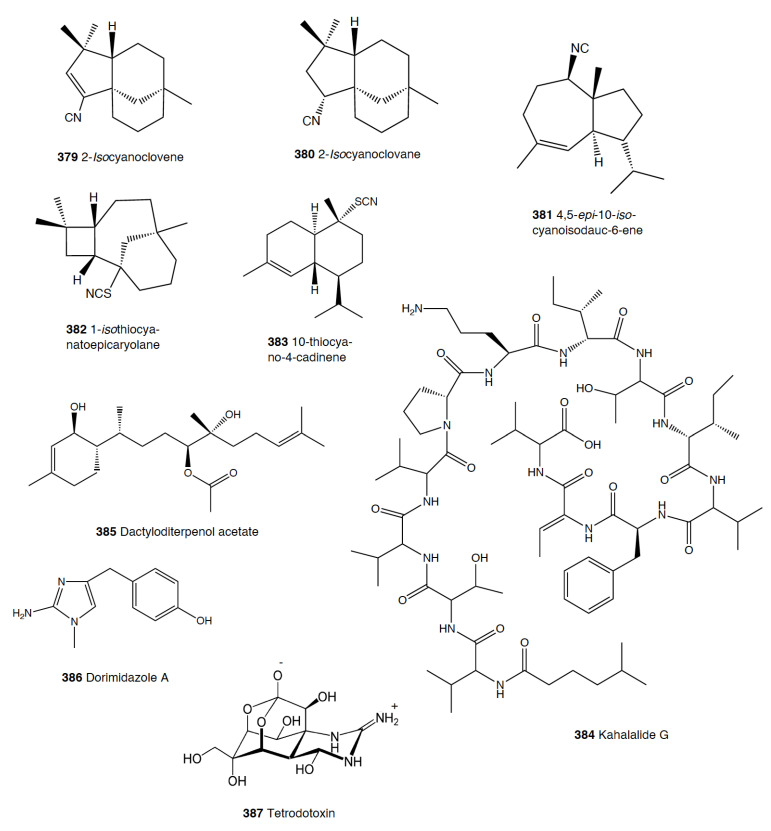
Structures of selected antiparasitic compounds and antivirals from Doridacea and Sacoglossa, anti-inflammatory compounds from Anaspidea, and compounds with other pharmacological activities in heterobranch molluscs. These molecules may also display other activities, as reported in the text.

Currently, another important need is to identify antiparasitic compounds, although the antiparasitic activities included here mainly comprise compounds related to antiplasmodial effects. Within heterobranchs, antimalarial compounds have been described in several doridacean nudibranchs, while only one species of sacoglossa has been cited to possess antileishmanial activity [1,2] (Figure 23 and Figure 24, Table 12).

#### 3.3.1. Nudibranchia

##### Doridacea

The doridacean nudibranch *Notodoris gardineri* from the Philippines presents the imidazole alkaloids *iso*-naamidine-A (**160**) and dorimidazole-A (**386**), the latter exhibiting anthelminthic activity against the nematode parasite *Nippostrongylus brasiliensis* at 50 μg/mL [424,426]. Moreover, *Chromodoris lochi* from Vanuatu contains the PKS-NRPS-derived mycothiazole (**129**) which is described to possess anthelminthic activity against the nematode parasite *N. brasiliensis* at 50 μg/mL [378]. Mycothiazole (**129**) has also been found in the prey sponge of the slug, the sponge *Spongia (Cacospongia) mycofijiensis* [379].

Among the numerous nitrogenated compounds reported from phyllidid nudibranchs, mostly obtained from their sponge prey, several have been found to have antiplasmodial activity [44,847]. Axisonitrile-3 (**25**) shows an IC_50_ towards *Plasmodium falciparum* of 16.5 ng/mL for chloroquine-resistant strain W2 and no associated cytotoxicity [848]. Axisonitrile-3 (**25**) has been reported to interfere with the detoxification of heme, a degradation product of hemoglobin digestion within infected erythrocytes, and to form a binary complex with iron in protoporphyrin IX, producing heme accumulation, which results in toxicity to the malaria parasite [849].

Pustulosaisonitrile-1 (**378**) from *Phyllidiella pustulosa* from Australia presents moderate levels of in vitro antimalarial activity [850]. Among the diverse nitrogenous mono-, bi- and tri-cyclic sesquiterpenes found in *Phyllidia ocelata* and *Phyllidiella pustulosa* from different geographical locations, several are reported to possess antimalarial activity against *Plasmodium falciparum* [102,118,119,120,122,375,376,377]. In particular, in *P. ocellata* from Australia, 2-*iso*cyanoclovene (**379**), its dihydro analogue 2-*iso*cyanoclovane (**380**) and 4,5-*epi*-10-isocyanoisodauc-6-ene (**381**) present IC_50_ values of 0.26–0.30 μM, while 1-*iso*thiocyanatoepicaryolane (**382**) has an IC_50_ > 10 μM [376]. In *P. pustulosa* from Fiji, 10-thiocyano-4-cadinene (**383**) shows moderate antiplasmodial activity [118,123]. 

#### 3.3.2. Sacoglossa 

The depsipeptides kahalalides (**194,354–356,377**) found in different *Elysia* species, such as *E. rufescens*, *E. ornata*, *E. grandifolia*, and their algal food *Bryopsis pennata*, have been reported to possess antileishmanial properties [794,795,796,797]. Kahalalides (**194,354–356,377**) are active against *Leishmania* spp. At micromolar ranges, and their lethality is linked to the alteration of the plasmatic membrane of the protozoan [794,795,796,797].

### 3.4. Antiviral Activity

Marine organisms are considered an underexplored source of antiviral compounds [851,852,853]. Many of the drugs currently employed produce strong side effects and develop resistances [853]. Viral diseases cause a huge number of deaths annually; for example, human immunodeficiency virus (HIV) is one of the top five most deadly diseases worldwide [853]. Furthermore, new viruses are appearing with extreme virulence, such as COVID-19, with no known treatment to date [854]. Therefore, the need for new antiviral drugs is clear, and heterobranchs, with their amazing biodiversity and chemodiversity, may perhaps contribute to this. To date, only doridacean nudibranchs, sea hares, and sacoglossans have been reported to possess antiviral compounds (Figure 24, Table 13).

#### 3.4.1. Nudibranchia

##### Doridacea

The chromodoridid *Cadlina luteomarginata* presents compounds with the tricyclic ansellane carbon skeleton, among other compounds, obtained from its sponge prey *Phorbas* sp. [513]. Among them, ansellone A (**216**) from the sponge was tested for the “shock and kill” approach to a sterilizing HIV-1 cure [855]. Ansellone A (**216**), together with other sponge compounds, was found to activate the latent proviral HIV-1 gene expression, as well as to possess LRA profiles comparable to prostratin, which is in phase I as a potential HIV treatment [855].

Several chromodorid species contain spongiadiol (**35**) from the sponges they feed on, among other spongian diterpenes [22,166,380,381,535,536]. This is the case of *Chromodoris mandapamensis* and *Glossodoris cincta* (*G. atromarginata*) specimens from different localities, which present these compounds in their mantle and digestive gland [22,166,380,381,535,536]. Spongiadiol (**35**) is active against the herpes simplex virus, showing an IC_50_ of 0.25 μg/mL against herpes simplex virus type I [535,537]. *Chromodoris hamiltoni* presents a wide arsenal of chemicals, among which the 2-thiazolidinone macrolides latrunculins A (**38**) and B (**37**) from its diet of sponges are found at different localities [154,156]. Latrunculin B (**37**) is also present in *C. africana* and *C. quadricolor* [170,171]. Latrunculin B (**37**), a very active compound, as previously mentioned, was reported in the sponge *Latrunculia magnifica* [168,169,170]. The EC_50_ of latrunculin B (**37**) against HIV-1 is 16.4 μM, thus showing a moderate activity while being non-cytotoxic [853]. *Doriprismatica (Glossodoris) atromarginata* also presents furanoditerpenoids and scalarane sesterterpenes originating from its dietary sponges *Spongia* (*Hyatella*) sp. and *Hyrtios* spp., depending on the geographical location (Australia, Sri Lanka, and India) [92,175,180,381,382]. Some of these compounds are reported as antivirals, particularly spongiadiol (**35**) and *epi*-spongiadiol (**232**) [180,383,384,385,386,519,535,539]. Puupehenone (**218**) from *Chromodoris petechialis* is also active against HIV-1 [797].

#### 3.4.2. Anaspidea

*Dolabella auricularia* presents some diet-derived cyclic depsipeptides reported to exhibit a wide range of activity against different stages of the HIV life cycle [718]. Dolastatin 3 (**192**) from *D. auricularia* was further isolated from the circumtropical cyanobacterium *Lyngbya majuscula* from Palau [718,728]. Dolastatin-3 (**192**) inhibits HIV-1 integrase at relatively high concentrations, with EC_50_ of 5 mM for the terminal cleavage and 4.1 mM for the strand-transfer reactions [728]. The activity of dolastatin-3 (**192**) was lost after some time in the laboratory, since it was a difficult-to-handle molecule and also presented some cytotoxicity; therefore, it was not taken forward for further investigation [728]. 

#### 3.4.3. Sacoglossa 

In this group, the previously mentioned depsipeptides kahalalides (**194,354–356,377**) are found in *Elysia rufescens*, *E. grandifolia*, and *E. ornata*, as well as in the green algae *Bryopsis pennata in* their diet [47,49]. Among them, kahalalide F (**194**) is the most bioactive compound, although all kahalalides possess many activities as reported above. Kahalalide F (**194**) presents antiviral properties against herpes simplex virus II, while kahalalides A (**377**) and G (**384**) are inactive [331,794,795,796,797]. Kahalalide F (**194**) is found along with its isomer, *iso*-kahalalide F (**354**), both displaying a wide array of bioactivities and suggested to be of bacterial origin [331,806,808]. Kahalalide F (**194**) also exhibits moderate activity against HIV-1, with an EC_50_ of 14.2 μM, while it is not cytotoxic against human peripheral blood mononuclear (PBM) cells [853]. Kahalalides A (**377**) and G (**384**), contrastingly, are not active against HIV-1 [853].

### 3.5. Anti-Inflammatory Activity

Only a few nudibranchs and some sea hares are known to possess anti-inflammatory compounds, while there have been no studies to date regarding the remaining groups (Figure 24, Table 14).

#### 3.5.1. Nudibranchia

##### Doridacea

As reported above, several species of *Glossodoris* present scalaradial (**44**) and other scalarane compounds derived from the sponges they feed on [22,166,175,180,381,383,856]. These include *Glossodoris rufomarginata*, *G. pallida*, *G. vespa*, *G. averni*, *G. hikuerensis*, *G. atromarginata*, and *G. cincta* from different geographical locations [22,166,175,178,380,381,383,536,856]. Scalaradial (**44**) is a potent anti-inflammatory compound [856], but it is also toxic to slugs, and, thus, after feeding, they quickly transform scalaradial (**44**) into its 12-deacetyl derivative or other related scalaranes in a detoxification process, locating them in MDFs in their mantle rims [2,4,22,166,176,177,383]. Scalaradial (**44**) was first found in the Mediterranean sponge *Cacospongia mollior* [857] and has been reported to display a potent inhibition of PLA_2_ [858]. Similarly, *Goniobranchus* species usually present spongian cyclic diterpenes, which are often cytotoxic, as reported above, and obtained from their diet of *Spongionella* sponges [154,190,533]. Among these compounds, *G. splendidus* contains gracilins (**224–228**), some of which have been tested from the sponge and possess a high anti-inflammatory potential, such as cyclosporine A mimics and as BACE1 and ERK inhibitors [534].

##### Dendronotida

The invasive species *Melibe viridis* presents a prostaglandin lactone in its mucus and cerata which had been previously reported in *Tethys fimbria* [77,240]. In fact, *T. fimbria*, presents a wide array of de novo biosynthesized prostaglandins (**80,81**) with different roles, which may include reducing inflammation in their tissues after autotomy and tissue regeneration [240]. 

Moreover, punaglandins (**250**) from *Tritonia* sp. show anti-inflammatory activity, and a synthetic 10-thiomethyl derivative enhances in vivo mineralization in human osteoblasts [587].

#### 3.5.2. Anaspidea

Several species of sea hares have been studied to date for anti-inflammatory activity. *Aplysia depilans* presents 8 carotenoids and 22 polyunsaturated fatty acids obtained from their algal diet and found in the digestive gland which possess anti-inflammatory activity [859]. *Aplysia dactylomela* possesses dactyloditerpenol acetate (**385**); this is probably derived from laurenditerpenol from *Laurencia intricata*, which is reported to have a significant in vitro anti-neuroinflammatory activity [860,861].

*Bursatella leachii* feeds on cyanobacteria and accumulates its natural products usually in its digestive gland, using them for its own defense as previously discussed [311,312,783,784]. Among these compounds, the alkaloid malyngamide S (**350**) from New Zealand specimens presents anti-inflammatory properties [786].

### 3.6. Against Neurodegenerative Diseases

Activity against neurodegenerative diseases has been described for several marine natural compounds [862]. In heterobranch molluscs, compounds from several species have been tested, providing some interesting results (Figure 24, Table 15). The doridacean nudibranch *Polycera atra* feeds on the bryozoan *Bugula neritina,* accumulating the polyketide macrolides bryostatins (**203**) and transferring them to its spawn, as mentioned above [492,493,494]. Bryostatins (**203**) have been further traced to the symbiont Candidatus *Endobugula sertula*, where the biosynthetic genes have been described [495]. Among them, bryostatin 1 (**203**) is the most studied molecule as a potential treatment for many diseases, including cancer and Alzheimer disease (AD), and it is in phase I trials for AD [495,497].

The doridacean nudibranchs *Goniobranchus obsoletus* and *G. splendidus* from Australia possess many cyclic diterpenes of the spongian type, including gracilins (**224–228**) which are accumulated from feeding on *Spongiella* sponges [154,190,533]. Gracilins (**224–228**), as previously mentioned, possess several interesting properties as drug candidates and also show a potential role against neurodegenerative diseases, such as AD, which is also being tested [534,863].

The chromodoridid *Cadlina luteomarginata* obtains ansellone A (**216**) from its diet of the sponge *Phorbas* sp. [513]. Ansellone A (**216**) shows cAMP activation (EC_50_ = 14 mM) comparable to that of forskolin in the HEK293 cell-based assay [513]. This activity is very useful in stem cell techniques, because modulating the cAMP signaling pathway is crucial for treating many diseases, such as cancer and heart failure, as well as neurodegenerative diseases [513].

In cephalaspideans, the cylichnidae *Scaphander lignarius* lives in soft bottom, muddy areas, usually feeding on foraminiferans [229,231]. As previously mentioned, *S. lignarius* specimens from the Mediterranean and East Atlantic present the so-called lignarenones (**171**), which are de novo biosynthesized and secreted in the Blochmann’s gland [245]. These compounds are suggested to be used as alarm pheromones, similarly to other cephalaspidean species mentioned above. Interestingly, recent studies suggest that lignarenone B (**171**) could also be used as a possible therapeutic candidate for the treatment of GSK3β-involved pathologies, such as AD [864]. In silico binding studies revealed that lignarenone B (**171**) can act over the ATP and/or substrate binding regions of GSK3β [864]. The predicted inhibitory potential of lignarenone B (**171**) was experimentally validated by an in vitro assay showing a ~50% increase in Ser9 phosphorylation levels of GSK3β, while it also potentiates structural neuronal plasticity in vitro using neuronal primary cultures [865]. Future studies are aimed to test lignarenones in preclinical mouse models of AD.

### 3.7. Other Pharmacological Activities

Other activities that were not included in the previous sections comprise those of a couple of nudibranch species and a pleurobranchoidea (Figure 24, Table 16). No other activities have been described in the remaining groups.

Janolusimide (**138**) is a tripeptide described in the Mediterranean euarminid nudibranch *Janolus cristatus* [388]. Janolusimide (**138**) is toxic to mice (LD 5 mg/kg) and affects acetylcholine receptors, thus having a neurotoxic action at lower concentrations [388]. A N-methyl analogue, janolusimide B, was further described in the New Zealand bryozoan *Bugula flabellata* [390], suggesting a dietary origin for janolusimide (**138**), since *J. cristatus* has been reported to feed on bryozoans, including *B. flabellata* [390].

The pleurobranchoid genus *Pleurobranchaea* is often used as a model for neurobiology investigations because of its peculiar escape swimming behavior, which is achieved by alternating dorsal and ventral body flexions [868]. Furthermore, it is also interesting because *P. maculata* from New Zealand possesses tetrodotoxin (TTX) (**387**). TTX (**387**) is found in its adult tissues, gonads, and egg masses, thus suggesting a defensive role [869,870]. TTX (**387**) is a very potent neurotoxin that inhibits action potential in nerve cells, and it has been found in many poisonous animals, such as flatworms, arrow worms, ribbon worms, snails, blue-ringed octopus, xanthid crabs, sea stars, fish, and toads [871,872]. In some of these cases, it has been demonstrated that TTX (**387**) is produced by symbiotic bacteria from the *Pseudoalteromonas*, *Pseudomonas*, *Vibrio*, and other strains, and that it is bioaccumulated along the food chain. However, the bacterial origin of TTX (**387**) has not been proved in all cases [871,872].

## 4. Concluding Remarks

**Figure 25:**
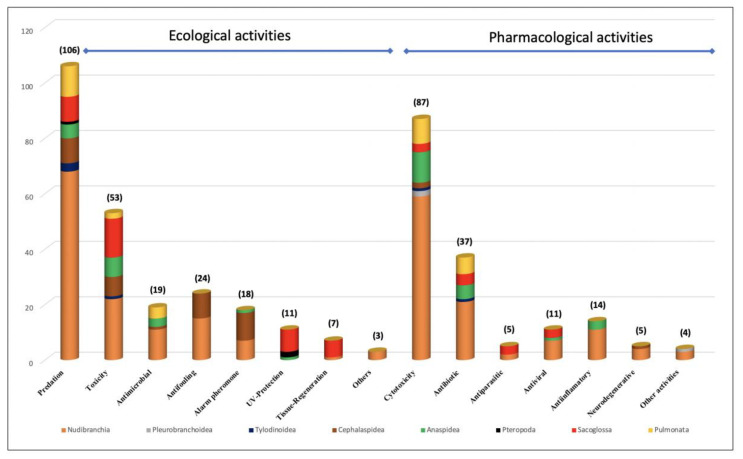
Number of species with bioactive compounds in the different heterobranch groups.

Despite the fact that only a small proportion of heterobranch molluscs has been reported to date, they represent a particularly rich group of natural products. Their NPs display an astonishing variation in bioactivity, both ecological and pharmacological, reflecting the huge chemodiversity they possess (Figure 25). Biodiversity and chemodiversity correlate here to offer a huge amount of bioactive molecules in these peculiar group of molluscs. Heterobranchs indeed comprise a very diverse group of organisms that present almost all classes of natural products described to date, but not all of these NPs have been tested for potential bioactivities [1,2,28]. Thus, many more studies are expected to find not only new NPs but also their potential bioactivities. 

Regarding ecological activity, in fact, very few compounds from the total NPs described have been tested, as described above, and this keeps the door open for many other ecological interactions to be identified in the future. NPs from heterobranchs have been shown to be ecologically relevant, as in the case of those from other marine organisms, although many experiments should still be performed to complete the available information. Some molecules are shown to display multiple roles, as is the case in other marine invertebrates, while similar structures are shown to display similar roles in geographically distant localities by phylogenetically related species [1,2]. The most studied activity is feeding deterrence, followed by toxicity (Figure 25), although the NPs are not usually tested against sympatric species, raising doubts about their real effects in the habitat where the molluscs live. Reliable field data are scarce, and, therefore, the ecological significance of many compounds remains to be demonstrated. 

The pharmacological activity of heterobranch NPs is still underexplored because we are only aware of a small part of their chemical arsenal. However, their potential is obvious from the molecules reported above, which are proven to be of interest in many fields, with some compounds being promising drugs, such as dolastatins, ulapualides, kabiramides, latrunculins, doliculides, and others. Further research is needed to develop these compounds. Overall, the most studied activities are cytotoxicity and anticancer activity, followed by antibiotic activity (Figure 25).

In this review, we discussed more than 450 metabolites isolated from ca. 400 species of heterobranch molluscs. Heterobranch molluscs are thus an important source of bioactive NPs, even if not all of them are produced by the molluscs themselves. John Faulkner once said that in order to find the most bioactive compounds in an ecosystem, heterobranchs would be the best shot to find them, because they have already selected them along evolution. This continues to appear to be true. While symbionts may be behind some of the NP syntheses, heterobranchs have evolved to use them for their own benefit [1,2]. In some cases, dietary cyanobacteria or other bacteria have been proven to be the source of compounds, but, in general, origin from symbionts remains difficult to prove [481]. In any case, if NPs can be traced to a microorganism, this may help to solve the supply problems for many of the bioactive NPs, either by culturing, by isolating the BGCs (biosynthetic gene clusters), or by using other metagenomic techniques [873,874]. Culturing the molluscs is a difficult-but-not-impossible task, which could also be useful for some heterobranch species. Moreover, possible strategies to improve MNP selection include many dereplication strategies described in the literature, in addition to the many chemical techniques used to obtain derivatives, as well as synthesis methods [44,761,875]. Furthermore, virtual screening, computational chemistry, as well as more studies on molecular targets are needed to overcome the limitations of studying MNPs. The use of nanotechnology to deliver drugs is also a promising field that requires further investigation; ADCs, for instance, show considerable potential [481]. As an example, kahalalide F (**194**) conjugated to 40 nm gold nanoparticles resulted in higher cell growth inhibition in HeLa cervical carcinoma cells than the compound alone [481,876]. Further research should also be devoted to this field. Overall, we have seen that heterobranch molluscs are extremely interesting in regard to the study of marine natural products in terms of both chemical ecology and biotechnology studies, providing many leads for further detailed research in these fields in the near future.

## Figures and Tables

**Table 1:** Species number and natural products numbers (NPs) for the different heterobranch groups [2,49,50]. * Accepted species number obtained from WoRMs (www.marinespecies.org), accessed on 11 November 2020). ** Natural products’ number, main types of molecules, and diet according to Avila et al. [2]. *** Only marine pulmonata are considered here. **#** Number.

Phylum Mollusca Class Gastropoda Subclass Heterobranchia	Species #* 80548 33193	NPs #**	Main Types of Molecules **	Main Diet **
Nudibranchia	2462	~250	Terpenoids, alkaloids, macrolides, peptides, acidic secretions, etc.	Porifera, bryozoa, tunicata, cnidaria, other heterobranchs, crustacea, turbellaria
Pleurobranchoidea	96	25	Terpenoids, alkaloids, peptides, acidic secretions, etc.	Tunicates, other animals
Tylodinoidea	12	6	Alkaloids, diacylglycerols, etc.	Porifera
Cephalaspidea	875	40	Polyketides, polypropionates, polyacetates, ethers, acidic secretions, etc.	Algae, other heterobranchs, porifera, other animals
Anaspidea	94	~200	Polyketides, terpenoids, peptides, etc.	Algae, sea grasses, cyanobacteria
Pteropoda	409	5	Polypropionates, etc.	Phytoplankton, other pteropods
Sacoglossa	362	~120	Terpenoids, polypropionates, etc.	Algae
Pulmonata	500 ***	~75	Polypropionates, terpenoids, peptides, etc.	Algae

**Table 2:** Natural products used against predation in the different heterobranch groups. In brackets: number of species with antipredatory compounds, number of the compounds in figures, and reference numbers. **#** Number.

Species (#)	Compounds (#)	Predator(s) Tested	References (#)
**Nudibranchia (68)**			
*Bathydoris hodgsoni*	Hodgsonal (**1**)	Sea star *Odontaster validus,* anemone *Epiactis* sp.	[54,55,56]
*Doris (Austrodoris) kerguelenensis*	Diterpene diacylglycerides (**2**)	Sea star *Odontaster validus,* anemone *Epiactis* sp.	[58,59,60]
*Aldisa sanguinea*	3-Oxo-chol-4-ene-24-oic acid (**3**), unsaturated analogue (**4**)	Goldfish (*Carassius auratus*)	[73]
*Aldisa andersoni*	9-Chloro-phorbazole D (**5**), N1-methyl-phorbazole A (**6**), phorbazoles A (**7**), B (**8**), and D (**9**)	Shrimp	[54,56,75,76,77]
*Sclerodoris tanya*	Tanyolides A (**10**) and B (**11**)	Fishes (*Gibbonsia elegans* and *Paraclinus integrippinis*)	[78]
*Paradoris (Discodoris) indecora*	Variabilin (**12**)	Marine and freshwater fishes	[79]
*Dendrodoris limbata*	Polygodial (**13**), olepupuane (**14**)	Marine and freshwater fishes	[80,81,83,84]
*Dendrodoris arborescens*	7-Deacetoxyolepupuane (**15**)	Feeding deterrence	[87]
*Dendrodoris carbunculosa*	Dendrocarbins A–N (**16**)	Feeding deterrence	[86]
*Dendrodoris denisoni*	Cinnamolide (**17**), olepupuane (**14**), polygodial (**13**)	Fish	[88]
*Doriopsilla pelseneeri*	Pelseneeriols 1 (**18**) and 2, polygodial (**13**)	Feeding deterrence	[95,99]
*Doriopsilla albopunctata, D. areolata, D. janaina, D. pharpa*	*ent*-pallescensin A (**19**), 15-acetoxy-*ent*-pallescensin (**20**)	Feeding deterrence	[96,97,99]
*Doriopsilla pharpa*	Polygodial (**13**)	Fishes (*Chasmodes bosquianus, Fundulus heteroclitus*), crabs (*Callinectes similus, Panopeus herbstii*)	[98]
*Phyllidia varicosa*	9-*Iso*cyanopupukeanane (**21**), 2-*iso*cyanopupukeanane (**22**)	Fish	[106,107,108]
*Phyllidia coelestis, Phyllidiella pustulosa*	Xidaoisocyanate A (**24**)	Fish	[117]
*P. pustulosa*	Axisonitrile-3 (**25**), amphilectene (**26**), kalihinol A (**27**), kalihinol E (**28**), *ent*-stylotelline (**29**)	Goldfish *(C. auratus)*	[118,120,123]
*Cadlina luteomarginata*	Isocyanides (**30**), albicanyl acetate (**31**), cadlinaldehyde (**32**), luteone (**33**), 1a,2a-diacetoxyalbicanyl acetate (**34**)	Fishes (*Carassius auratura, Clinocottus analis*)	[126,127,128,129,130]
*Chromodoris africana, C. (Glossodoris) quadricolor*	Kurospongin (**36**), latrunculin B (**37**)	Fish (*Tilapia mosambica*)	[167,168,170,171]
*Chromodoris hamiltoni*	Latrunculins A (**38**) and B (**37**), hamiltonins A–E (**41**,**42**)	Feeding deterrence	[153,155]
*Glossodoris vespa, G. averni, G. pallida*	12-Deacetoxy-12-oxoscalaradial (**43**)	Shrimp (*Palaemon serenus*)	[175,176,177]
*Glossodoris pallida*	Scalaradial (**44**), deacetylscalaradial (**45**), deoxoscalarin (**46**)	Crabs (*Leptodius* sp.), fish (*Abudefduf sexfasciatus*)	[176,177,178]
*Ceratosoma trilobatum, C. gracillimum*	Pallescensin B (**47**), (–)-furodysinin (**48**), (–)-dehydroherbadysidolide (**49**), (–)-herbadysidolide (**50**), nakafuran-9 (**51**), dendrolasin (**55**), furodysin (**58**), agassizin (**59**)	Goldfish (*C. auratus*)	[131,193,194,195,196]
*Felimare (Hypselodoris) picta webbi, F. (Hypselodoris) villafranca, F. (Hypselodoris) cantabrica, F. (Hypselodoris) tricolor, F. (Hypselodoris) fontandraui*	Longifolin (**52**)	Shrimp (*P. elegans*)	[124,165]
*Felimare (Hypselodoris) fontandraui*	Tavacpallescensin (**53**)	Shrimp (*P. elegans*)	[205,211]
*Hypselodoris capensis*	Nakafuran-8 and -9 (**54**,**51**)	Feeding deterrence	[213]
*Hypselodoris obscura*	Dendrolasin (**55**), (–)-euryfuran (**56**), (+)-pallescensin A (**57**), (–)-furodysinin (**48**), (–)-furodysin (**58**)	Feeding deterrence	[186]
*Hypselodoris whitei*	(–)-Euryfuran (**56**), (–)-furodysin (**58**), (–)-furosydinin (**48**), dendrolasin (**55**)	Feeding deterrence	[186]
*Hypselodoris infucata*	Nakafuran-8 and -9 (**54**,**51**), (–)-furodysinin (**48**)	Shrimp (*Penaeus vannamei*)	[157,165,186]
*Hypselodoris benneti*	Euryfuran (**56**), agassizin (**59**), dehydroherbadysidolide (**49**), pallescensone (**60**)	Feeding deterrence	[178]
*Hypselodoris (Risbecia) tryoni*	Dehydroherbadysidolide (**49**), furodysinin (**48**), nakafuran-9 (**51**), dendrolasin (**55**)	Feeding deterrence	[178]
*Hypselodoris kanga*	Furodysinin (**48**)	Feeding deterrence	[166]
*Hypselodoris lajensis*	Furodysinin lactone (**61**)	Feeding deterrence	[207]
*Mexichromis festiva*	Euryfuran (**56**), dendrolasin (**55**)	Feeding deterrence	[178]
*Mexichromis marieri*	Euryfuran (**56**)	Feeding deterrence	[178]
*Hexabranchus sanguineus*	Kabiramide C (**62**), dihydrohalichondramide (**63**), sanguinamides A (**64**) and B	Fish (*Thalassoma lunare),* crab (*Dardanus megistos)*	[217,218,219,220,221,222]
*Tambja abdere, T. eliora*	Tambjamines A–F (**65**–**70**), tambjamine aldehyde (**71**)	Fish	[160,224,225]
*Roboastra tigris, Nembrotha* spp.	Tambjamines A–F (**65**–**70**), tambjamine aldehyde (**71**), tetrapyrrol (**72**)	Fish	[137,157,223,226,227]
*Tritonia hamnerorum*	Julieannafuran (**73**)	Fish	[229]
*Tritoniella belli*	1-O-hexadecyl glycerol (**74**)	Seastar (*O. validus*)	[230]
*Tritoniopsis elegans*	Tritoniopsins A–D (**75**–**78**)	Feeding deterrent	[234]
*Marionia blainvillea*	Homarine (**79**)	Feeding deterrent	[235]
*Tethys fimbria, Melibe viridis*	PGE_2_-1,15-lactone (**80**), PGE_3_-1,15-lactone (**81**)	Feeding deterrent	[237,238]
*Charcotia granulosa*	Granuloside (**82**)	Seastar (*O. validus*)	[241,242]
*Cratena pilata, Cuthona gymnota, Hermissenda crassicornis, Phestilla lugubris, Cuthona coerulea, Flabellina exoptata, F. ischitana, F. pedata, F. affinis*	Homarine (**79**)	Feeding deterrent	[235]
*Phyllodesmium magnum, Phyllodesmium guamensis*	11β-Acetoxypukalide (**83**)	Pufferfish (*Canthigaster solandri*)	[236,249]
*Phyllodesmium longicirrum*	Trocheliophorol (**84**), 4-oxochatancin (**85**), (2S)-*iso*sarcophytoxide (**86**), cembranoid bisepoxide 12	Pufferfish (*Canthigaster solandri*)	[245,250,251]
**Tylodinoidea (3)**			
*Tylodina fungina, T. perversa*	3,5-Dibromotyrosine (**87**)	Feeding deterrent	[256,257]
*Tylodina corticalis*	Bromotyrosine-derived alkaloids	Feeding deterrent	[258]
**Cephalaspidea (9)**			
*Bulla striata, Philinopsis depicta*	Aglajnes 1–3 (**88**)	Fish *(C. auratus)*	[269]
*Bulla gouldiana, Navanax inermis*	Pulo’upone (**90**)	Feeding deterrent	[278]
*Aglaja tricolorata*	Homarine (**79**)	Reef fish	[235]
*Haminoea cymbalum*	Kumepaloxane (**91**), tetrahydropyran	Porcupine fish	[280]
*Haminoea cyanomarginata*	Tetrahydropyran	Shrimp *(P. elegans)*	[77]
*Sagaminopteron nigropunctatum, S. psychedelicum*	3,5 Dibromo-2-(2′,4′-dibromo-phenoxy)phenol (**92**)	Feeding deterrent	[282]
**Anaspidea (5)**			
*Aplysia californica, A. dactylomela, A. parvula*	Aplysioviolin (**93**), phycoerythrobilin	Blue crabs, lobsters	[289,290,292]
*Aplysia parvula*	Apakaochtodene A (**94**) and B, costatone (**95**)	Fish	[88,299,300]
*Stylocheilus longicauda*	Aplysiatoxin (**96**), debromoaplysiatoxin (**97**), stylocheilamide (**98**), makalika ester (**99**), makalikone ester (**100**), lyngbyatoxin A acetate (**101**), malyngamide A (**104**), malyngamide B, malyngamide O (**102**), and malyngamide P (**103**)	Fish, amphipods, crabs, cephalaspidean	[302,303]
*Bursatella leachii*	Bursatellin (**105**)	Fish (*Oreochromis mossambicus* and *Caffragobius gilchristi*)	[311,312]
**Pteropoda (1)**			
*Clione limacina*	Pteroenone (**106**)	Fish	[314]
**Sacoglossa (9)**			
*Ascobulla ulla*	Ascobullin A (**107**) and B	Feeding deterrent	[316]
*Elysia crispata*	Crispatenine, onchidal (**108**)	Feeding deterrent	[131]
*Elysia translucens*	Udoteal	Fish (*Pomacentrus coeruleus*)	[320]
*Thuridilla hopei*	Thuridillins (**110**), *nor*-thuridillonal (**111**), epoxylactone	Shrimp (*P. elegans*)	[123,321,322]
*Costasiella ocellifera*	Avrainvilleol (**112**)	Fish	[316,325]
*Cyerce cristallina, C. nigricans*	Cyercenes (**113**), chlorodesmin (**114**)	Mosquito fish (*Gambusia affinis*)	[326,327,328]
*Mourgona germaineae*	Cyclocymopol (**115**)	Fish	[330]
*Placida dendritica*	Polypropionate γ-pyrones (**116**)	Feeding deterrent	[332]
**Pulmonata (11)**			
*Onchidella binneyi*	Onchidal (**108**), ancistrodial (**109**)	Fish and crabs	[319]
*Peronia peronii, Onchidium ssp.*	Onchidin (**121**), onchidione (**122**), onchidiol, 4-*epi*-onchidiol	Sea stars	[343,358,359,361,362]
*Trimusculus costatus*	Labdanes 6β,7a-diacetoxylab-8,13-dien-15-ol (**117**), 2α,6β,7a-triacetoxylabda-8,13-dien-15-ol (**118**)	Fish (*Pomadasys commersonnii)*	[333]
*Trimusculus reticulatus, T. costatus, T. peruvianus*	6β-*iso*valeroxylabda-8,13-dien-7α, 15-diol, 2α,7α-diacetoxy-6/3-*iso*valeroxylabda-8,13-dien- 15-ol	Sea stars	[334,335,336,337]
*Siphonaria capensis, S. concinna, S. cristatus, S. serrata*	Siphonarienolone (**119**), siphonarin A (**120**), diemenensins A and B	Fish	[341,342,344,347,350]

**Table 3:** Number of toxic compounds in the different heterobranch groups. In brackets: number of species with toxic compounds, number of the compounds in figures, and reference numbers. # NumberSpecies (**#**)**.**

	Compounds (#)	Activity	References (#)
**Nudibranchia (22)**			
*Archidoris pseudoargus*	Diterpenoic acid glycerides (**123**)	Ichthyotoxicity	[363,366]
*Doris verrucosa*	Verrucosins A (**124**) and B	Ichthyotoxicity, potent activators of PKC, and promotion of tentacle regeneration in the freshwater hydrozoan *Hydra vulgaris*	[371,420]
*Phyllidia varicosa*	2-*Iso*cyanopupukeanane (**22**), 9-*iso*cyanopupukeanane (**21**), 2-*iso*cyanoallopupukeanane (**125**), 9-Thiocyanatopupekeanane (**126**)	Toxic to brine shrimp, killifish (*Oryzias latipes*), and crustaceans	[106,110,123]
*Phyllidia pulitzeri*	Axisonitrile-1 (**127**)	Toxic to fish (*Chromis chromis* and *Carassius carassius*)	[184]
*Phyllidiella rosans (P. bourguini)*	9-*Iso*cyanopupukeanane (**21**), *epi*-9-*iso*cyanopupukeanane	Ichthyotoxic to killifish *Oryzias latipes*	[373]
*Cadlina luteomarginata*	Isocyanides (**30**), isothiocyanates (**128**)	Toxic to goldfish (*Carassius auratus*)	[126,127]
*Chromodoris africana*	Kurospongin (**36**)	Ichthyotoxicity	[167,168,169]
*Chromodoris africana, C. quadricolor*	Latrunculins A (**38**) and B (**37**), kurospongin (**36**), 2-thiazolidinone	Ichthyotoxicity	[167,170,171]
*Felimida (Chromodoris) luteorosea*	Norrisolide (**130**), polyrhaphin C (**131**), chelonaplysin C (**132**), luteorosin (**133**), macfarlandin A (**134**)	Ichthyotoxicity	[149]
*Doriprismatica (Glossodoris) sedna*	12-Deacetyl-23-acetoxy-20-methyl-12-*epi*-scalaradial (**135**), 12-deacetyl-23-acetoxy-20-methyl-12-*epi*-deoxoscalarin, 12-deacetyl-20-methyl-12-*epi*-deoxoscalarin	Ichthyotoxic to *Gambusia affinis*	[183,421]
*Doriprismatica (Glossodoris) atromarginata*	12-Deacetoxy-12-oxodeoxoscalarin (**136**)	Ichthyotoxic to mosquito fish (*G. affinis*)	[380,381]
*Felimida (Glossodoris) dalli, Glossodoris rufomarginata, Glossodoris pallida, Glossodoris vespa, Ardeadoris (Glossodoris) averni*	Homoscalarane, scalarane, 12-deacetyl-23-acetoxy-20-methyl-12-*epi*scalaradial (**135**)	Ichthyotoxic to mosquito fish (*G. affinis*)	[175,183,383]
*Ceratosoma trilobatum, C. gracillimum*	Pallescensin B (**47**), (-)-furodysinin (**48**), (-)-dehydroherbadysidolide (**49**), (-)-herbadysidolide (**50**), nakafuran-9 (**51**)	Ichthyotoxicity	[22,131,193]
*Tethys fimbria, Melibe viridis*	Prostaglandin-1,15-lactones (**80**)	Ichthyotoxic to mosquito fish (*G. affinis*)	[77,240]
*Dermatobranchus ornatus*	Ophirin (**137**), calicophirin B, 13-deacetoxyl calicophirin B, 13-deacetoxyl-3-deacetyl calicophirin B	Inhibitory activity against the growth of silkworm *Bombyx mori*, and inhibition of cell division in fertilized starfish eggs	[387,422,423]
*Janolus cristatus*	Janolusimide (**138**)	Toxic to mice	[388,390]
**Tylodinoidea (1)**			
*Umbraculum mediterraneum*	Umbraculumins A–C (**139**)	Ichthyotoxic to mosquito fish (*G. affinis*)	[263,391,392,393]
**Cephalaspidea (7)**			
*Bulla gouldiana*	Niuhinone B, *iso*pulo’upone (**140**)	Ichthyotoxicity and shrimp toxicity	[278]
*Bulla occidentalis*	Niuhinone B	Ichthyotoxicity and shrimp toxicity	[274]
*Navanax inermis*	Niuhinone-B, *iso*pulo’upone (**140**), 5,6-dehydroagajne-3 (**141**)	Ichthyotoxicity and shrimp toxicity	[278]
*Philinopsis depicta*	Niuhinone B, aglajne 3 (**88**)	Toxic to *G. affinis* and *Artemia salina*	[270]
*Philinopsis speciosa*	Niuhinone A, B, pulo’upone (**90**), kulolide-1 (**271**), pupukeamide, tolytoxin-23-acetate	Ichthyotoxicity and shrimp toxicity	[272,273,275,276,277]
*Haminoea cyanomarginata*	Brominated tetrahydropyran (**142**)	Ichthyotoxic to mosquito fish (*G. affinis*)	[77]
*Haminoea cymbalum*	Brominated tetrahydropyran (**142**), kumepaloxane (**91**)	Ichthyotoxic to mosquito fish (*G. affinis*)	[280]
**Anaspidea (7)**			
*Aplysia fasciata*	4-Acetylaplykurodin-B (**143**), aplykurodinone B (**144**), 3-*epi*-aplykurodinone B (**145**)	Ichthyotoxicity	[396]
*Aplysia juliana*	Pyropheophorbides a and b, halogenated diterpenoid lactone, julianin-S	Lethal to crabs	[288]
*Aplysia kurodai*	Aplykurodin B (**146**)	Ichthyotoxicity	[398]
*Aplysia parvula*	Aplyparvunin (**147**), (3*Z*)-bromofucin (**148**)	Ichthyotoxicity	[399,400]
*Aplysia vaccaria*	Crenulides (**149**)	Ichthyotoxicity	[401,402]
*Aplysia depilans*	Aplyolides A−E (**150**–**151**)	Ichthyotoxicity	[403]
*Stylocheilus longicauda*	Makalika ester (**99**), makalikone ester (**100**), malyngamide I (**152**), malyngamide O (**102**), malyngamide P (**103**), lyngbyatoxin A acetate (**101**)	Ichthyotoxicity	[302,303,304]
**Sacoglossa (15)**			
*Oxynoe panamensis*	Caulerpicin (**153**), caulerpin (**154**)	Toxic to rats and mice	[406]
*Oxynoe olivacea, Ascobulla fragilis*	Caulerpenyne (**155**), oxytoxin 1 (**156**) and 2	Ichthyotoxicity	[316,407]
*Lobiger serradifalci*	Oxytoxin 1 (**156**)	Ichthyotoxicity	[316,407]
*Ascobulla ulla*	Ascobullin A (**107**) and B		[316]
*Ascobulla ulla, Oxynoe antillarum, Lobiger souberveii, Volvatella* sp., *Elysia subornata, E. patina, E. nisbeti*	Caulerpenyne (**155**), oxytoxin-1 (**156**)	Ichthyotoxicity	[316,409]
*Elysia expansa*	Caulerpenyne (**155**), dihydrocaulerpenyne, expansinol	Ichthyotoxicity	[411]
*Costasiella ocellifera (C. lilianae)*	Avrainvilleol (**112**)	Toxic to sympatric reef fishes	[325]
*Placida dendritica*	*Iso*-placidene-A (**116**)	Strong ichthyotoxicity against *Gambusia affinis*	[332]
*Cyerce cristallina*	Cyercene A (**157**) and B, cyercenes 1–5 (**158**,**159**)	Strong ichthyotoxicity against *G. affinis*	[326,327]
**Pulmonata (2)**			
*Siphonaria maura*	Vallartanones B	In laboratory assays against krill and fish (*Thallasoma lunare*)	[350]
*Trimusculus costatus*	6β,7a-Diacetoxylab-8,13-dien-15-ol (**117**), 2α,6β,7a-triacetoxylabda-8,13-dien-15-ol (**118**)	Toxic to brine shrimp (*Artemia salina)*	[333]

**Table 4:** Antimicrobial compounds in the different heterobranch groups. In brackets: number of species with antimicrobial compounds, number of the compounds in figures, and reference numbers. # Numbers.

Species (#)	Compounds (#)	Activity	References (#)
**Nudibranchia (11)**			
*Notodoris citrina, Notodoris gardineri*	*Iso*-naamidine-A (**160**)	Inhibits the AI-2 channel of the marine pathogen *Vibrio harveyi*	[123,426]
*Phyllidiella pustulosa, Phyllidia coelestis, Phyllidia varicosa, Phyllidia* sp.	Xidaoisocyanate A (**24**), axisonitrile-3 (**25**), 4-*iso*cyano-9-amorphene, 9-thiocyanato-pupukeanane (**126**), 3-*iso*cyanotheonellin (**161**)	Antimicrobial	[103,110,114,115,117,118,427]
*Marionia blainvillea, Phestilla lugubris, Cuthona caerulea*	Homarine (**79**)	Antimicrobial	[235]
**Cephalaspidea (1)**			
*Aglaja tricolorata*	Homarine (**79**)	Antibacterial	[235]
**Anaspidea (3)**			
*Aplysia punctata*	Glandulaurencianols A–C (**162**,**163**), punctatol (**164**)	Antibacterial	[429,430]
*Aplysia juliana*	Pyropheophorbides a and b, halogenated diterpenoid lactone, julianin-S	Antibacterial	[288,432,433]
*Dolabella auricularia*	Dolabellanin A	Antibacterial	[435]
**Pulmonata (4)**			
*Siphonaria* spp.	Siphonarienolone (**119**), diemenensins A (**165**) and B, siphonarin A (**120**), Vallartanones A and B	Antimicrobial	[27,340,344,348]
*Siphonaria diemenensis*	Diemenensin A (**165**)	Antibacterial	[341]
*Siphonaria pectinata*	Pectinatone (**166**)	Antimicrobial	[341,343]

**Table 5:** Antifouling compounds in the different heterobranch groups. In brackets: number of species with antifouling compounds, number of the compounds in figures, and reference numbers. # Numbers.

Species (#)	Compounds (#)	Activity	References (#)
**Nudibranchia (15)**			
*Phyllidia varicosa, Phyllidia rosans (P. bourguini)*	9-Isocyanopupukeanane (**21**), 3-isocyanotheonellin (**161**)	Antifouling against barnacle larvae	[93,106]
*Phyllidia sp.*	3-Isocyanotheonellin (**161**)	Antifouling activity against barnacle larvae	[114,115]
*Phyllidiella pustulosa*	Sesquiterpene isonitrile	Antifouling against barnacle larvae	[101]
*Phyllidia ocelata, P. varicosa, P. coelestis, P. picta, Phyllidiella pustulosa, Phillidiopsis krempfi*	10-epi-Axisonitrile-3, 10-isocyano-4-cadinene, 2-isocyanotrachyopsane, 1,7-epidioxy-5-cadinene, 4-isocyano-9-amorphene and 10α-isocyano-4-amorphene, 9-thiocyanatopupukeanane sesquiterpenes	Antifouling activity against barnacle larvae	[110,112]
*Reticulidia fungía*	Reticulidin A (**215**)	Antifouling activity	[438]
*Marionia blainvillea, Phestilla lugubris, Cratena pilata, Cuthona caerulea, Cuthona gymnota, Hermissenda crassicornis*	Homarine (**79**)	Antifouling activity, prevents microbial colonization of the slug mucus	[235,428,439,441,442]
**Cephalaspidea (9)**			
*Aglaja tricolorata*	Homarine (**79**)	Antifouling activity	[235,441]
*Sagaminopteron nigropunctatum, S. psychedelicum*	3,5 Dibromo-2-(2′,4′-dibromo-phenoxy)phenol (**92**)	Antifouling activity against marine bacteria, diatoms, barnacle larvae, and mussel juveniles	[282,443]
*Haminoea cyanomarginata, H. cymbalum *	Brominated tetrahydropyran (**142**)	Antifouling activity	[77]
*Haminoea orteai*	Haminol A,B,C (**167**–**168**)	Antifouling activity	[444]
*Haminoea orbignyana*	Haminol 1-2 (**169**), haminol A and B (**167**–**168**)	Antifouling activity against larvae of the barnacle Amphibalanus amphitrite	[444,445,446]
*Haminoea fusari*	Haminol 1–6 (**169**)	Antifouling activity	[445]

**Table 6:** Alarm signal compounds in the different heterobranch groups. In brackets: number of species with alarm signal compounds, number of the compounds in figures, and reference numbers. # Numbers.

Species (#)	Compounds (#)	Activity	References (#)
**Nudibranchia (7)**			
*Tambja abdere, T. eliora, Roboastra tigris*	Tambjamines (**65**–**70**)	Alarm pheromones and cues	[223]
*Tambja ceutae, Tambja stegosauriformis, Nembrotha spp.*	Tambjamines (**65**–**70**)	Alarm pheromones and cues	[226,227,228]
**Cephalaspidea (10)**			
*Navanax inermis*	Navenones A–C (**170**)	Alarm pheromones	[394,448]
*Haminoea exigua, H. fusari, H. orbignyana, H. orteai, H. navicula*	Haminols (**167**–**169**)	Alarm pheromones employed during cross copulation, escape reaction in conspecifics	[19,449]
*Haminoea navicula*	Haminols A and B (**167**,**168**)	Alarm pheromones	[449]
*Haminoea orteai*	Haminols A–C (**167**,**168**)	Alarm pheromones	[444]
*Haminoea fusari, H. hydatis*	Haminols 1-6 (**169**)	Alarm pheromones	[271,445]
*Haminoea japonica *	Navenone-C (**170**)	Alarm pheromones	[271,445]
*Haminoea callidegenita*	Haminols 7-11	Alarm pheromones	[271,451]
*Scaphander lignarius*	Lignarenones (**171**)	Alarm pheromones	[453,454,455]
**Anaspidea (1)**			
*Aplysia californica*	Aplysiapalythines A–C (mycosporine-like amino acids, MAAs), asterina, palythine	Alarm cues, causing avoidance behaviors in neighboring conspecifics	[457,458]

**Table 7:** Photoprotective compounds in the different heterobranch groups. In brackets: number of species with photoprotective compounds, number of the compounds in figures, and reference numbers.

Species (#)	Compounds (#)	Activity	References (#)
**Anaspidea (1)**			
*Aplysia californica*	Aplysiapalythines A, B, C (mycosporine-like amino acids, MAAs), asterine, palythine	Sunscreens	[457]
**Pteropoda (2)**			
*Limacina helicina, Clione limacina*	Mycosporine-like amino acids (MAAs)	UV photoprotection	[461]
**Sacoglossa (8)**			
*Cyerce cristallina*	Cyercene A (**157**) and B, cyercenes 1–5 (**158**,**159**)	Protection against sunlight-induced damage	[326,327]
*Elysia patagonica*	Phototridachiapyrone J (**172**)	Sunscreens	[412]
*Elysia crispata*	Tridachiahydropyrone (**173**), phototridachiahydropyrone (**174**)	Sunscreens	[317,464]
*Elysia (Tridachiella) diomedea*	Tridachiapyrones A–F (**175**,**176**), elysiapyrones (**177**)	Sunscreens, photoprotection	[417,418,419]
*Elysia viridis, E. chlorotica*	Elysione (**178**)	Sunscreens	[463,465]
*Placobranchus ocellatus, Placobranchus* sp.	9,10-Deoxy-tridachione (**179**), photodeoxytridachione (**180**), tridachiahydropyrone B and C (**181**), *iso*-9,10-deoxy-tridachione, ocellapyrones (**182**)	Sunscreens	[466,468,469]

**Table 8:** Tissue-regenerator compounds in the different heterobranch groups. In brackets: number of species with tissue-regenerator compounds, number of the compounds in figures, and reference numbers. # Numbers.

Species (#)	Compounds (#)	Activity	References (#)
**Nudibranchia (1)**			
*Tethys fimbria*	PGE2-1,15-lactone (**80**), PGE3-1,15-lactone (**81**)	Predator distraction by the release of their cerata, cerata regeneration	[237,238]
**Sacoglossa (6)**			
*Ercolania viridis*	Cyercenes (**113**, **157**–**159**)	Cerata autotomy, cerata regeneration	[332]
*Cyerce cristallina, C. nigricans *	Cyercenes (**113**, **157**–**159**)	Cerata autotomy, cerata regeneration	[326,327,328]
*Aplysiopsis formosa*	Aplysiopsenes A–D (**183**,**184**)	Cerata autotomy and cerata regeneration	[475]
*Mourgona germaineae, Costasiella ocellifera*	Prenylated bromohydroquino-nes	Cerata autotomy	[325,330]

**Table 9:** Other ecological activities in the different heterobranch groups. In brackets: number of species with active compounds, number of the compounds in figures, and reference numbers. **#** Number.

Species (#)	Compounds (#)	Activity	References (#)
**Nudibranchia (3)**			
*Doris kerguelenensis*	Austrodoral (**185**), austrodoric acid (**186**)	Stress metabolites	[64,65]
*Tethys fimbria*	PGs–lactones (**80**,**81**)	Role in reproduction	[237,238]
*Dermatobranchus ornatus*	Eunicellin, ophirin (**187**), calicophirin B (**188**), 13-deacetoxycalicophirin B, 13-deacetoxy-3-deacetylcalicophirin	Inhibition of cell division in fertilized starfish eggs	[22,243,387,477]

**Table 10:** Cytotoxic and antitumoral compounds in the different heterobranch groups. In brackets: number of species with these compounds, number of the compounds in figures, and reference numbers. # Numbers.

Species (#)	Compounds (#)	Activity	References (#)
**Nudibranchia (59)**			
*Doris kerguelenensis*	Palmadorins A (**195**), B (**196**), D (**197**), M (**198**), N (**199**), and O (**200**)	Inhibition of human erythroleukemia cells (HEL), inhibition of Jak2, STAT5, and Erk1/2 activation in HEL cells	[66,67,824]
*Doris verrucosa*	Verrucosins A (**124**) and B	Activation of protein kinase C	[371,420]
*Notodoris citrina, N. gardineri*	Naamidine A (**201**), iso-naamidine-A (**160**)	Inhibition of the epidermal growth factor (EGF), inhibition of human tumor xenografts in mice, and promotion of caspase-dependent apoptosis in tumor cells	[424,489,490]
*Adalaria loveni*	Lovenone (**202**)	Cytotoxic to two HTCLs	[491]
*Polycera atra*	Bryostatins (**203**)	Cytotoxic to P388 lymphocytic leukemia and Alzheimer’s disease cells	[424,486,487]
*Actinocyclus papillatus*	(–)-Actisonitrile (**204**), actinofide (**205**)	Cytotoxic to tumor and non-tumor cells	[361,479,504,503]
*Aldisa andersoni*	9-Chloro-phorbazole D (**5**), N1-methyl-phorbazole A (**6**), phorbazoles A (**7**), B (**8**), and C	Cytostatic effects in vitro against several HTCLs (human SKMEL-28 melanoma and U373 glioblastoma cells)	[55,75,76]
*Dendrodoris carbunculosa*	Dendocarbins A–N (16, 206), isodrimeninol (**207**), 11-epivaldiviolide (**208**)	Cytotoxic to murine leukemia P388 cell lines	[86,509]
*Phyllidiella coelestis*	1-Formamido-10(1→2)-abeopupukeanane (**209**), 2-formamidopupukeanane (**210**)	Cytotoxic to HeLa, MCF-7, KB, HT-29 cancer cell lines	[111]
*Phyllidiella pustulosa*	Axinisothiocyanate K (**211**), isothiocyanate axisonitrile-3 (**25**)	Cytotoxic to NBT-T2 cells	[123,377]
*Phyllidiella coelestis, P. pustulosa*	Bisabolane-type sesquiterpenoid (**212**), theonellin isothiocyanate (**213**), 7-isocyano-7,8-dihydro-α-bisabolene (**214**)	Cytotoxic to several HCCLs	[117]
*Reticulidia fungia*	Reticulidins A (**215**) and B	Cytotoxic in vitro to KB cells and mouse L1210 leukemia cells	[438,511,512]
*Cadlina luteomarginata*	Ansellone A (**216**)	Activation of the cyclic adenosine monophosphate (cAMP) signaling pathway	[513]
*Chromodoris elisabetina, C. hamiltoni, C. lochi, C. africana, C. annae, C. kuiteri, C. magnifica, C. quadricolor*	Latrunculins A (**38**) and B (**37**)	Disruption of normal cell organization and function	[153,155,168,169,170,171]
*Chromodoris lochi*	Mycothiazole (**129**)	Inhibition of the hypoxia-inducible factor-1 (HIF-1), and suppression of the mitochondrial respiration at complex I	[378,379,825,826]
*Chromodoris lochi*	Laulimalide (**39**), isolaulimalide (**40**)	Cytotoxic to the KB cell line	[142,523,524]
*Chromodoris inornata *	Inorolides A–C (**217**)	Cytotoxic to murine L1210 leukemia and human epidermoid carcinoma KB cell lines	[156,531]
*Chromodoris petechialis*	Puupehenone (**218**)	Human peripheral blood mononuclear (PBM) cells	[827]
*Goniobranchus splendidus*	Epoxygoniolide-1 (**219**)	Cytotoxic	[532]
*Goniobranchus (Chromodoris) sinensis*	Aplyroseol-2 (**220**)	Cytotoxic to HeLa S3 cells	[131]
*Goniobranchus reticulatus*	Spongian-16-one (**221**), aplytandiene-3 (**222**), aplysulfurin (**223**), aplyroseol-2 (**220**), gracilins A (**224**), B (**225**), C (**226**), G (**227**), and M (**228**)	Cytotoxic to P388 mouse leukemia, HTCLs cell lines, and BACE1 and ERK inhibition	[161,190]
*Goniobranchus (Chromodoris) obsoleta*	Dorisenones A–D (**229**), 11β-hydroxyspongi-12-en-16-one (**230**), spongian-16-one (**221**)	Cytotoxic to murine lymphoma L1210 and KB cells	[154]
*Doriprismatica (Glossodoris) atromarginata*	Spongiadiol (**35**), spongiadiol diacetate (**231**), epispongiadiol (**232**), 12-deacetoxy-12-oxodeoxoscalarin (**136**), heteronemin (**233**), mooloolabene D (**234**)	Cytotoxic to MCF-7 breast cancer cells	[180,182,381,828]
*Felimida (Glossodoris) dalli, Doriprismatica (Glossodoris) sedna, Glossodoris rufomarginata, G. pallida, G. vespa, Ardeadoris (Glossodoris) averni*	12-deacetyl-23-acetoxy-20-methyl-12-epi-scalaradial (**135**)	Inhibition of mammalian phospholipase A2	[175,381]
*Hypselodoris infucata*	(–)-Furodysinin (**48**)	Cytotoxic to HeLa cell	[214]
*Felimida (Chromodoris) macfarlandi*	Macfarlandin E (**235**)	Golgi-modifying properties	[139,140,149,543]
*Hexabranchus sanguineus*	Ulapualides A (**190**), B (**236**) and C (**237**), kabiramides A (**238**), B (**239**), C (**62**), D (**240**), E (**241**) and G (**243**), dihydrohalichondramide (**63**), 33-methylhalichondramide (**242**), halichondramide (**244**), Hurghadin	Cytotoxic to murine L1210 leukemia cells, cytotoxic to several HTCLs, and cytotoxic to human MCF-7 breast cancer cells	[545]
*Jorunna funebris*	Jorumycin (**189**), jorunnamycins A–C (**245**), renieramycin M (**246**)	Cytotoxic to cancer cell lines P388, A549, HT29, and MEL28, and cytotoxic to human colon (HCT-116) and breast (MDA-MB-435) cancer cells	[560]
*Jorunna funebris*	Fennebricins A and B, N-formyl-1,2-dihydrorenierol	Strong NF-κB inhibition, and cytotoxic to A549 and HL-60 tumor cell lines	[564,565]
*Peltodoris atromaculata*	Petroformynes (**247**), hydroxyl-dehydroisofulvinol (**248**), fulvinol	Cytotoxic to murine P388 leukemia cells, A549 NSCLC, HT-29 colon cancer and SKMEL-28 melanoma cells	[567,568]
*Halgerda aurantiomaculata*	Zooanemonin (**367**)	Antineoplastic	[829]
*Tambja capensis, T. ceutae, T. eliora, T. morosa, T. stegosauriformis, T. verconis, Roboastra tigris, Nembrotha spp.*	Tambjamines (**65**–**70**), tambjamine K (**249**), tetrapyrrole (**72**)	Cytotoxic to several tumor cell lines (Caco-2 colon cancer cells, HeLa cervix cancer cells)	
*Tritonia sp.*	Punaglandins (**250**)	Cytotoxic	[587]
*Doto pinnatifida*	Dotofide (**251**)	Cytotoxic to Hs683 oligodendroglioma, U373 glioblastoma, A549 NSCLC human carcinoma, MCF-7 breast carcinoma, SKMEL-28, and mouse B16F10 cells	[243,505]
*Tritoniopsis elegans*	Tritoniopsins A–D (**75**–**78**)	Cytotoxic to rat cell lines	[234]
*Dermatobranchus ornatus*	Ophirin (**187**)	Cytotoxic	[22]
*Leminda millecra*	Prenylated hydroquinone (**252**)	Cytotoxic to WHCO1, WHCO6 esophageal cancer cell lines	[588,589,590]
*Hermissenda crassicornis*	L-6-bromohypaphorine (**253**)	Cytotoxic to human a7 nicotinic acetylcholine receptor (nAChR subtype)	[591]
*Phyllodesmium briareum*	Brianthein W (**254**), excavatolide C (**255**)	Cytotoxic to cancer cell line P-388	[248]
*Phyllodesmium magnum*	11-episinulariolide acetate (**256**)	Cytotoxic to cancer cell line P-388	[248]
*Phyllodesmium longicirrum*	Trocheliophorol (**84**)	Cytotoxic	[245]
*Phidiana militaris*	Phidianidines A (**257**) and B	Cytotoxic to C6 and HeLa tumor cells	[592,593,595]
**Pleurobranchoidea (2)**			
*Pleurobranchus albiguttatus, P. forskalii*	Chlorolissoclimide (**258**), dichlorolissoclimide (**259**), haterumaimides A (**260**), C (**267**), J (**265**), K (**266**), G (**268**), I (**269**), 3ß-hydroxylissoclimide (**261**)	Cytotoxic to melanoma cells	[610,619]
*P. forskalii*	Keenamide A (**262**), cycloforskamide (**263**), ergosinine (**264**)	Cytotoxic to P-388, A-549, MEL-20, and HT-29 tumor cell lines	[611,612]
**Tylodinoidea (1)**			
*Tylodina perversa*	Iso-fistularin 3 (**270**)	Cytotoxic to human HeLa cervix carcinoma cells	[257,625]
**Cephalaspidea (2)**			
*Philinopsis speciosa*	Kulolides 1 (**271**), 2 (**272**) and 3 (**273**), kulokainalide 1 (**274**), lulokekahilides 1 (**275**) and 2 (**276**)	Cytotoxic to L-1210, P388 leukemia, human SK-OV-3 ovarian, tMDA-MB-435 breast cancer, human A549 NSCLC, K562 chronic myelogenous leukemia, HeLa cervix carcinoma, and MCF-7 breast cancer cell lines	[277,626,627,628]
*Scaphander lignarius*	ARA, EPA, HTA (fatty acids)	Cytotoxic to a set of cancer and normal cell lines	[630]
**Anaspidea (11)**			
*Aplysia angasi, A. dactylomela, A. depilans, A. fasciata, A. juliana, A. kurodai, A. oculifera, A. punctata*	Aplysistatin (**277**)	Cytotoxic to mouse P388 leukemia, human KB cancer, and HeLa cervix carcinoma cells	[613,631,820]
*Aplysia dactylomela*	Elatol (**278**), obtusol (**279**), iso-obtusol (**280**), linear halogenated monoterpene (**281**)	Cytotoxic to ten cancer cell lines, B16F10 melanoma, HM02 gastric carcinoma, HEP-G2 liver carcinoma, and MCF-7 breast carcinoma cancer cells	[631,633,632,633]
*A. dactylomela*	Parguerol (**282**), parguerol-16-acetate (**283**), iso-parguerol (**284**), iso-parguerol-16-acetate (**285**), deoxyparguerol (**286**)	Cytotoxic to P388 leukemia and Ehrlich ascite carcinoma cells	[611,636,640]
*A. dactylomela*	Aplysqualenol A (**287**)	Cytotoxic to 60 cancer cell lines	[641,642]
*A. dactylomela*	Thyrsiferol (**288**)	Cytotoxic to P388 leukemia and T47D human breast tumor cells, and suppression of hypoxic induction of HIF-1 target genes	[647,648,649]
*Aplysia depilans*	Endoperoxide sterol (**289**)	Cytotoxic to human HCT-116 colorectal cancer cells	[650,651]
*Aplysia fasciata*	3-epi-aplykurodinone B (**145**)	Cytotoxic to mouse P388 leukemia, human A549 NSCLC, HT-29 colon cancer, and SKMEl-28 melanoma	[397]
*Aplysia juliana*	Pyropheophorbides a and b, julianin S	Cytotoxic	[288,432]
*Aplysia kurodai*	Aplyronines A (**191**), B (**290**), C (**291**), D (**292**) and H (**293**), aplaminal (**294**)	Cytotoxic to human HeLa S3 cervix carcinoma cells	[653,654,657,658]
*A. kurodai*	Aplaminone (**295**), neoaplaminone (**296**), neoaplaminone sulfate (**297**)	Cytotoxic to human HeLa S3 cervix carcinoma cells	[667]
*A. kurodai*	Aplysiaterpenoid A (**298**), aplysiapyranoids A–D (**299**)	Cytotoxic to Vero, MDCK, and B16 cells	[668,669]
*A. kurodai*	Kurodainol (**300**), aplysiaterpenoids A–D (**298**), aplysin-20 (**301**), iso-aplysin-20, aplysiadiol (**302**), epi-aplysin-20, ent-isoconcinndiol (**303**), aplysianin A	Induction of growth inhibitory effects in various cancer cell lines	[668,669,670,671,672,673,674]
*A. kurodai*	(-)-Aplysin (**304**), aplysinol (**305**), aplykurodins A (**306**) and B (**146**)	Cytotoxic to various cancer cell lines, human A549 NSCLC, and human glioma cells	[680,682,683]
*Aplysia oculifera*	Oculiferane (**308**), epi-obtusane (**309**)	Cytotoxic to PC-3 prostate, A549 NSCLC, MCF-7 breast, HepG2 liver, and HCT116 colon cancer cell lines	[684]
*Aplysia punctata*	Halogenated monoterpenes (**310**–**312**)	Cytotoxic to four tumor cell lines	[685]
*Dolabella auricularia*	Dolabellanin A	Antineoplastic	[435]
*D. auricularia*	Auripyrones A (**319**) and B, dolabelides A (**320**), B (**321**), C (**322**) and D (**323**)	Cytotoxic to human HeLa S3 cancer cells	[687,696,701,702]
*D. auricularia*	Doliculide (**325**)	Cytotoxic to human HeLa S3, MCF-7, and MDA-MB-231 breast cancer cells	[708]
*D. auricularia*	Dolastatins 3 (**192**), 10 (**326**), 11 (**327**), 12 (**328**), 13 (**329**), 14 (**330**), 15 (**331**), 16 (**346**), 17 (**347**), 18 (**348**), 19 (**332**), C (**333**), D (**334**), G (**337**) and H (**335**), iso-dolastatin H (**336**), debromoaplysiatoxin (**97**), anhydrodebromo-aplysiatoxin, aurilide (**324**), nor-dolastatin G (**338**), auristatins E (**339**), PHE (**340**), PYE (**341**), 2-AQ (**342**), 6-AQ (**343**) and PE (**344**), tasidotin (**345**)	Cytotoxic to renal, ovarian, prostate, hepatobiliary, pancreatic cancer cell lines, P388 murine leukemia, colon 26 cancer, Lewis lung carcinoma, B16 melanoma, M5076 sarcoma, human MX-1 breast cancer, LX-1, MCF-7, colon KM20L2 cancer, and SBC-3 SCLC cell lines	[46,688,694,703,705,717,718,719,721,722,724,744,750,751,766]
*Stylocheilus longicauda*	Aplysiatoxin (**96**), debromoaplysiatoxin (**97**), makalika (**99**), makalikone (**100**), lyngbyatoxin A (**349**), lyngbyatoxin A acetate (**101**), malyngamide B, O (**102**) and P (**103**)	Cytotoxic to P388, A549, HT29, and HTB38 cancer cell lines, and toxic to mice	[302,304,305,307,777,778,779]
*Bursatella leachii*	Lyngbyatoxin A (**349**), debromoaplysiatoxin (**97**), hectochlorin (**351**), deacetylhectochlorin (**352**), malyndamides S (**350**) and X (**353**)	Cytotoxic to murine P388 leukemia, human A549, NSCLC, NCI-H187 (SCLC), HT-29 colon cancer, HL60 leukemia, KB, and BC breast cancer	[783,784,785,786,787]
**Sacoglossa (3)**			
*Elysia subornata*	Caulerpenyne (**155**)	Cytotoxic to neuroblastoma SK-N-SH cell line	[316,411,788,789,790]
*Elysia rufescens*	Kahalide F (**194**), iso-kahalalide F (**354**)	Cytotoxic to A549 and Hs683 cell lines, and breast cancer cell lines SKBR3 and BT474	[331,797,800]
*Elysia ornata*	Kahalides F (**194**), Z_1_ (**356**), Z_2_ (**355**), elisidepsin (**356**)	Cytotoxic to A549 and Hs683, breast, colon, head, neck, lung, ovary, pancreas, prostate, and melanoma cell lines	[795,797,810,811,812]
**Pulmonata (9)**			
*Trimusculus peruvianus *	Hydroxylated sterol (**358**)	Cytotoxic to human HCT-116 and HT29 colon cancer cell lines	[819]
*Trimusculus costatus*	Secosterol (**359**)	Cytotoxic to WHCO1 esophageal cancer cell line	[337]
*Siphonaria capensis, S. concinna, S. cristatus, S. serrata*	Siphonarienfuranon, capensinone, denticulatins	Cytotoxic	[339,346,352]
*Siphonaria spp.*	Siphonarienolone (**119**), diemenensins A (**165**) and B, siphonarin A (**120**), vallartanones A and B	Cytotoxic	[27,134,340,341,342,343,344,348,351]
*Onchidium sp.*	onchidin (**121**), onchidin B (**360**), onchidione (**122**), onchidiol (**361**), ilikonapyrones (362,363), onchidionol	Cytotoxic to murine P388 and KB oral cancer cells, and regulation of some genes related to tumor growth	[131,358,359,821]

**Table 11:** Number of antibiotic compounds in the different heterobranch groups. In brackets: number of species with antibiotic compounds, number of the compounds in figures, and reference numbers. **#** Number.

Species (#)	Compounds (#)	Activity	References (#)
**Nudibranchia (21)**			
*Phyllidiella pustulosa*	Axisonitrile-3 (**25**)	Antimycobacterial activity against Mycobacterium tuberculosis	[427]
*P. pustulosa, P. coelestis*	Xidaoisocyanate A (**24**)	Antibiotic activity	[117]
*Phyllidia picta*	Pictaisonitrile 1 (**23**) and 2	Antibiotic activity	[112]
*Phyllidia varicosa*	9-Thiocyanatopupukeanane (**126**)	Antibiotic activity	[247]
*Phyllidiella rosans*	9-Isocyanopupukeanane (**21**), epi-9-isocyanopupukeanane	Antibacterial activity against *Bacillus subtilis* and *Candida albicans*	[110]
*Doriorismatica (Glossodoris) atromarginata*	Scalaranes, heteronemin (**233**)	Antimycobacterial activity against *M. tuberculosis* H_37_Rv	[833]
*Glossodoris hikuerensis, G. vespa, G. cincta*	Heteronemin (**233**), scalaradial (**44**), 12-deacetoxy-12-oxoscalaradial (**43**), 12-deacetoxy-12-oxo-deoxoscalarin (**136**), 12-epi-scalaradial	Antibiotic activity	[178]
*Felimida (Chromodoris) macfarlandi*	Macfarlandines D and E (**235**)	Antibacterial activity against *B. subtilis* in the disk assay system at 10 gg per disk, and activity against *Vibrio anguillarum* and Beneckea harveyi at 100 gg per disk	[139,140,149,543]
*Chromodoris willani*	Deoximanoalide (**364**), deoxysecomanoalide (**365**)	Antimicrobial activity against *Escherichia coli* and *B. subtilis*, and inhibitor of snake venom phospholipase A2	[159]
*Chromodoris spp.*	Nakafuran-8 (**54**), nakafuran-9 (**51**), puupehenone (**218**)	Antibacterial activity against *E. coli*, *Staphylococcus aureus*, *Pseudomonas aeruginosa*, *B. subtilis*, and antifungal activity against *C. albicans*	[157,158,165]
*Hexabranchus sanguineus*	Kabiramides A–E (**238**, **239**, **62**, **240**, **241**), sanguinamides A (**64**), B (**366**), halichondriamides (**244**), ulapualides A (**190**) and B (**236**)	Antibacterial activity against *P. aeruginosa*, and antifungal activity against *C. albicans*	[208,219,221,222,545,834,835,836,837]
*Jorunna funebris*	Jorumycin (**189**), jorunnamycins A–C (**245**)	Antimicrobial activityagainst *B. subtilis* and *S. aureus*	[166,553,560]
*Halgerda aurantiomaculata*	Zooanemonin (**367**)	Antibacterial	[841]
*Roboastra tigris, Tambja abdere, T. eliora*	Tambjamines (**65**–**70**, **249**), tetrapyrrole (**72**)	Antibacterial activity against *B. subtilis*	[223]
*Leminda millecra*	Millecrones A (**368**) and B (**369**), millecrols A (**370**) and B (**371**)	Antibiotic activity against *C. albicans*, *S. aureus* and *B. subtilis*	[588]
*Dermatobranchus otome*	DO1 (**372**), DO2 (**373**), DO3 (**374**)	Antibacterial activity against *B. subtilis*	[842]
*Armina babai*	Extracts	Antibacterial activity against *Pseudomonas* sp. and *Proteus mirabilis*	[843]
**Tylodinoidea (1)**			
*Tylodina corticalis*	Hexadellin (**375**), aplysamine 2 (**376**)	Antibacterial activity against *E. coli* and *S. aureus*	[262]
**Anaspidea (5)**			
*Aplysia punctata*	Glandulaurencianols A–C (**162**,**163**), punctatol (**164**)	Antibacterial activity against *B. subtilis* and *E. coli*	[429,430,431]
*Aplysia juliana*	Julianin-S	Antibacterial activity	[288]
*Aplysia kurodai*	Aplysianin E	Antifungal activity against *C. albicans*	[672,673,674]
*Dolabella auricularia*	Dolabellanin A	Antibacterial activity against *E. coli*	[435]
*Bursatella leachii plei, B. leachii savignyana*	Bursatellin (**105**)	Antibiotic activity	[311,312]
**Sacoglossa (4)**			
*Elysia rufescens*	Kahalalides A (**377**) and F (**194**), iso-kahalalide F (**354**)	Antimycobacterial activity against *Mycobacterium tuberculosis* and *M. intracellulare*	[794]
*Elysia ornata, E. grandifolia*	Kahalalide F (**194**)	Antimycobacterial activity against *M. tuberculosis* and *M. intracellulare*	[331,411,797]
*Cyerce nigricans*	Chlorodesmin (**114**)	Antibacterial and antifungal activity	[845,846]
**Pulmonata (7)**			
*Siphonaria australis, S. diemenensis, S. capensis, S. concinna, S. cristatus, S. serrata, S. pectinata*	Siphonarienolone (**119**), siphonarin A (**120**), pectinatone (**166**)	Antimicrobial activity	[340,341,342,343,346]

**Table 12:** Antiparasitic compounds in the different heterobranch groups. In brackets: number of species with antiparasitic compounds, number of the compounds in figures, and reference numbers. **#** Number.

Species (#)	Compounds (#)	Activity	References (#)
**Nudibranchia (4)**			
*Phyllidiella pustulosa*	Axisonitrile-3 (**25**), pustulosaisonitrile-1 (**378**), 10-thiocyano-4-cadinene (**383**)	Activity against *Plasmodium falciparum*	[118,123,848,849,850]
*Phyllidia ocellata*	2-*Iso*cyanoclovene (**379**), 2-*iso*cyanoclovane (**380**), 4,5-*epi*-10-*iso*cyanoisodauc-6-ene (**381**), 1-*iso*thiocyanatoepicaryolane (**382**)	Activity against *Plasmodium falciparum*	[376]
*Notodoris gardineri*	*Iso*-naamidine-A (**160**), dorimidazole A (**386**)	Anthelminthic activity	[424,426]
*Chromodoris lochi*	Mycothiazole (**129**)	Anthelminthic and toxic activity	[378]
**Sacoglossa (3)**			
*Elysia rufescens*, *E. ornata*, *E. grandifolia*	Kahalalides (**194**,**354**–**356**,**377**)	Antileishmanial activity	[794,795,796,797]

**Table 13:** Antiviral compounds in the different heterobranch groups. In brackets: number of species with antiviral compounds, number of the compounds in figures, and reference numbers. # Number.

Species (#)	Compounds (#)	Activity	References (#)
**Nudibranchia (7)**			
*Cadlina luteomarginata*	Ansellone A (**216**)	Activation of the latent proviral HIV-1 gene expression	[855]
*Chromodoris mandapamensis, Glossodoris cincta*	Spongiadiol (**35**), *epi*-spongiadiol (**232**)	Activity against herpes simplex virus, type 1 (HSV-1) and P388 murine leukemia cells	[166,535]
*Chromodoris hamiltoni*	Latrunculins A (**38**) and B (**37**)	Activity against HIV-1	[153,155]
*Chromodoris africana, C. quadricolor*	Latrunculin B (**37**)	Activity against HIV-1	[155,853]
*Chromodoris petechialis*	Puupehenone (**218**)	Anti-HIV-1	[797]
**Anaspidea (1)**			
*Dolabella auricularia*	Dolastatin 3 (**192**)	Activity against HIV life cycle	[718,728,853]
**Sacoglossa (3)**			
*Elysia rufescens, E. grandifolia, E. ornata*	Kahalalide F (**194**), *iso*-kahalalide F (**354**)	Activity against herpes simplex virus II	[331,794,795,797,853]

**Table 14:** Anti-inflammatory compounds in the different heterobranch groups. In brackets: number of species with anti-inflammatory compounds, number of the compounds in figures, and reference numbers. **#** Number.

Species (#)	Compounds (#)	Activity	References (#)
**Nudibranchia (11)**			
*Glossodoris rufomarginata, G. pallida, G. vespa, G. averni, G. hikuerensis, G. atromarginata, G. cincta*	Scalaradial (**44**)	Potent inhibition of PLA_2_, and potent anti-inflammatory activity	[175,177,381,383,856,857,858]
*Goniobranchus splendidus*	Gracilins (**224–228**)	Cyclosporine A mimics, BACE1 and ERK inhibition	[190,533,534]
*Tethys fimbria, Melibe viridis*	Prostaglandin E-1,15-lactones (**80**, **81**)	Reduction of inflammation after autotomy and tissue regeneration	[77,240]
*Tritonia* sp.	Punaglandins (**250**)	Anti-inflammatory activity	[587]
**Anaspidea (3)**			
*Aplysia depilans*	Carotenoids, polyunsaturated fatty acids	Anti-inflammatory activity	[859]
*Aplysia dactylomela*	Dactyloditerpenol acetate (**385**)	Anti-neuroinflammatory activity	[860,861]
*Bursatella leachii*	Malyngamide S (**350**)	Anti-inflammatory activity	[786]

**Table 15:** Compounds used against neurodegenerative diseases in the different heterobranch groups. In brackets: number of species with these compounds, number of the compounds in figures, and reference numbers. **#** Number.

Species (#)	Compounds (#)	Activity	References (#)
**Nudibranchia (4)**			
*Polycera atra*	Bryostatin 1 (**203**)	Alzheimer disease (AD)	[492,493,494,866]
*Goniobranchus obsoletus, G. splendidus*	Gracilins (**224**–**228**)	Potential against neurodegenerative diseases	[533,534,863]
*Cadlina luteomarginata*	Ansellone A (**216**)	cAMP activation (neurodegenerative diseases)	[513]
**Cephalaspidea (1)**			
*Scaphander lignarius*	Lignarenone B (**171**)	Alzheimer disease (AD)	[864,867]

**Table 16:** Other pharmacological activities in compounds from different heterobranch groups. In brackets: number of species with active compounds, number of the compounds in figures, and reference numbers. **#** Number.

Species (#)	Compounds (#)	Activity	References (#)
**Nudibranchia (1)**			
*Janolus cristatus*	Janolusimide (**138**)	Toxic to mice	[390,388]
**Pleurobranchoidea (1)**			
*Pleurobranchaea maculata*	Tetrodotoxin (TTX) (**387**)	Neurotoxin	[869,870]

## Abbreviations

AD	Alzheimer Disease
ADCs	Antibody-drug conjugates
ADMET	Absortion, Distribution, Metabolism, Excretion, and Toxicity
ADR	Adriamycin resistant
BGCs	Biosynthetic Gene Clusters
cAMP	Cyclic Adenosine Monophosphate
EC_50_	Half maximal Effective Concentration
ED_50_	Half effective dose
EGF	Epidermal Groth Factor
EGFR	Epidermal Groth Factor Receptor
ERK	Extracellular signal-regulated kinases
FDA	Food and Drugs Administration
GI_50_	Maximal inhibition of cell proliferation
HCCLs	Human Colon Cancer Cell Lines
HEL	Human Erythroleukemia cells
HeLa	Henrietta_Lacks cell line from cervical cancer cells
HIF-1	Hypoxia Inducible Factor 1
HTCLs	Human Tumor Cell Lines
KB	Subline of the KERATIN-forming tumor cell line HeLa
IC_50_	Half minimal Inhibitory Concentration
LD	Lethal Dose
LRA	Latency Reversal Agent
MAAs	Mycosporine-like Amino Acids
MAPK	Mitogen-Activated Protein Kinase
MDA	Microtubule-Desestabilizing Agent
MDFs	Mantle Dermal Formations
MDR	Multidrug resistant variant
MIC	Minimum Inhibitory Concentration
MNPs	Marine Natural Products
MTT	Dimethyl Thiazolyl Diphenyl Tetrazolium Bromide
NCI	National Cancer Institute
NPs	Natural Products
NSCLC	Nonsmall Cell Lung Cancer
PBM	Peripheral Blood Mononuclear
PG	Prostaglandins
PKC	Protein Kinase C
PSMA-ADC	Prostate-specific membrane antigen antibody–drug conjugate
PTPRK	Protein Tyrosine Phosphatase Receptor type K
TRAIL	Tumor necrosis factor-related apoptosis-inducing ligand
TTX	Tetrodotoxin
UVR	Ultra-Violet Radiation
VCR	Vincristine resistant
